# Supplementary material for: Prognostic tools and candidate drugs based on plasma proteomics of patients with severe COVID-19 complications
Source: Nat Commun. 2022 Feb 17;13:946. doi: 10.1038/s41467-022-28639-4 (PMC8854716; doi:10.1038/s41467-022-28639-4)
Supplement: Supplementary file 1 — Supplementary Information [file 41467_2022_28639_MOESM1_ESM.pdf]

Prognostic tools and candidate drugs based on plasma proteomics of patients with severe COVID-19 complications

Maryam A.Y. Al-Nesf, Houari B. Abdesselem, Ilham Bensmail, Shahd Ibrahim, Walaa A.H. Saeed, Sara S.I. Mohammed, Almurtada Razok, Hashim Alhussain , Reham M.A. Aly, Muna Al Maslamani, Khalid Ouararhni, Mohamad Y. Khatib, Ali Ait Hssain, Ali S. Omrani, Saad Al-Kaabi, Abdullatif Al Khal, Asmaa A. Al-Thani, Waseem Samsam, Abdulaziz Farooq, Jassim Al-Suwaidi, Mohammed Al-Maadheedh, Heba H. Al-Siddiqi, Alexandra E. Butler, Julie V. Decock, Vidya Mohamed-Ali and Fares Al-Ejeh

Content

Supplementary Figures
- Supplementary Figure 1
- Supplementary Figure 2
- Supplementary Figure 3
- Supplementary Figure 4
- Supplementary Figure 5
- Supplementary Figure 6

Supplementary Note 1 & Supplementary References

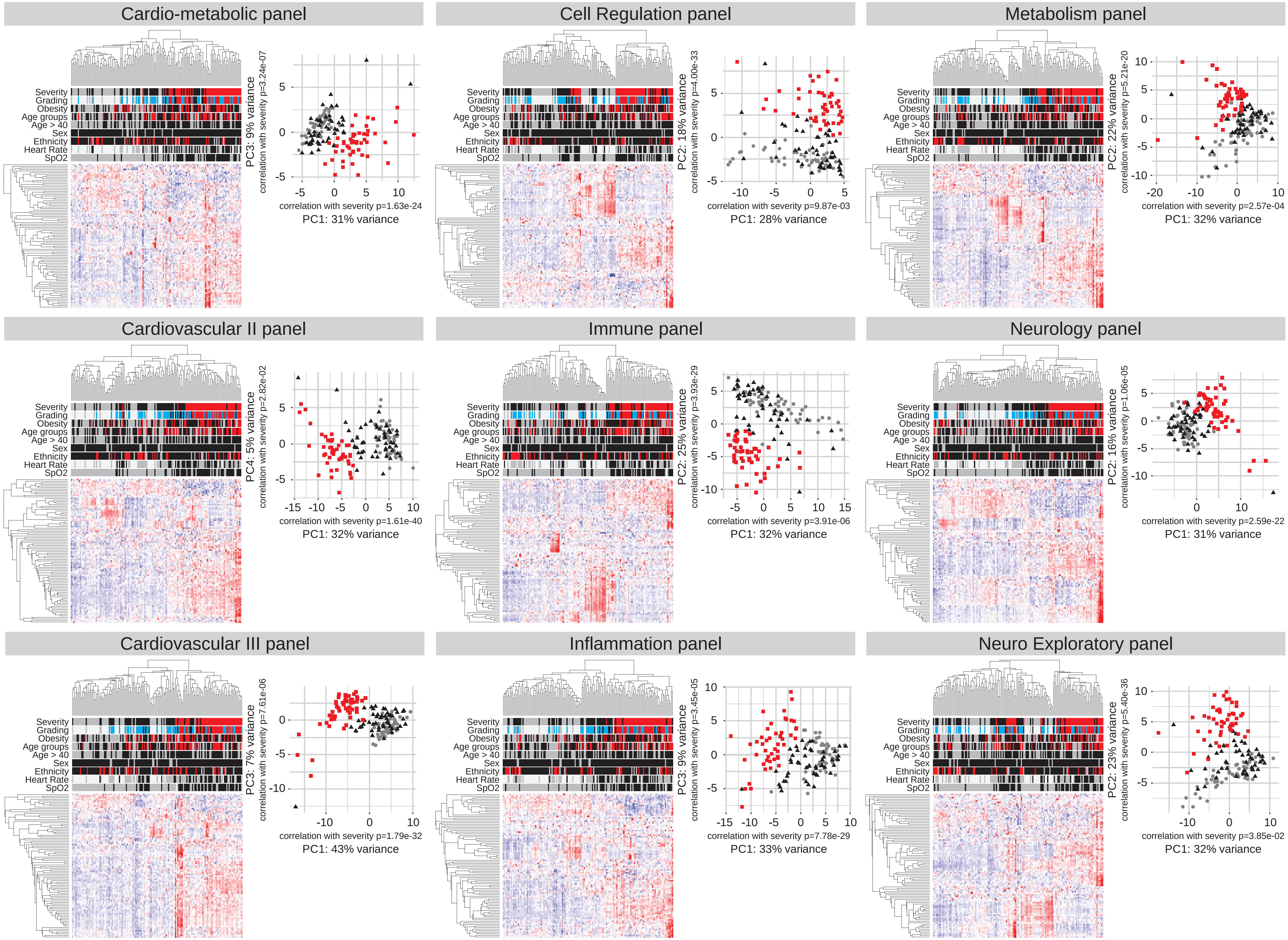

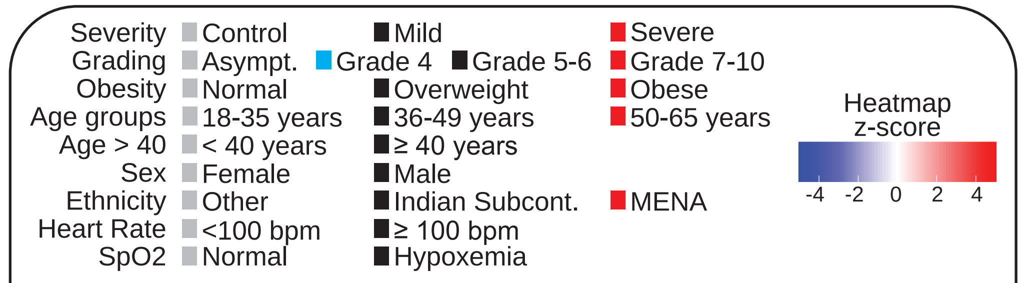

Supplementary Figure 1: Differential protein expression in plasma from patients with active SARS-CoV-2 infection. Unsupervised hierarchical clustering based of all 92 proteins in each of the named Olink panel showed a separation between patients with severe complications compared to mild cases and controls. The heatmap shows z-scores and clustering was done using correlation and average linkage. Principal component analysis (PCA) tested the separation of the severe cases based on the expression profiles of all proteins. The limma package was used to identify differentially expressed proteins (DEPs) from the single Olink panels defined as protein with more than 1.25-fold change with a p-value of <0.05 and FDR <0.1. Differential expression analysis addressed severity as the main effect and included obesity, age, sex, ethnicity, heart rate and SpO2 to correct for the interaction of these factors with disease severity. A summary of the number of DEPs for each panel is shown in Figure 1a.

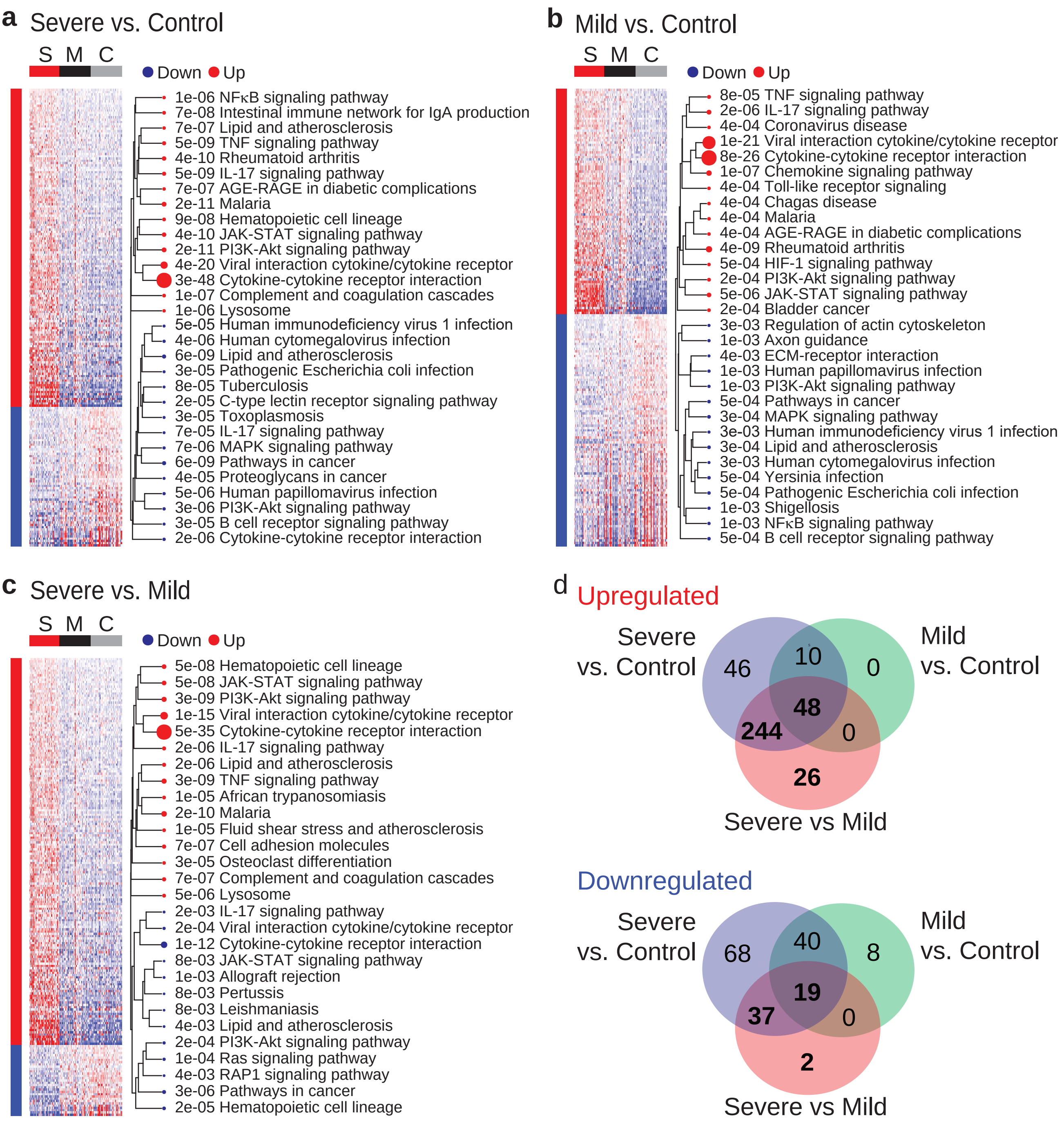

Supplementary Figure 2: Functional analysis of differentially expressed proteins in plasma of patients with active SARS-CoV-2 infection. (**a-c**) Heatmaps of the expression differentially expressed proteins (DEPs) in severe (S) and mild (M) cases and control (C) are shown to the side of enrichment trees of enriched KEGG pathways using DEP.92. Upregulated and downregulated proteins and pathways are shown in red and blue respectively. The p-value for enrichment is depicted by the size of the circles in the enrichment trees. (**d**) Venn diagrams summarizing the shared and unique upregulated (top) and downregulated (bottom) DEPs. The identities of the proteins in each Venn diagram are shown in Supplementary Data 3.

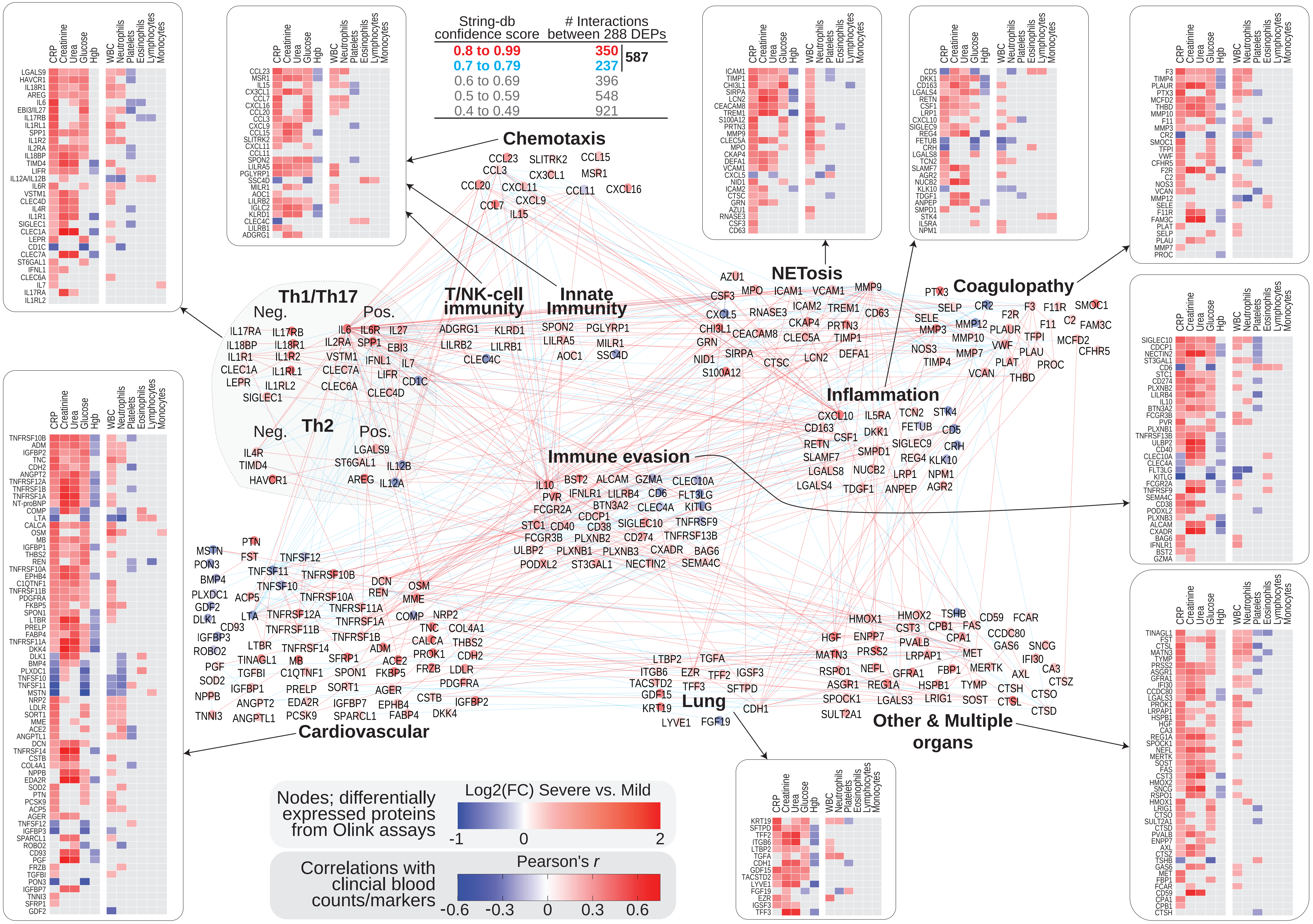

Supplementary Figure 3: Functional networks of deregulated plasma proteins in severe versus mild COVID-19 disease. Differentially expressed proteins (DEPs) in patients with severe complications compared to mild-moderate disease were subjected to network analysis using the STRING database and annotation for their function as circulating proteins (**Supplementary Data 3** and **Supplementary Notes**). Of the 375 DEPs (1.25-fold change in severe vs. mild cases), 288 (77%) DEPs could be allocated to 11 functional groups considering their potential function as circulating proteins; chemotaxis, coagulopathy/fibrinolysis, immune evasion, innate immunity, T- or NK-cell immunity, T-/Th-cells dysfunction, inflammation, neutrophils/neutrophil extracellular traps (NETosis), and organ damage (lung, cardiovascular or other and multiple organs). DEPs are classified as agonists (pos.) or antagonist (neg.) for the Th1/Th17 and Th2 immune responses. The color intensities (red: upregulated, blue: downregulated; legend) depict the log2 fold-change between severe and mild-moderate cases. Interactions between the 288 DEPs are shown only for those with STRING-db confidence score ≥ 0.7 are shown (587 high-confidence interactions). Inserted table in the Figure summarizes the number of interactions across the different STRING-db confidence scores (0.4 to 0.99). The heatmaps summarize the Pearson's correlation coefficient (r) for significant correlations ($p<0.05$, two-tailed, GraphPad Prism) between each protein in the functional networks and the clinical blood biochemical markers and blood cell counts available in our cohort. Refer to **Supplementary Data 3** for the correlation r values of all DEPs with the clinical markers.

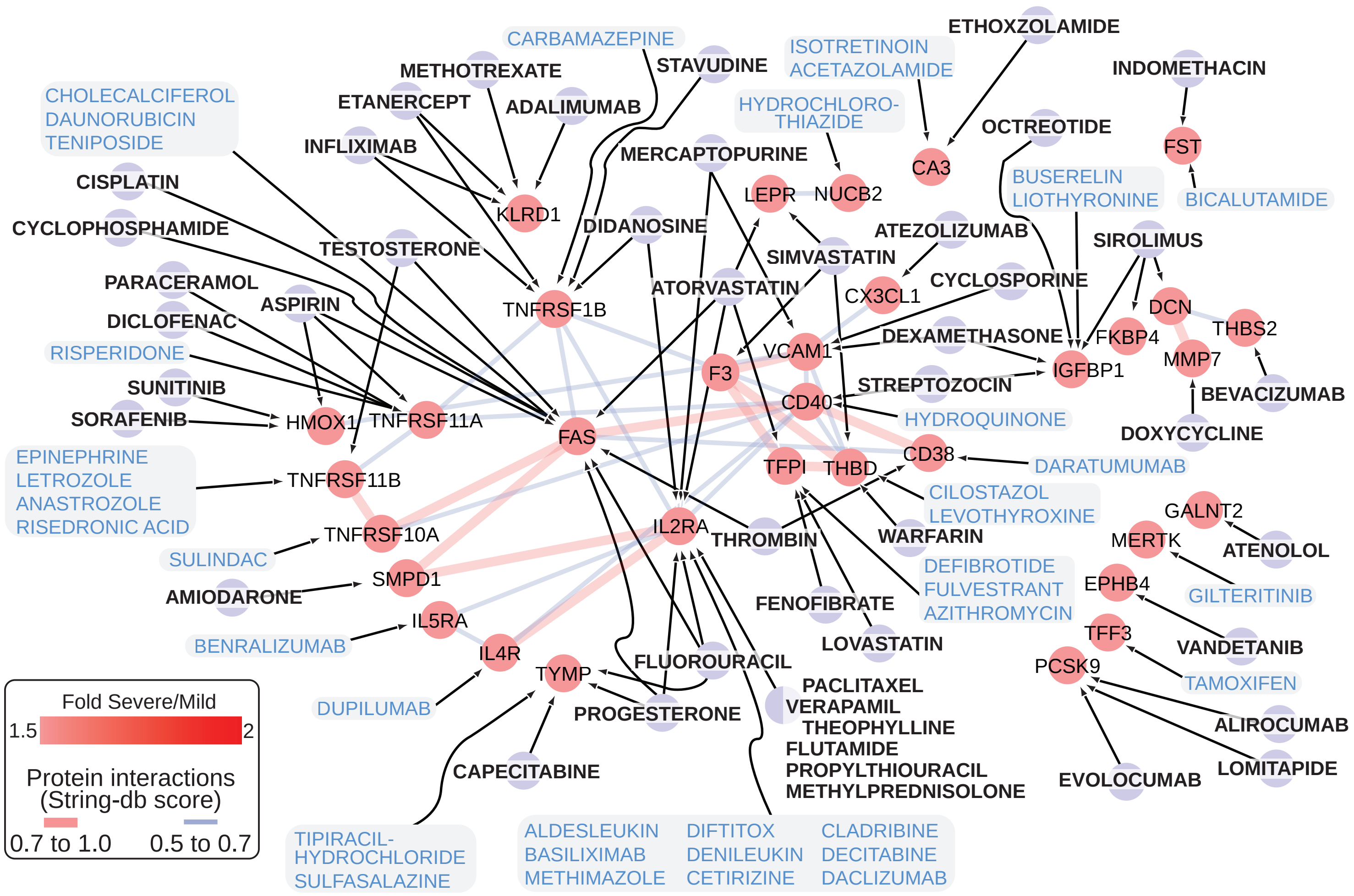

Supplementary Figure 4: Protein-drug interaction network of 1.5- to 2-fold upregulated plasma proteins in severe COVID-19. Proteins with 1.5- to 2-fold upregulation in patients with severe complications versus mild-moderate disease were subjected to protein-drug interaction (PDI) using the Drug-Gene Interaction database (DGIdb, v4.2.0). Target proteins are colored red, and the intensity depicts the fold-change. Drugs which target single proteins are shown in grey boxes and blue font and those that target multiple proteins (on this Figure or in Figure 4) are depicted in black font in blue nodes. Protein-protein interactions are colored according to the STRING-db confidence scores; red: confidence score ≥ 0.7, blue: confidence score ≥ 0.5 and < 0.7. Drugs in red bold font are notable examples discussed in the main text.

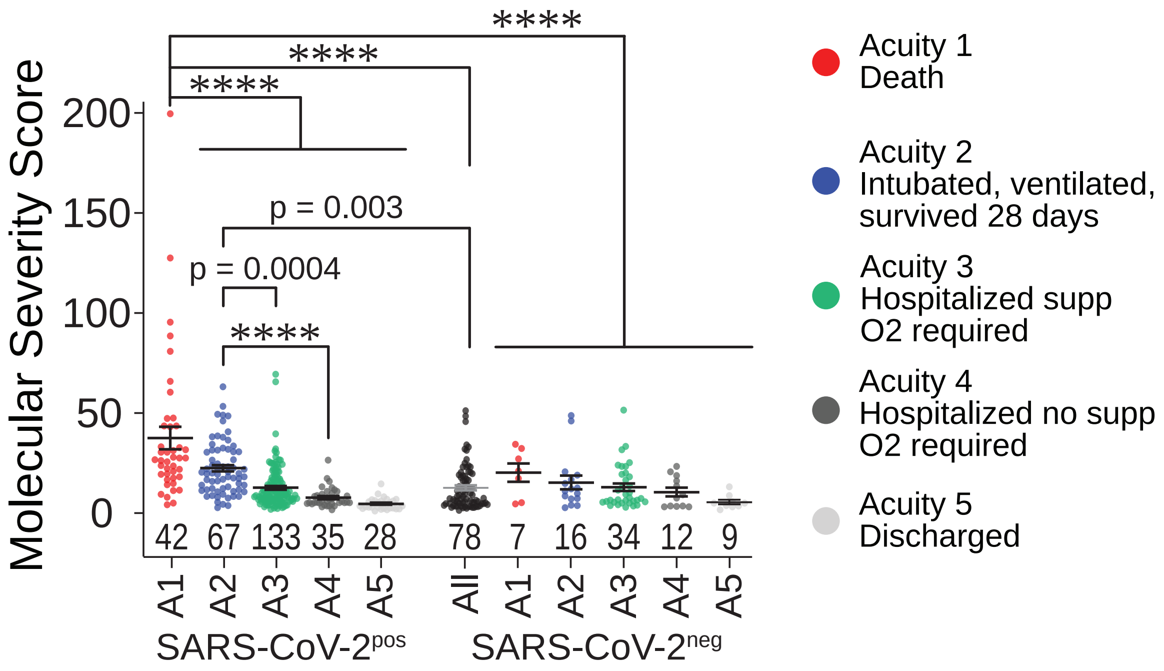

Supplementary Figure 5: The COVID-19 molecular severity score on day 0 in the SARS-CoV-2 positive and negative patients in Massachusetts General Hospital (MGH) cohort. The MGH cohort collected plasma samples on day 0 (within 24 hours of admission to the emergency department) from symptomatic patients, of whom 78 patients were found to be negative for SARS-CoV-2. The molecular severity score on day 0 was compared across the different severity levels (acuity max over 28-day period) and between SARS-CoV-2^{pos} and SARS-CoV-2^{neg} patients. Scatter plots show the calculated scores (mean ± SEM) and the number of patients in each group is stated under each plot. Only significant differences are depicted (two-way ANOVA with Tukey's multiple testing correction, GraphPad Prism); **** p<0.0001, exact p-values are stated otherwise.

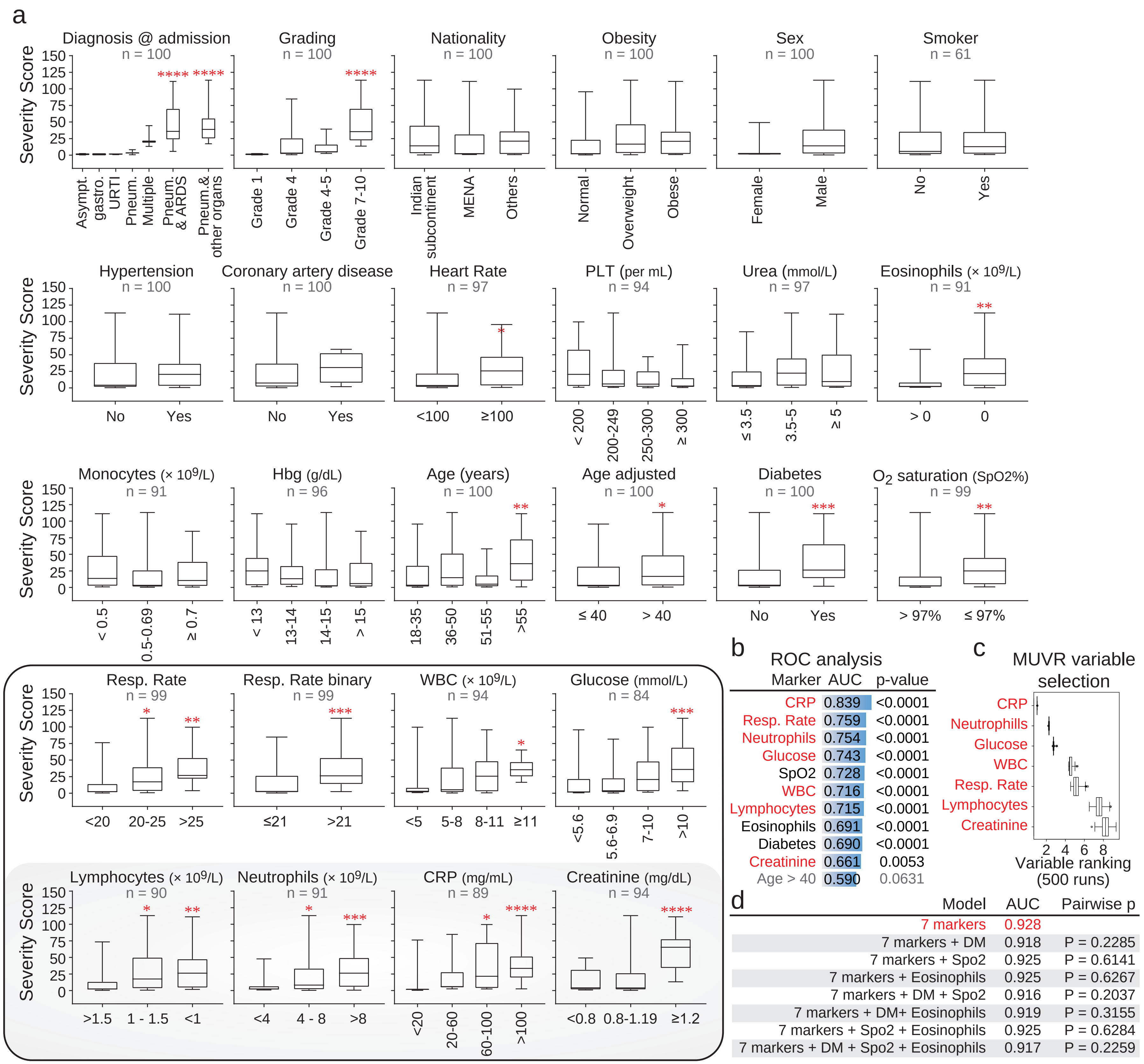

b ROC analysis

Marker	AUC	p-value
CRP	0.839	<0.0001
Resp. Rate	0.759	<0.0001
Neutrophils	0.754	<0.0001
Glucose	0.743	<0.0001
SpO2	0.728	<0.0001
WBC	0.716	<0.0001
Lymphocytes	0.715	<0.0001
Eosinophils	0.691	<0.0001
Diabetes	0.690	<0.0001
Creatinine	0.661	0.0053
Age > 40	0.590	0.0631

d

Model	AUC	Pairwise p
7 markers	0.928	
7 markers + DM	0.918	P = 0.2285
7 markers + Spo2	0.925	P = 0.6141
7 markers + Eosinophils	0.925	P = 0.6267
7 markers + DM + Spo2	0.916	P = 0.2037
7 markers + DM+ Eosinophils	0.919	P = 0.3155
7 markers + Spo2 + Eosinophils	0.925	P = 0.6284
7 markers + DM + Spo2 + Eosinophils	0.917	P = 0.2259

Supplementary Figure 6: Comparison of the COVID-19 molecular severity score across the groups within the clinical parameters included in the study cohort. (**a**) Boxplots (median as center line, box marks 25th and 75th percentiles, and whiskers define minimum and maximum) for the score from the 12-protein signature across the stated groups in each of the clinical annotations in infected patients (n = 100). One-way ANOVA with Dunnett's multiple testing correction was used for clinical parameters with more than two groups, and unpaired two- tailed t-test was used for parameters with two groups. Significant differences are depicted as * p<0.05, ** p<0.01, *** p<0.001 and **** p<0.0001. Refer to **Supplementary Data 6** for more details of the statistical comparisons and exact p-value. (**b**) ROC curve analysis of the parameters which showed significant association with the 12-protein molecular severity score. The DeLong et al. method was used for statistical analysis. (**c**) MUVR was used for variable selection using the same parameters in panel b. Seven parameters (markers) were selected by MUVR and the boxplot summarizes the median ranking (center line), 25th and 75th percentiles (box boundaries), and minimum and maximum (whiskers) from 500 independent MUVR runs. (**d**) The model of 7 markers from MUVR was further confirmed for performance using ROC curve analysis in comparison to models which included the remaining clinical markers. There was no additional benefit from addition diabetes, SpO2 and/or eosinophil counts as judged by pairwise comparisons (DeLong et al. method) against the model of the 7 markers alone. Abbreviations; Resp. Rate: Respiratory rate, WBC: white blood cells, CRP: C-reactive protein.

Supplementary Note 1

Functional annotation and literature for Figure 2 and Supplementary Data 3. The differentially expressed proteins in patients with severe COVID-19 versus patients with mild-moderate disease were subjected to functional annotation based on information from databases and literature and concerning their role in circulation and pathogenesis.

Protein	Annotation
**IL6**	In addition to a strong pro-inflammatory role, IL-6 can modulate the Th1/Th2 balance towards Th2, and with TGFβ it promotes Th17 cells [1]
**IL6R**	sIL-6R renders cells lacking the IL-6R, but expressing gp130, responsive to IL-6 [1]; the trans-signaling is highly inflammatory [2] and maintains local Th17 cells [3]
**EBI3/IL27**	Both form the IL27 which potentiate the early phase of Th1 response and suppress Th2 and Th17 differentiation [4]. IL27 is a potent immunosuppressant and increased in sepsis [5]
**IL12A/IL12B**	Growth factor and enhance lytic activity of activated T- and NK-cells and stimulate the production of IFNG by resting PBMC. Expressed by activated macrophages. Th1 cells development [6]. Th2 cells inhibit antigen-dependent IL-12 secretion by DCs [7]
**TIMD4**	Ligand for HAVCR1/TIM-1, expressed on APCs such as dendritic cells or macrophages. Also called TIM-4; Soluble form may be inhibitory of cellular function TIM-4 in Th2 development [8]
**HAVCR1**	Also called TIM-1, receptor for TIMD4. May be a receptor for SARS-CoV-2 in lung and kidney [9]. Soluble form may be inhibitory of cellular function TIM-1 [8] including its role in regulating Th2 responses [10]. Plasma TIM-1/KIM-1 associates with stroke [11] and lower kidney function [12]. Also elevated in cardiovascular disease, worsened diastolic function [13]
**LIFR**	Soluble form (sLIFR) inhibits LIF function [14] and binds to OSM. sLIFR may inhibit LIF-mediated promotion of Treg lineage and repressing Th17 lineage-specific genes [15]
**IL17RB**	Binds to IL17B and IL17E. Soluble form is a decoy receptor produced by Th2-skewed antigen-presenting cells (APC2) [16]
**IL17RA**	Receptor for IL17A and IL17F. Receptor for SARS coronavirus-2/SARS-CoV-2 virus protein ORF8 [17] and sIL17RA [18] acts a decoy receptor [17]
**CCL20**	Chemotaxis of DCs, effector/memory T-cells and B-cells, slightly, neutrophils, but not monocytes. Recruitment of proinflammatory IL17 producing Th17 and Treg cells to sites of inflammation [19]
**VSTM1**	VSTM1-v2 (soluble isoform) behaves as a cytokine, promoting IL17A secretion by CD4+ T-cells, and differentiation and activation of IL17 producing Th17 cells [20]
**IL1R1**	Mechanism for neutralization of IL1B by secreted/soluble receptors [21], which interferes with the critical role of IL-1 in Th17 differentiation [22, 23]
**IL1R2**	Secreted IL1R2 form is dominant mechanism for neutralization of IL1B by secreted/soluble receptors [21], as a decoy receptor it interferes with the critical role of IL-1 in Th17 differentiation [22, 23], thus implicated in several pathologies including sepsis [24]

**IL1RL1**	Receptor for IL33. Soluble form (sST2) inhibits IL33 binding and its cardioprotective effect [25] and is elevated in patients who do not survive from sepsis [26]. IL33 is an inducer of Th2 cells [27] which is inhibited by sST2.
**IL1RL2**	Receptor for IL36. IL36 signaling promotes Th1 polarization [28] but soluble receptor inhibits IL36 signaling [29]
**AREG**	Epithelial-derived AREG (Amphiregulin) can act to promote tissue repair and integrity. AREG is also secreted by innate lymphoid cells 2 (ILC2) and other innate immune cells and might be a critical component of type 2-mediated resistance and tolerance [30]. Pathogenic memory Th2 cells induce AREG via IL-33, which reprograms eosinophils that via Osteopontin/SPP1 facilitate an inflammatory state and airway fibrosis [31]
**IL18BP**	Inhibitor of IL18-mediated early Th1 cytokine response, IFNG production, resulting in reduced T-helper type 1 immune responses [32]
**IL18R1**	IL18 receptor involved in IL18-mediated IFNG synthesis from Th1 cells [33]. Soluble form inhibits IL18-mediated IFNG synthesis from Th1 cells [34, 35]
**SPP1**	Upregulate INFG and IL12, essential in the pathway that leads to type I immunity and Th1-cytokine functions [36]. Also involved in pathogenic memory Th2 cells induce AREG via IL-33, which reprograms eosinophils that via Osteopontin/SPP1 facilitate an inflammatory state and airway fibrosis [31]. SSP1 is elevated in several cardiovascular pathologies [37]
**IL10**	Dramatic early proinflammatory IL-10 elevation may play a pathological role in COVID-19 severity proinflammation and T-cell exhaustion [38]
**PVR**	Also called CD155 expressed in peripheral tissue (e.g., endothelial, epithelial cells and APCs) and is the ligand for CD226 (DNAM-1) expressed on NK cells, and a subset of T-cells (stimulatory) and to TIGIT on NK-/T-cells (inhibitory) [39]. sCD155 inhibits NK-Cells CD226 mediated cytokine production, including that of IL2, IL5, IL10, IL13, and IFNG cytotoxicity [40]
**ADGRG1**	Receptor involved in cell adhesion and probably in cell-cell interactions. ADGRG1 (GPR56) inhibits NK-cell cytotoxicity and is cleaved/shed (sGPR56) upon activated [41]
**LGALS9**	By interacting with TIM3, it inhibits Th 1 and Th17 cells, but not on Th2 cells, skewing to Th2 imbalance and Tregs [42, 43]. Binding to CD40 inhibits the proliferation and survival of CD4loCD40+ effector T-cells [44]
**BST2**	Tetherin is induced by INFa [45, 46] and restricts cell-free virions spread by blocking the release of envelop virus including SARS-CoV1. Plasma BST2 has been described in colorectal and breast cancers [47, 48]. SARS-CoV-2 Orf7a impedes Tetherin, and both are secreted in virus like particles as an evasion mechanism [49]
**SIGLEC10**	Immunoregulatory role, inhibiting pathogen-related and damage-associated molecular patterns (DAMPs)-mediated inflammation [50]. Siglec-10 Sv2, a secreted form, is the most abundantly expressed transcript in PBMC and retains functionality [51]; thus, might be an evasion mechanism allowing viral spread and excessive inflammation
**STC1**	Despite its anti-inflammatory role, it is a phagocytosis checkpoint driving immune evasion by binding to calreticulin; thus, abrogating membrane calreticulin-directed phagocytosis by APCs (macrophages and DCs) impairing APC capacity of antigen presentation and T cell activation [52]
**ST3GAL1**	ST3Gal-1 in circulation is principally carried by platelets and released upon activation [53]. ST3GAL1-mediated O-linked sialylation of CD55 act as CD55-mediated immune evasion [54]

**LILRB4**	Binds to MHC I molecules on APCs and NK-/T-cells and inhibits immune and inflammatory responses to regulate autoimmunity [55]. Soluble form is produced by splice variant [56]. Soluble form (sLILRB4) suppresses T cell responses and elicits T-cell anergy or activation of Treg or T suppressor cells [56-58]
**NECTIN2**	Also called PVRL2, expressed in peripheral tissue (e.g., endothelial, epithelial cells and APCs) and is the ligand for CD226 (DNAM-1) expressed on NK cells, and a subset of T-cells (stimulatory) and to PVRIG on NK-/T-cells (inhibitory) [39]. sNECTIN2 [59, 60]. Soluble form is inhibitory
**SIGLEC1**	Macrophage-restricted expressed resident and inflammatory macrophage mediating cell interactions to granulocytes, monocytes, NK-cells, B-cells and CD8 T-cells [61]. Soluble form (sSIGLEC1) associates with the INF type-I transcriptional signature and a biomarker of renal disease in SLE [62]. The soluble form is encoded by a variant transcript and is functional [63] which can be suppressive of function: reduce numbers of infiltrating Th1 and Th17 cell, higher numbers of Treg cells [64]
**PODXL2**	Cell surface transmembrane proteins ligand for vascular selectins mediates rapid rolling of leukocytes over vascular surfaces [65]. Cleaved [66, 67]; the soluble form is inhibitory [67] and would inhibit leukocytes recruitment site of injury during inflammation
**CD274**	PD-L1 modulates the activation threshold of T-cells and limits T-cell effector response. sPDL1 induced immune suppression and damage, and associates with COVID-19 pathogenesis and mortality [68]
**IFNL1**	INF lambda 1 (type-III INF, also called IL29) involved in antiviral host defense, predominantly released by epithelial tissues, including lung. Ligand for IL10RB and IFNLR1 leading to expression of IFN-stimulated genes (ISG). Significantly up-regulate IL6, IL8 and IL10 from monocytes [69] and inhibits Th2 polarization towards Th1 [70-72]
**IFNLR1**	IFNLR1/IL10RB dimer is a receptor for type III INFs mediating their antiviral activity. Expressed on epithelial cells within the lung, intestine, and liver [70, 72]. Soluble variant of IFNLR1 (sIFNLR1/sIFN-λR1), inhibits antiviral and immune effect of type III INF signaling/ISG induction [73]
**PGLYRP1**	Pattern recognition receptor in innate immunity, promotes the activation of monocytes/macrophages and enhances the inflammatory response [74]. Able to kill virus-infected cells [75]. High in cardiovascular disease and heart failure [76, 77]. It also binds to TNFRSF1A/TNFR1 and inhibit of TNFα cytotoxic activity [78]
**CD38**	Enzyme and moonlights as a receptor on immune cells (B-, T-, NK-Cells), upregulated by inflammatory mediators, and used as a cell activation marker [79]. Released to blood as a soluble form which inhibits binding to the membrane form, inhibiting adhesion to endothelial cells and immune cell chemotaxis [80] but retains its enzymatic function [81]
**IL2RA**	IL2 receptor regulates immune tolerance by controlling Tregs. Soluble form enhances the development of Th17 responses [82] and increased in association with cardiovascular events [83]. Soluble IL2RA (sCD25) is elevated in severe COVID-19 [84]
**LEPR**	Soluble LEPR inhibits Leptin's effects on enhancing the immune response via activating APCs, Th1/Th17 cells function and proliferation, and suppresses Th2 cytokine production [85, 86]

**CDCP1**	Cell adhesion and cell matrix association; ligand for CD6. Shedding and a soluble isoform leads to sCDCP1, occurs during tissue injury [87, 88]. sCDCP1 can bind CD6 [89], reduces Th1 and/or Th17 immune responses or acts as T-cell chemoattractant [90]. High sCDCP1 and sALCAM along with reduced sCD6 suggest reduced T-cell activation
**ALCAM**	Promotes T-cell activation and proliferation via interaction with CD6 but the soluble form (sALCAM) abolishes this function. High sCDCP1 and sALCAM along with reduced sCD6 suggest reduced T-cell activation. Shed by platelets [91]
**CD6**	Interaction with ALCAM/CD166 functions as costimulatory molecule; promotes T-cell activation and proliferation. Soluble CD6 (sCD6) - shedding - indicates activation of T-Cells but leads to inhibition of T-cells [92]. High sCDCP1 and sALCAM along with reduced sCD6 suggest reduced T-cell activation
**PLXNB1**	Plexin-Semaphorin. Expressed by activated T cells, immature bone marrow-derived DCs, and lung DCs [93] and platelets [94]. Plexin B1-B3 members have a convertase cleavage site [95]. sPLXNB1 (isoform or cleaved) may neutralize SEMA4D functions [96] which include T-cell priming, B-cell survival and antibody production in response to T-dependent antigens, monocyte paralysis and the arrest of its spontaneous and chemokine-induced migration [97]
**PLXNB2**	Plexin-Semaphorin. Expressed on macrophages, DCs and plasmacytoid DCs [95]. Receptor for SEMA4C. Negatively regulates macrophage migration. Optimal activation and differentiation of CD8+ T Cells [98]. Plexin B1-B3 members have a convertase cleavage site [95]. Cleavage releases sPLXNB2 [66] would interfere with CD8+ T-cell activation
**PLXNB3**	Plexin-Semaphorin. Disruption of focal adhesions and cellular collapse as well as inhibition of cell migration and invasion. Plexin B1-B3 members have a convertase cleavage site [95]. Shed by platelets [91]; soluble form would block SEMA5A effect of increased T- and NK-cell proliferation and induced the secretion of proinflammatory Th1/Th17 cytokines [99]
**SEMA4C**	Plexin-Semaphorin. Ligand for PLXNB2, required for Tfh cells to migrate to the GC and a marker of memory B-cells and B-cells stimulated by Th2 cytokines [100, 101]. Both soluble PLXNB2 and SEMA4C marks the block of their function
**RNASE3**	Released during degranulation/activated eosinophils [102, 103], and released in neutrophil NETs [104]. Cytotoxin activity [105] and may play a role in neutrophil transendothelial migration
**ULBP2**	Ligand for the NKG2D killer activation receptor on NK-cells mediating cytotoxicity and release multiple cytokines/chemokines. Soluble/secreted ULBP2 inhibits NK-cells as a mechanism to evade immunosurveillance by NK cells [106, 107]
**IL4R**	IL4 response is involved in promoting Th2 differentiation but the soluble form can inhibit IL4-mediated cell proliferation and IL5 upregulation by T-cells [108]
**IL7**	Forms a heterodimer with HGF. Important for proliferation during certain stages of B-cell maturation, T and NK cell survival [109]. Induces Th1 and Th17-associated cytokine secretion [110]. Elevated serum IL7 levels associates with COVID-19 [111]
**BTN3A2**	Plays a role in T-cell responses. Inhibits the release of IFNG from activated T-cells. sBTN3A may prevent T-cells from exerting their cytotoxic activity [112, 113]
**CXADR**	Role in tight junction integrity, transepithelial migration of leukocytes and neutrophils (interaction with JAML) [114, 115] and with JAML co-stimulation of epithelial γδ T cell activation [116]. Soluble form (sCAR) inhibits viral entry and inhibit other functions

**SPON2**	Innate immune response and a unique pattern-recognition molecule in the ECM [117]
**KLRD1**	Inhibitory receptor on NK-cells and memory/effector CD8-positive T cells. sKLRD1 has been reported in HIV patients [118]. May be reversing NK-cell suppression
**AOC1**	Digestive enzyme degrades compounds involved in allergic and immune responses, cell proliferation, tissue differentiation, tumor formation, and possibly apoptosis. Eosinophil and granulocytes increase diamine oxidase activity and release in acute inflammation [119-122]
**ST6GAL1**	Attachment of sialic acids to glycoproteins as a posttranslational modification influences cellular responses. Produced by platelets [123] and other cells and known as an acute phase reactant where blood level is upregulated during systemic inflammation [124]. Prolongs the activity of TNF, NFκB and STAT3 promoting the inflammatory phenotype of monocytic cells [125]. Enhances B cell IgG production and increases blood IgG titers [126]. Th2 polarization and M2 macrophages [124]
**BAG6**	BAG6 on exosomes a ligand of NK-cells receptor NCR3 and stimulates NK cells cytotoxicity but soluble ligand BAG6 suppressed NK-cells [127-129]
**LILRB1**	Binds to MHC I molecules on APCs and NK-/T-cells and inhibits immune and inflammatory responses. Soluble form is produced by splice variant and reverses the inhibition of NK cell cytotoxicity [56]
**LILRB2**	Binds to MHC I molecules on APCs and NK-/T-cells and inhibits immune and inflammatory responses. Soluble form is produced by a splice variant [56]. Soluble form (sLILRB2) blocks this immunosuppressive function and activates T-cells [130]
**MILR1**	Inhibitory role in the degranulation of mast cells. Also expressed on DCs, macrophages and neutrophils [131] - myeloid immunity. Serum MILR1 level associates with increased mast cells in circulation [132], may reverse the inhibitory role of membrane MILR1
**SIGLEC9**	Expressed on monocytes, neutrophils, B cells, NK cells, and minor subsets of T cells such as NK-cells as a vital inhibitory group. Engaging SIGLEC9 signaling suppresses neutrophil-mediated immunity, including inhibiting NETosis [133]. sSIGLEC9 inhibits its suppressive effect on neutrophils [134]. Soluble Siglec9 (sSIGLEC9) in the plasma can induce oxidative stress, and its expression can be increased by TNF-α, IL-6, and IL-8 [135]
**LILRA5**	Expressed on neutrophils, triggering innate immune responses, production of inflammatory signals such as IL6 and stimulate the early phases of immune responses [136, 137]
**GZMA**	T- and NK-cells specific serine protease for lysis of target cells. Reduced levels in COVID-19 severe patients associate with impaired NK- and cytotoxic T cell functions [138, 139]
**CD1C**	Antigen-presenting protein on DCs [140] presents to T-cell receptors on NKT-cells [141]. Soluble form would inhibit CD1C role in promoting Th1/Th17 function [140]
**KITLG**	Also called SCF. KITLG/SCF binding can activate several signaling pathways. Soluble form [142] secreted by fibroblasts and endothelial cells attracting mast cells.
**FLT3LG**	Stimulates the proliferation and differentiation of various blood cell progenitors [143]. sFLT3LG expands immature B-cells, NK-cells and DCs
**SSC4D**	Regulation of both innate and adaptive immune responses. Scavenger receptor [144]
**IGLC2**	Immunoglobulin Lambda Constant 2 (IGL@). Upregulated in plasma of critical (ICU) COVID-19 patients vs severe/mild [145, 146]

**CKAP4**	Anchoring of the endoplasmic reticulum to microtubules. Neutrophil degranulation [147]
**GRN**	Regulator of lysosomal function and as a growth factor involved in inflammation. Neutrophils produce progranulin and elastase capable of cleaving progranulin into granulin peptides promoting inflammation. High in cardiovascular disease [76]
**CXCL5**	Secreted by eosinophils and neutrophils [148] in response to inflammatory cytokines IL-1 or TNFα. Neutrophil activation [148, 149]
**IL15**	Stimulates phagocytosis of neutrophils [150, 151] and promotes T-cell proliferation during inflammation [152]
**VCAM1**	Expression by cytokine-activated endothelium. Neutrophil elastase and cathepsin G released by neutrophils cleaves VCAM1. Soluble VCAM1 (sVCAM1) is elevated in endothelial dysfunction and inflammation/fibrosis [153, 154]. Shed by platelets [91]
**CD63**	Receptor for TIMP1, leukocytes adhesion onto endothelial cells and a known marker for exosomes. Neutrophils/platelets degranulation [155, 156]
**MMP9**	Involved in local proteolysis of the ECM and in leukocyte migration. Roles in neutrophil-derived vascular endothelial damage and wound healing [157]. Associates with cardiovascular and respiratory failure in COVID-19 [158]
**MSR1**	Macrophage scavenger receptor glycoproteins (also called SR-A) implicated in the pathologic deposition of cholesterol in arterial walls during atherogenesis. Soluble form is high in arthritis inflammatory progression concomitant with increased Th17 response [159]. Neutrophil SRA expression is increased in sepsis and facilitates NETosis [160]
**TREM1**	Receptor involved in amplifying inflammatory responses. Soluble TREM1 is elevated during infection and shown to be a marker of sepsis and mortality [161]. Neutrophil stimulation/degranulation releases soluble TREM1 which can inhibit TREM1 receptor–mediated proinflammatory cytokine production [162]
**AZU1**	Expressed in specialized lysosomes of the neutrophils. Mediating recruitment of monocytes in the second wave of inflammation. Released in neutrophil NETs [104]
**CEACAM8**	Activated neutrophils, neutrophil degranulation. Released in neutrophil NETs [104], associates with acute-phase response, inflammation and immune response [163]
**MPO**	Major component of neutrophil azurophilic granules and mediates activation. Elevated in Severe COVID-19 [84]. Also cleaved from vascular endothelium by proteases [164]
**PRTN3**	Serine protease degrades ECM. Released in neutrophil NETs [104]
**S100A12**	Also called EN-RAGE and plays a prominent role in the regulation of inflammatory processes and immune response. Implicated in COVID-19 severity [165]
**SIRPA**	Released in neutrophil NETs [104]. SIRPα on macrophages interaction with CD47 on RBCs prevents phagocytosis [166]; soluble form might block the phagocytosis inhibition
**CTSC**	Cathepsin C. Activation of various pro-inflammatory serine proteases from neutrophils and mast cells [167]. Neutrophilic lung inflammation [168]
**CHI3L1**	Th2 inflammatory response and IL13-induced inflammation, DCs accumulation and M2 macrophage differentiation [169, 170]. Released by neutrophils [170-172]

**CSF3**	G-CSF induces granulocytes and neutrophils release [173]
**DEFA1**	A definsin abundant in the granules of neutrophils and other cells [174]. Elevated in plasma during infection and inflammation [174, 175]
**LCN2**	Innate immunity [176] and elevated in severe COVID-19 [177]
**NID1**	Basement membrane glycoprotein, role in cell interactions with ECM. Promotes neutrophil adhesion and has a potent chemotactic activity for neutrophils [178]. Cleaved from vascular endothelium by proteases [164].
**TIMP1**	Metalloproteinase inhibitor. Growth factor/integrin signaling via CD63 and ITGB1. Neutrophilia via CD63 [179]. High in cardiovascular disease [180]. Cleaved from vascular endothelium by proteases [164]
**F3**	Tissue factor (TF) function in blood (extrinsic) coagulation initiation forming a complex with circulating factor VII or VIIa. Blood TF contributes to thrombosis [181] and associates with sepsis [182] and acute myocardial infarction [183, 184]
**PROC**	Protein C regulates blood coagulation by inactivating factors Va and VIIIa and cellular anti-inflammatory signaling (THBD/EPCR/PAR-1[cleaved by PROC instead of thrombin]) [185]. Circulating form is pro-enzyme/zymogen (not activated)
**NOS3**	eNOS plays crucial roles in regulating vascular tone, leukocyte adhesion, platelet aggregation, and anti-inflammatory role. eNOS was reported in plasma and serum [186, 187]; endothelial cell death may release eNOS during sepsis and oxidative stress, suggesting eNOS uncoupling with proinflammatory and coagulopathy consequences [188, 189]
**PLAT**	tPA generates plasmin, involves in tissue remodeling and degradation. High serum levels in acute myocardial infarction [190, 191]
**TFPI**	Antithrombotic against factor x from the extrinsic coagulation pathway. Increased in critically ill COVID-19 patients [192]
**MCFD2**	Plays a role in the secretion of coagulation factors (factors V and VIII) [193]. MCFD2 is secreted via a classical secretion pathway [194]
**MMP10**	Thrombin induces endothelial MMP10 levels through a PAR1 (F2R)-dependent mechanism. MMP10 serum levels associate with inflammatory markers and arterial diseases [195-197]
**MMP3**	Activated by plasmin and activates MMP7. MMP3 serum levels are associated with inflammatory markers and COVID-19 severity [198]
**FAM3C**	Promotes EMT, relevant to inflammation/fibrosis. Released by platelets [199]
**MMP7**	Activated by plasmin. Serum level associate with lung fibrosis, COVID-19 severity, and other diseases [177, 200, 201]
**CR2**	Also called CD21 is receptor for complement C3 and binds to CD19 on B-cells. Soluble CD21 in the blood is mainly derived from follicular DCs [202] and it competes for C3 binding and inhibits CD21-CD19 B-cell activating function [203]
**F2R**	Also called PAR1, a receptor for activated thrombin expressed in platelets and endothelial cells mediating the interplay between coagulation and inflammation [204]. Shedding makes PAR1 unresponsive to thrombin [205]

**PLAU**	Converts plasminogen to plasmin and leads to D-dimers [206], function as stimuli for inflammatory cell (neutrophils, monocytes, macrophages) production of cytokines. TNFα or IL-1 induce the expression of uPA from endothelial cells [207, 208]
**THBD**	Thrombomodulin is a cofactor in the thrombin-induced activation of protein C (PROC). Soluble thrombomodulin associates with mortality, hospital stay and ICU in COVID-19 patients [209]. Cleaved to its soluble form by neutrophil elastase during acute and chronic inflammatory responses immunologic reactions and complement activation [210]
**C2**	Part of the classical pathway of the complement
**CFHR5**	The dimerized forms have avidity for tissue-bound complement fragments and efficiently compete with the physiological complement inhibitor CFH [211]
**F11**	Blood (intrinsic) coagulation by activating factor IX
**MMP12**	Degrades soluble and insoluble elastin. Role in countering neutrophil infiltration, clearing NETs, and dampening inflammatory pathways [212]
**PLAUR**	High level of suPAR associates with COVID-19 severity [213], suPAR levels are positively correlated with high-sensitivity C-reaction protein (hs-CRP), neutrophil/leukocyte ratio, and lymphocyte counts [214]. Cardiovascular disease [13]
**TIMP4**	Protease inhibitor, inhibits platelet aggregation and recruitment [215]
**VWF**	Promotes adhesion of platelets to the sites of vascular injury [216]
**PTX3**	Acute phase response protein, activates the classical pathway of complement activation and facilitates pathogen recognition by macrophages and DCs [217-220]
**VCAN**	Involved in inflammation-related interactions with leukocytes and chemokines to recruiting inflammatory cells, particularly in inflammatory lung conditions. Cleaved by MMPs and plasmin and presence in circulation promotes coagulation [221]. Cleaved from vascular endothelium by proteases [164]
**SMOC1**	Promoting endothelial cell proliferation and angiogenesis [222] and coagulation [223]
**LTA**	Homotrimeric form binds to TNFRSF1A/TNFRSF1B/TNFRSF14. Heterotrimeric form with LTB binds to TNFRSF3/LTBR. Lymphotoxin is produced by lymphocytes as a cytotoxic. Increased in cardiovascular disease [180]
**LTBR**	TNFRSF3 is receptor for LTA/LTB and TNFS14/LIGHT, promotes apoptosis and role in lymphoid system. High levels of circulating LTβR associated with cardiovascular risk factors, multiple inflammatory markers, and markers of cardiac injury [224]
**TNFRSF1A**	Receptor for TNFSF2/TNFa and homotrimeric TNFSF1/LTA. Contributes to the induction of non-cytocidal TNF effects including anti-viral state. Shed off neutrophils and T-cells [225-227]. Soluble sTNFR1 associates with nephropathy, cardiovascular events, heart failure [228-230]
**TNFRSF1B**	Receptor for TNFSF2/TNFa and homotrimeric TNFSF1/LTA, mediates most of the metabolic effects of TNFa. It is shed of neutrophils and T-cells [225-227]. Soluble form (sTNFR2) associates with nephropathy, cardiovascular events, heart failure [228-230]
**TNFRSF14**	Receptor for TNFSF14/LIGHT and homotrimeric LTA. Has different cis/trans signaling with activating/inhibitory effect on immunity. Soluble form (sHVEM), by cleavage, is increased during inflammation and inhibits LIGHT function. Increased in severe COVID-19 [231]

**TNFSF11**	RANKL binds to TNFRSF11B and TNFRSF11A. Augments the ability of dendritic cells to stimulate naive T-cell proliferation. sRANKL decreased in coronary artery disease [232, 233]
**TNFRSF11B**	OPG is a decoy receptor for TNFSF11/RANKL and thereby neutralizes its function [234]; it is upregulated in coronary heart disease [232]
**TNFRSF11A**	TNFRSF11A/RANK is a receptor for TNFSF11. Involved in the regulation of interactions between T-cells and dendritic cells [235]
**CD40**	Receptor for TNFSF5/CD40LG. Mediates a broad variety of immune and inflammatory responses. Soluble CD40 has immunosuppressive effects, reduced T-Cells and INFγ secretion, and is elevated in atherosclerotic vascular disease [236]
**TNFSF10**	TRAIL binds to TNFRSF10A/TRAIL-R1, TNFRSF11B/OPG and others. Binding to the decoy receptor OPG cannot induce apoptosis. sTRAIL correlates with inflammatory cytokines and CD68 expression and plaque cell apoptosis, plaque inflammatory activity, and with symptomatic carotid plaques [237]
**TNFRSF10A**	TRAIL-R1. Promotes the activation of NF-kappa-B. Essential for ER stress-induced apoptosis. Soluble TRAIL-R1 (sTRAIL-R1) has been reported in cancer [238, 239] and ankylosing spondylitis [240]
**TNFRSF10B**	TRAIL-R2 promotes the activation of NF-kappa-B. Essential for ER stress-induced apoptosis. Higher plasma levels of sTRAIL-R2 had a higher risk of future cardiovascular events [13]. sTRAIL-R2 correlates with inflammatory cytokines and CD68 expression and plaque cell apoptosis, plaque inflammatory activity, and with symptomatic carotid plaques [237]
**TNFSF12**	TWEAK. Promotes angiogenesis and the proliferation of endothelial cells [241]. Induction of inflammatory cytokines [242]. Promotes IL8 secretion [243]
**TNFRSF12A**	Receptor for TNFSF12/TWEAK. Promotes angiogenesis and the proliferation of endothelial cells. Soluble form (sFn14) has been described acute and chronic kidney diseases [244]
**TNFRSF13B**	TACI is a receptor that stimulates B- and T-cell function and the regulation of humoral immunity T-independent humoral response [245, 246]. Soluble form acts as a decoy receptor inhibiting ligand-mediated B-cell survival/function and NFκB-activation [247]
**TNFRSF9**	Soluble forms (sCD137) released by activated T cells [248] is antagonistic and reduces immune activity [249, 250]. High in cardiovascular disease [180, 251]
**EDA2R**	Mediates the activation of the NF-kappa-B and JNK pathways. Shed form reported [252] and protects from apoptosis. High in cardiovascular disease [180]
**FAS**	Receptor for TNFSF6/FASLG. The secreted isoforms 2 to 6 (sCD95) block apoptosis [253], elevated in liver disease [254], kidney injury [255], angina [256]
**CXCL16**	Scavenger receptor on macrophages, which specifically binds to OxLDL (oxidized low-density lipoprotein). A secreted splice variant by DCs is a chemoattractant for CXCR6+ cells [257]
**SLITRK2**	Released in neutrophil NETs [104]. Expressed predominantly in neural tissues and have neurite-modulating activity. Suppresses neurite outgrowth (Axonogenesis) and may be involved in leukocyte chemotaxis [258]
**CCL11**	Promotes the accumulation of eosinophils, but not mononuclear cells or neutrophils, a prominent feature of allergic inflammatory reactions [259]

**CCL15**	Attracts T-cells and monocytes, but not neutrophils, eosinophils, or B-cells (UniProtKB)
**CCL23**	Attracts monocytes, resting T-Cells, and neutrophils, not activated lymphocytes (UniProtKB)
**CCL3**	Attracts inflammatory cells; macrophages, monocytes and neutrophils [260]
**CCL7**	Attractant for monocytes and eosinophils, not neutrophils (UniProtKB)
**CX3CL1**	Soluble form is chemotactic for T-cells and monocytes and not for neutrophils [261]
**CXCL11**	Attractant for interleukin-activated T-cells but not unstimulated T-cells, neutrophils, or monocytes (UniProtKB)
**CXCL9**	Attractant for activated T-cells (UniProtKB)
**CLEC5A**	Activation of CLEC5A on neutrophils and macrophages induce neutrophil extracellular trap (NET) formation and proinflammatory cytokine release [262, 263]
**CLEC4D**	Expressed in resting macrophages [262]. CLEC4D modulates T-cells toward effector T-helper 1 and T-helper 17 cell subtypes [264]
**CLEC7A**	Expressed in DCs, neutrophils and other immune cells and engages signaling cascades that drive innate and adaptive immunity, inflammatory cytokine secretion and DC maturation to prime CTL CD8+ and Th1/Th17 cells [262]
**CLEC1A**	Expressed on APCs, myeloid cells, and ECs and reduces Th17 differentiation and increases Tregs [265-267]. Soluble form inhibited the HRG-induced neutrophil rounding, phagocytic activity, and prolongation of survival time [268]
**CLEC6A**	Expressed in macrophages, monocytes, neutrophils and several DC subtypes and activation leads to cytokines release and induce a mixed Th2/Th17 response [262]
**CLEC4A**	Expressed on monocytes, macrophages, granulocytes, B cells, and DCs cross-presentation to CD8+ T cells [262]
**CLEC10A**	Expressed in subsets of DCs and macrophages and is used as a marker of alternative macrophage activation and plays an anti-inflammatory role [262]
**CLEC4C**	Antigen capturing by DCs; its reduction is a marker of DCs maturation [262, 269]
**CD5**	Regulation of T-cells and B-cells [270]. Soluble form of CD5 associate with autoimmune disease and inflammation/sepsis patients [271], sCD5 as decoy receptor for the treatment of inflammation/sepsis [270]
**CXCL10**	CXCL10 is secreted by several cell types in response to IFN-γ. These cell types include monocytes, endothelial cells and fibroblasts [272] chemoattraction for monocytes, macrophages, T cells, NK cells, and DCs, promotes T cell adhesion to endothelial cells [273]
**RETN**	Adipokine associated in several pathologies including cardiovascular disease [274], and pro-inflammatory effect [275] with levels are correlated with inflammatory and fibrinolytic markers such as CRP, TNF-α, and IL-6 [274]
**AGR2**	Secreted in mucus including the lungs and modulates cell migration/adhesion, cell differentiation and cell growth. Pro-inflammatory [276]

**IL5RA**	Subunit of the Interleukin-5 receptor. Soluble IL5RA (sIL-5Rα) is encoded [277] and increases with the eosinophil count [278], functional impairments in B cells and eosinophils [279] and would inhibit the protective effect of IL-5 during sepsis [280]
**DKK1**	Antagonist of the Wnt/β-catenin signaling pathway. Increase in blood is associated with inflammation and infection [281]
**TDGF1**	Also called Cripto-1, exists as cell-associated and secreted (shedding by TMEM8A) form signaling in cis and trans. Cripto-1 enhances macrophage phagocytic activity and upregulates the production of pro-inflammatory cytokines [282], also enhances pro-inflammatory TNFα from CD4+ T helper cells [283]
**SMPD1**	Secreted form [284] is increased in response to stress and inflammatory mediators (IL1B and TNF) and viral infection [285] and coagulation during SARS-CoV-2 [286]. Converts sphingomyelin to ceramide which facilitates SARS-CoV-2 infection [287, 288]
**NUCB2**	Release of tumor necrosis factor from vascular endothelial cells [289]; regulate inflammatory responses [290]. NUCB2/nesfatin-1 correlated positively with plasma levels of IL6, and TNFα, IL8 in chronic obstructive pulmonary disease [291]
**TCN2**	Vitamin B12-binding and transport protein, high serum levels in inflammation/infection and liver disease, and characteristics of acute-phase reactant [292, 293]
**NPM1**	Several cellular processes. NPM1 can be passively released by necrotic or damaged cells, or secreted by endothelial cells, monocytes, and macrophages under stress/infection. Released in neutrophil NETs [104]. Extracellular NPM1 acts as a potent inflammatory stimulator promoting cytokine production. NPM described as an alarmin [294]
**CSF1**	M-CSF. Release of proinflammatory chemokines, role in innate immunity and inflammatory processes, influencing function of macrophages [295]
**CD163**	Acute phase, inflammatory response. Released from M2 macrophages during chronic inflammation/sepsis related to TNFα and TACE/ADAM17 activity [296], and cardiovascular disease [76]. A valuable diagnostic parameter for monitoring macrophage activation in inflammatory conditions including COVID-19 [297]
**REG4**	Involved in inflammatory and metaplastic responses of the gastrointestinal epithelium [298], polarization macrophages to M2 phenotype [299]
**ANPEP**	Also called CD13, expressed on small-intestinal and renal microvillar membrane. Soluble form (sCD13) is a pro-inflammatory mediator [300] and shown in severe COVID-19 [301]
**CRH**	Anti-inflammatory peptide released during stress and leads to cortisol production, an anti-inflammatory hormone [302]
**STK4**	Stress-activated, pro-apoptotic kinase. Serum STK4 levels are reduced with increased IL6 and increased inflammation [303]
**LRP1**	Intracellular signaling and endocytosis implicated in many biological processes. Shed by ADAM proteases. sLRP1 a biomarker of the level of atherosclerotic plaques and coronary artery events [304]. sLRP1 is generated in inflammation and may regulate inflammation by its effects on macrophage secretion of TNF-α, MCP-1/CCL2, and IL-10 [305]
**LGALS8**	Sensor of membrane damage caused by infection and restricts the proliferation of infecting pathogens by targeting them for autophagy. Gal-8 plays a role in innate and adaptive immunity and inflammation [306], pro-inflammatory activities in the endothelium [306]

**LGALS4**	Sensor of membrane damage, pro-inflammation inducing CD4+ T cells to produce IL-6 [307]
**KLK10**	Proteas. Represses proliferation. Inhibits endothelial Inflammation and atherosclerosis [308]
**ADM**	Blood pressure, hypotensive effect in blood vessels. Activates eNOS (NOS3) for NO production [309]. Strongly elevated in patients with sepsis, and in patients with hypertension and acute heart failure where high levels could reflect residual tissue congestion [310, 311]. Augments the release and production of TFPI [312] and predicts COVID-19 mortality [313]
**TNC**	Endothelial/inflammatory cardiomyopathy. Upregulated in blood of sepsis patients [314, 315]. Cleaved from vascular endothelium by proteases [164]
**SFRP1**	sFRP1 is an antagonist of Wnt signaling and is elevated in cardiovascular disease [316, 317]
**CALCA**	Vasodilator with high serum level associating with CAD [318, 319]
**ACE2**	Soluble form converts angiotensin I into the vasodilator angiotensin 1-7 [320] and associate with CVD development [321] and COVID-19 severity [322]
**IGFBP2**	Inhibits IGF-mediated growth. High levels associate with severity of pulmonary arterial hypertension [323]. Predictor of mortality in chronic and acute heart failure patients [324]
**MME**	Mature neutrophils marker, soluble form (Neprilysin or CD10) predicts heart failure [325]
**NPPB**	Heart failure, relates to MME inhibitors [326, 327]
**FRZB**	Also called sFRP3 is an antagonist of Wnt signaling and may augmenting myocardial injury-driven fibrosis [328], Cleaved from vascular endothelium by proteases [328]
**NT-proBNP**	Heart failure [326, 327]
**OSM**	Oncostatin M has a pro-inflammatory effect on cytokine production by endothelial cells, including IL-6, G-CSF and GM-CSF. Induces dedifferentiation of cardiomyocytes, promotes progression of heart failure [329]
**LDLR**	sLDLR reduces uptake of triglycerides and contributes to atherosclerosis [330]
**CDH2**	Cell adhesion protein and the soluble form inhibits cell-cell adhesion, inhibits vascular smooth muscle cell (VSMC) and macrophage apoptosis which contributes to myocardial infarction [331-333]. Released in neutrophil NETs [104]
**PDGFRA**	Plays a role in platelet activation, secretion of agonists from platelet granules, and in thrombin-induced platelet aggregation. Soluble form has been described [334] and might inhibit the function of membrane form leading to anti-proliferative effect on vascular endothelial cells
**REN**	Generates angiotensin I from angiotensinogen in the plasma, vasoconstriction, and increase in blood pressure [335]
**AGER**	Mediator of acute and chronic vascular inflammation. sAGER (sRAGE) is unclear for role in CVD [336]
**ACP5**	Serum ACP5 (TRAP/TRAP5b) higher in coronary artery disease patients and loss of bone mineral density. High in cardiovascular disease [337]
**TNNI3**	Cardiac Troponin I (cTnI) is exclusively expressed in adult cardiac muscle. High blood level is an indicator for myocardial ischemia and infarction [180, 338, 339]

**IGFBP1**	Promotes cell migration. High levels associate with cardiovascular disease [340-342]
**ANGPTL1**	A key anti-angiogenic protein (it is also known as angioarrestin) by inhibiting the proliferation, migration, tube formation, and adhesion of endothelial cells [343]
**C1QTNF1**	Serum levels are high in CAD and associate with CAD severity and TNFα and IL6 [344]
**DCN**	Ligand for multiple cell surface receptors mediates its role in tumor suppression, including a stimulatory effect on autophagy and inflammation and an inhibitory effect on angiogenesis. Cleaved from vascular endothelium by proteases [164]
**SORT1**	A sorting receptor in the Golgi compartment and as a clearance receptor on the cell surface. Soluble sortilin in serum/plasma associate with atherosclerosis, coronary artery disease, and peripheral arterial disease [345-347]
**PCSK9**	Pro-atherosclerotic effects leading to elevated levels of LDL, low HDL levels, obesity and overweight, diabetes, and coronary heart disease [348, 349]
**PGF**	Growth factor active in angiogenesis and endothelial cell growth. Found within human atherosclerotic lesions is associated with plaque inflammation [350]
**SPARCL1**	Actively released from quiescent endothelial cells via the classical secretion pathway and inhibits angiogenesis, endothelial cell proliferation and migration but required for capillary morphogenesis and integrity [351, 352]
**ANGPT2**	Antagonist for both ANGPT1 and TIE2, disrupts the vascular remodeling ability of ANGPT1 and may induce endothelial cell apoptosis. High in cardiovascular disease [180, 337]
**COL4A1**	Cleaved into arresten, comprising the C-terminal NC1 domain that inhibits endothelial cell proliferation, migration, and tube formation [353-355]. Cleaved from vascular endothelium by proteases [164]
**TGFBI**	Inhibit cell adhesion. Plasma TGFBI remains high in recovered COVID-19 patients [356]. Induced in various forms of heart disease affecting fibrosis and disease responsiveness [357] Cleaved from vascular endothelium by proteases [164]
**PRELP**	Present in connective tissue ECM. Elevated serum level in pulmonary hypertension [358] and elevated in cardiac ECM after myocardial ischemia/reperfusion injury [359]. Cleaved from vascular endothelium by proteases [164]
**SPON1**	Cell adhesion. Serum/plasma SPON1 significantly higher in cardiovascular disease/heart failure [13, 76, 180, 337]
**IGFBP7**	Binds to IGF with high affinity and stimulates cell adhesion. Roles in cardiac hypertrophy, fibrosis, cellular senescence, insulin resistance, endothelial dysfunction, and inflammation. Increase IGFBP7 reflects worsening diastolic function, adverse cardiac remodeling, metabolic derangement, and heart failure [360]. Cleaved from vascular endothelium by proteases [164]
**SOD2**	Mitochondrial matrix protein that clears mitochondrial reactive oxygen species, protective against apoptosis. The concentrations of plasma SOD1 and SOD2 were higher in CAD than in healthy controls [361]
**NRP2**	Involved in cardiovascular development, axon guidance, tumorigenesis, inflammation, and cardiovascular disease. Soluble form (sNPR2) [362] acts a decoy inhibiting function [363]
**BMP4**	Regulates development including heart development and adipogenesis. Role in cardiomyocyte induction [364]

**GDF2**	GDF2 (BMP9) binds to ACVRL1 and is potent circulating inhibitor of angiogenesis inhibiting microvascular endothelial cell migration and growth [365]
**IGFBP3**	Main IGF transport protein in the bloodstream. Serum levels significantly reduce during the catabolic flow phase of injury [366], acute myocardial infarction and coronary heart disease [367]
**COMP**	COMP is a marker of cartilage turnover, role in vascular wall remodeling [368, 369]. Cleaved from vascular endothelium by proteases [13]
**PON3**	Associates with HDL and inhibit the oxidation of LDL to slow the initiation and progression of atherosclerosis [370]
**DKK4**	Antagonist of the Wnt/β-catenin signaling pathway. Increase in blood is associated with cardiovascular disease [371]
**FKBP5**	FKBP5–NFκB signaling mediates inflammation, potentially contributing to cardiovascular risk [372]. Plasma level is increased in stroke patients [373]
**PRSS2**	Increased serum/urine levels in acute and chronic pancreatitis [374]
**FBP1**	Metabolism, gluconeogenesis. High serum level in acute liver failure [375]
**FST**	Bioneutralization of members of the TGF-β superfamily, antagonist of activin (multifunctional protein including immune response and wound repair). High Follistatin (FST) associates with COVID-19 severity and mortality reflecting local (lung and endothelium) and system damage and inflammation [376]. High in cardiovascular disease [13]
**SFTPD**	Pulmonary surfactant-associated protein D involved in lung's defense against inhaled microorganisms, organic antigens and toxins [377]. Leakage from the lung into circulation is a promising biomarker for lung injury [377].
**LRIG1**	Interact with RTKs (the EGFR family, MET and RET) as a feedback negative regulator of signaling by RTKs with role in homeostasis. Soluble form (sLRIG1) retains this inhibitor function in a paracrine manner [378]
**GDF15**	Inflammation, tissue hypoxia, acute injury and oxidative stress, and cardiovascular disease [13], induced in lung injury [379]
**LRPAP1**	Chaperon for LRP1, leakage in circulation due to tissue damage [380]. RAP Inhibits ligand binding to LDLR.
**PTN**	Endothelial cell migration and neovasculogenic effects in damaged heart, cardiomyocyte programmed cell death in response to pro-apoptotic stress, which may be critical to myocardial injury repair [381-383]
**RSPO1**	Ligand for LGR4-6 receptors activating canonical Wnt signaling by relieving the Dkk1 inhibition imposed on the Wnt pathway. Excess levels are linked to liver fibrosis [384, 385]
**KRT19**	KRT19 fragment (CYFRA21-1) relates to lung pathologies. Found high in severe/critical/deceased COVID-19 [386]
**MATN3**	Cartilage specific, role in the formation of extracellular filamentous networks. Presence in circulation my reflect tissue damage [387].
**MB**	Binds oxygen on a heme group. Released into the bloodstream after muscle injury [388], potential marker for heart attack [389]

**HGF**	Growth factor for a broad spectrum of tissues and cell types and functions. Neutrophils activation [390], liver disease [391], hypertension [392], cardiovascular [393], advanced heart failure [394], viral load and lung injury, severity and mortality in COVID-19 patients [395, 396], and immune suppression, reduced antigen presentation [397] and other suppressive effect on cytotoxic cell killing [398]
**PROK1**	Potently contracts gastrointestinal smooth muscle. Role in cardiovascular health and disease, marker for heart and kidney damage [399]
**REG1A**	Sepsis [400-402]
**TINAGL1**	Kidney inflammation/damage and cardiovascular damage. Cleaved from vascular endothelium by proteases [164]
**GFRA1**	Axon guidance/development. Co-receptor with RET for GDNF to mediate RET tyrosine kinase signaling. Released form described from neural cells and nerve injury which mediates trans signaling [403, 404]
**CPA1**	Acute and chronic pancreatitis [405, 406]
**NEFL**	Presence in plasma reflects axonal/neuronal damage [407], and stroke [408, 409]
**PVALB**	Muscle relaxation. Serum parvalbumin during muscle injury [410] and neural damage [411]
**CPB1**	Pancreatitis [412]
**CTSL**	Cathepsin. High serum level associates with COVID-19 severity, may play a role in SARS-CoV-2 entry; contributes to fibrosis in COVID-19 [413]. Marker of cardiovascular event [414]
**CSTB**	Cathepsin. Released in neutrophil NETs [104]. Reversible inhibitor of cathepsins L, H and B. Blood CSTB (Cystatin B) is a marker of cardiovascular event [414, 415]
**CTSZ**	Cathepsin. Cathepsin. Might contribute to fibrosis in COVID-19 [413]. Cleaved from vascular endothelium by proteases [164]
**FCAR**	Several functions including cytokine, proinflammatory, production. Neutrophil activation and immunity [416]. CD89 (FCAR) serves as an innate receptor during the early phase of infection [417]. Soluble form (sCD89) a biomarker for IgA nephropathy [418]
**CTSD**	Cathepsin. Might contribute to fibrosis in the lung, spleen, thyroid, liver, and heart in COVID-19 [413]. Cleaved from vascular endothelium by proteases [164]. Marker of cardiovascular event [414, 415]
**CTSO**	Cathepsin. Might contribute to fibrosis in COVID-19 [413]
**CTSH**	Cathepsin. Might contribute to fibrosis in COVID-19 [413]
**LYVE1**	Binds to hyaluronic acid (HA), cell surface receptor on lymphatic endothelial cells. Macrophage-derived LYVE-1 is shed by metalloproteinases [419] which might induce arterial stiffness and collagen deposition [420]. Shedding inhibits LYVE-1-mediated lymphangiogenic responses [421] and may promote pathological lymphangiogenesis [422]. High serum LYVE-1 during acute lower respiratory Infection and renal dysfunction [423]
**EPHB4**	Role in heart morphogenesis, angiogenesis and blood vessel remodeling and permeability. sEphB4 blocks activation of EphB4 and EphrinB2; suppresses endothelial cell migration, adhesion, and tube formation [424]

**ROBO2**	SLIT2/ROBO2 guidance cue in cellular migration, including axonal navigation and angiogenesis. Reduced sROBO2 (shedding by ADAM10 at the ectodomain) suggests low activity of the SLIT2/ROBO2 signaling [425, 426]
**PLXDC1**	Role in endothelial cell capillary morphogenesis [427]. Secreted form from transcript variants may act as decoy thus inhibiting capillary repair
**ASGR1**	Mediates the endocytosis of plasma glycoproteins; soluble form (sASGR1) [428] is upregulated in liver fibrosis/cirrhosis [429]
**HSPB1**	HSPB1 (HSP27) is released from platelets during activation/aggregation, and serum levels of HSP27 associate with inflammation and other tissue injuries [430, 431]
**IFI30**	Expressed in antigen-presenting cells and induced by INFγ in other cell types. Important in MHC class II-restricted antigen processing and restricts viral entry including SARS-CoV [432]. Secreted IFI30 (GILT) may enhance hemolysin-mediated tissue damage [433]
**HMOX1**	Heme catabolism. High serum/plasma levels in ARDS and interstitial lung disease [434] and acute kidney injury [435]
**HMOX2**	Heme catabolism. High serum/plasma levels in ARDS, interstitial lung disease, peripheral artery disease, acute kidney injury [436]
**MERTK**	RTK which binds to several ligands including GAS6. sMERTK act as a competitive inhibitor of MerTK by acting as a decoy for its ligand GAS6 inhibiting the anti-inflammatory function of GAS9/TAM signaling in macrophages [437, 438]. sMERTK shed during inflammatory responses and inflammatory cardiovascular lesions [437, 438], and associates with kidney disease and sepsis [439, 440]
**CST3**	Biomarker of kidney function [441, 442] and predicting new-onset or deteriorating cardiovascular disease [443]. sCST3 is a predictor of COVID-19 severity [444, 445]
**TYMP**	Role in maintaining the integrity of the blood vessels, promoting activity on endothelial cells, angiogenic activity. High serum level in sepsis [446], and associates with COVID-19 associated thrombotic event, inflammation, and organ damage [447]
**SNCG**	γ-Synuclein is found primarily in the peripheral nervous system. Serum/urine presence detected in cancer & secreted by cancer cells [448]. May mark peripheral nerve damage [449]
**TFF2**	Inhibits gastrointestinal motility and gastric acid secretion repair repairing the gastrointestinal tract [450]. Increased in serum with kidney disease [451] and declining lung function [452]
**CCDC80**	Cell adhesion and matrix assembly. Blood levels are linked to glucose tolerance derangements and related to inflammation-associated chronic complications in diabetes [453], and metabolic and cardiovascular risk in patients with inflammatory bowel disease [454]
**CA3**	Muscle specific CA released after muscle injury. Serum myoglobin/carbonic anhydrase III ratio as a marker of reperfusion after myocardial infarction [455]
**SPOCK1**	Cell-cell and cell-matrix interactions. Soluble form (Testican-1) is upregulated in sepsis and associates with sepsis severity [456]
**TFF3**	Protects the mucosa from insults, stabilizes the mucus layer and affect healing of the epithelium, repair of the intestinal mucosa and lung. Marker for lung inflammation/cancer and declining lung function [452, 457]

**EZR**	Linker between plasma membrane and actin cytoskeleton. Ezrin in blood could mark tissue damage, particularly the lung [458]
**TACSTD2**	Cell surface receptor that transduces calcium signals for self-renewal, proliferation, invasion, and survival; stem cell-like qualities. Cleaved to release the extracellular domain and the intracellular domain translocases to the nucleus; both fragments increase hyperplasia [459]. Relevant to bronchial cells/lung repair including proinflammatory secretion and hyperplasia
**TGFA**	Wound healing and tissue repair. TGFα has a broad mitogenic effect including epithelial development. Mediates injury-induced lung fibrosis [460]
**THBS2**	Adhesive glycoprotein that mediates cell-to-cell and cell-to-matrix interactions, a ligand for CD36 mediating antiangiogenic properties [461]. High serum levels associate with fibrosis and cardiovascular diseases, heart failure and aortic aneurysm [462-467]
**IGSF3**	In lungs, it increased cell adhesion and decreased cell migration thus may be involved lung tissue repair [468]
**AXL**	Several roles in host-virus interaction and immunity. sAXL is high in severe sepsis, sepsis, and infection [440, 469]
**GAS6**	Ligand for AXL, secreted by endothelial cells and is important for the activation of endothelium during inflammation. High in severe sepsis, sepsis, and infection [469]
**ICAM2**	Interacts with EZR. Mediates adhesive interactions important for antigen-specific immune response, NK-cell mediated clearance, lymphocyte recirculation. Soluble ICAM2 (sICAM2) is elevated in endothelial dysfunction and inflammation/fibrosis [470]. Shed by platelets [91]
**ICAM1**	Plasma levels are predictive of COVID-19 mortality and organ failure [471]. Role in leukocyte trans-endothelial migration [472]
**SOST**	Negative regulator of bone growth that acts through inhibition of Wnt signaling and bone formation. Serum Sclerostin associated with ICU disease severity independent of the presence of sepsis and correlated with biomarkers reflecting renal, hepatic, and cardiac dysfunction, and biomarkers reflecting bone metabolism [473]
**ITGB6**	Expressed on epithelial cells, including lung. Plays key role in TGF-beta-1 activation and inflammation and lung fibrosis [474]
**CDH1**	Cell adhesion protein. Shedding of cleaved E-cadherin molecules during inflammatory response [475]. Blood soluble E-cadherin might reflect tissue injury in the events of inflammation [476]. Shed by platelets [91]
**CD59**	Potent inhibitor of the complement membrane attack complex. Soluble form has greatly reduced ability to inhibit MAC assembly on cell membranes [477]. sCD59 biomarker for glucose handling and diabetes [478], associated with cellular damage after acute myocardial infarction [479], and lung dysfunction after lung transplant [480]
**MET**	Wound healing, organ regeneration and tissue remodeling. Soluble c-Met relates to liver injury [481], diabetic nephropathy [482]
**CD93**	Cell-cell adhesion and host defense. Expressed on many cell types including platelets, neutrophils, monocytes, microglia, and endothelial cells. Soluble CD93 (sCD93) associates with CAD, acute myocardial infarction [483] inflammation [484]
**DLK1**	Soluble form cleaved off by ADAM17 is active in inhibiting adipogenesis neuroendocrine differentiation, reduced level associate with myocardial fibrosis [485]

**FGF19**	Reduced blood level in certain metabolic disorders [486], non-alcoholic fatty liver disease [487] and insulin resistance [488]. FGF19 have anti-fibrotic properties in the lung [489]
**TSHB**	Indispensable for the control of thyroid structure and metabolism (UniProtKB)
**MSTN**	Negative regulator of skeletal muscle growth. Low serum level post-myocardial infarction associate with improved survival, possibly by limiting extent of fibrosis [490]
**ENPP7**	Sphingomyelinase that hydrolyses sphingomyelin to ceramide in the intestinal tract. It has features to be secreted in bile and was detected in medium [491], released in intestinal lumen by bile salts and enzymes [491, 492]
**SULT2A1**	Enzyme in maintaining steroid and lipid homeostasis. Serum level was shown to be a marker of liver injury mouse model [493]
**PTS**	Metabolism; involved in serotonin biosynthesis and NO synthase activity. Induced by IL1B and INFg in endothelial cells [494]. Possibly released due to apoptosis/cell death from tissue damage.
**HAO1**	Oxidative stress. Located in the peroxisome and expressed in liver and pancreas [495]. Possibly released due to apoptosis/cell death from tissue damage.
**MAD1L1**	Mitotic checkpoint, spindle-assembly checkpoint (UniProtKB). Possibly released due to apoptosis/cell death from tissue damage.
**NADK**	Redox. Located in Nucleoplasm, Vesicles (the Human Protein Atlas). Possibly released due to apoptosis/cell death from tissue damage.
**ANXA10**	Undetermined function
**CA5A**	Ureagenesis and gluconeogenesis. Mitochondrial enzyme in the liver, kidney, and skeletal muscle (the Human Protein Atlas)
**HEXIM1**	RNA polymerase II transcription inhibitor. Regulation of innate immune response (the Human Protein Atlas)
**ZBTB17**	Transcription factor, prevents apoptosis in lymphoid precursors, allowing them to survive in response to IL7 and undergo proper lineage commitment (the Human Protein Atlas)
**LAMP3**	Also called CD208 and DC-LAMP, and almost exclusively found in matureDCs with role in dendritic cell function and in adaptive immunity, DCs maturation (the Human Protein Atlas)
**CES1**	Manage cellular cholesterol esterification levels and expressed in monocytes (called monocyte esterase). May be secreted in other species but no evidence in humans apart from liver cancer [496]
**PRSS8**	Might be an alternative entry portal for SARS-CoV-2 and contribute to and/or worsen lung infection/pneumonia [497]
**NMNAT1**	Nicotinamide-nucleotide adenylyltransferase (NMNAT), protective for injured axons (axon degeneration), but Inhibitory of axon regeneration (the Human Protein Atlas)
**AGRP**	Related to obesity [498]
**KYNU**	Metabolism. Biosynthesis of NAD cofactors from tryptophan through the kynurenine pathway (the Human Protein Atlas)
**CD300LF**	Inhibitory receptor for myeloid cells and mast cells (UniProtKB)

**BLMH**	Cytoplasmic cysteine peptidase. Inactivated the drug Bleomycin (UniProtKB)
**ZBTB16**	Transcriptional repressor and plays a role in myeloid maturation (UniProtKB)
**HS3ST3B1**	O-sulfation of Heparan sulfate (UniProtKB)
**HS6ST1**	O-sulfation of Heparan sulfate (UniProtKB)
**PAPPA**	Metalloproteinase which specifically cleaves IGFBP-4 and IGFBP-5, resulting in release of bound IGF. Involved in local proliferative processes such as wound healing [499]
**SCARB2**	Expressed in brain, heart, liver, lung and kidney, and at intercalated discs [500] & Wikipedia. Highly expressed in plasmacytoid DCs and involved in type I IFN production [501]
**DFFA**	Inhibitor of the caspase-activated DNase (UniProtKB)
**DCBLD2**	Also called ESDN, regulator of vascular remodeling and angiogenesis and inhibitor of insulin receptor signal transduction [502]
**THOP1**	Metabolism of peptides under 20 aa residues long, also plays a role in MHC-I antigen presentation and is secreted [503]
**PILRB**	Expressed on the cell surface of neutrophils, monocytes, macrophages, NK-cells, subset of T-cells and DCs. Triggering PILRB increases levels of IL-1β, TNFa and IL6 in serum or bronchoalveolar lavage fluid [504]. PILRβ is primary isoform displayed by NK cells [505]
**LAYN**	Cell adhesion. Upregulated in CD8+ Cytotoxic T cells [506]
**DPP7**	Expressed in quiescent lymphocytes (NCBI Gene)
**FKBP4**	Role in immunoregulatory gene expression in B- and T-Cells via IRF-4 inhibition [507]. Secreted in response to dsDNA [508]
**GALNT2**	Protein modification. Released by platelets [199]
**PHOSPHO1**	Phosphatase that has a high activity toward phosphoethanolamine (PEA) and phosphocholine (PCho). Involved in the generation of inorganic phosphate for bone mineralization (the Human Protein Atlas)
**PPP3R1**	Regulatory subunit of calcineurin, a calcium-dependent, calmodulin stimulated protein phosphatase (the Human Protein Atlas)
**CLSTN2**	Cell adhesion, modulate calcium-mediated postsynaptic signals (the Human Protein Atlas)
**KLB**	Bile acid synthesis and involved in activation of FGF21 protein has a protective effect on heart muscle cells [509]. Soluble, circulating form of β-klotho has been described but unclear function [510]
**SLAMF8**	A role in B-lineage commitment and/or modulation of signaling through the B-cell receptor (the Human Protein Atlas)
**VAMP5**	Participate in trafficking events that are associated with myogenesis, such as myoblast fusion and/or GLUT4 trafficking (the Human Protein Atlas)
**ACVRL1**	Receptor for BMP9/GDF2 and BMP10 and important regulator of normal blood vessel development (the Human Protein Atlas)
**PLIN1**	Perilipin, associate with the surface of lipid droplets [511]. Controls adipocyte lipid metabolism and its expression is elevated in obesity [512] Wikipedia

**CD300C**	Immunoregulatory. Inhibits T-cells [513]
**TRIM21**	TRIM21 is an intracellular antibody effector in the intracellular antibody-mediated proteolysis pathway. Involved in the regulation of innate immunity and the inflammatory response in response to IFNG/IFN-gamma [514] Wikipedia
**IDUA**	Heparin catabolism (the Human Protein Atlas)
**AGR3**	Regulation of ciliary beat frequency and mucociliary clearance in the airway, regulation of intracellular calcium in tracheal epithelial cells (UniProtKB)
**PON2**	Inflammation. Prevents LDL lipid peroxidation, reverses the oxidation of mildly oxidized LDL (UniProtKB)
**ENAH**	Induces the formation of F-actin rich outgrowths in fibroblasts (the Human Protein Atlas)
**EFNA4**	May play a role in the interaction between activated B-lymphocytes and dendritic cells (the Human Protein Atlas)
**DRAXIN**	Chemorepulsive axon guidance protein. Antagonist of Wnt signaling pathway [515]
**VSIG2**	Unknown
**NOMO1**	Part of a protein complex that participates in the Nodal signaling pathway in development (the Human Protein Atlas)
**CD302**	Receptor involved in cell adhesion and migration, as well as endocytosis and phagocytosis (the Human Protein Atlas)
**PCDH17**	Potential calcium-dependent cell-adhesion protein (UniProtKB)
**PREB**	A transcriptional regulator and is thought to be involved in some of the developmental abnormalities. Specifically activates the small GTPase SAR1B (UniProtKB and the Human Protein Atlas)
**AHCY**	Metabolism (UniProtKB)
**QDPR**	Enzyme for tetrahydrobiopterin biosynthesis (the Human Protein Atlas)
**HSD11B1**	Reduces cortisone to the active hormone cortisol (UniProtKB) that activates glucocorticoid receptors [516]
**DDAH1**	Regulation of nitric oxide generation, inhibit nitric oxide synthase activity (the Human Protein Atlas)
**GALNT10**	Protein modification (the Human Protein Atlas)
**FOSB**	Interacts with Jun proteins enhancing their DNA binding activities (the Human Protein Atlas)
**PFDN2**	A subunit of the Prefoldin complex, a chaperone complex in cytoplasm mainly involved in neurodegenerative diseases [517]
**ACP6**	Metabolism (UniProtKB)
**CFC1**	Development, vascular and heart. Maintenance of stem cells and stem cell renewal. Soluble form [518] inhibits the membrane-form function
**ALDH1A1**	Enzyme mainly expressed in liver (the Human Protein Atlas). Serum levels detected in cancer (e.g. breast cancer [519]). Presence in blood may be indicator of liver damage

**DDC**	Catalyzes the decarboxylation of L-3,4-dihydroxyphenylalanine (DOPA) to dopamine (the Human Protein Atlas)
**IGFBPL1**	Axonal Guidance. Circulating protein but not characterized (the Human Protein Atlas)
**ADAM22**	Regulation of cell adhesion and spreading and in inhibition of cell proliferation (the Human Protein Atlas)
**NTRK2**	Cell differentiation (the Human Protein Atlas)
**DNER**	Activator of the NOTCH1 pathway. Soluble form associates with inflammation [520]
**GPC5**	Binds growth factors and play a role in cell division and growth regulation, and cell migration (the Human Protein Atlas). Has secreted form but not functionally characterized
**PAG1**	Negatively regulates T-cell antigen receptor (UniProtKB)
**PRSS27**	Protease mainly expressed mainly in the pancreas (the Human Protein Atlas)
**BOC**	Cell-cell interactions between muscle precursor cells, Promotes differentiation of myogenic cells (the Human Protein Atlas). Elevated plasma BOC in heart failure but reduced with recovery [521]
**GFRA3**	Axon guidance (UniProtKB)
**EGLN1**	Primary regulator of HIF-1α steady state levels in the cell, involved in various hypoxia-influenced processes such as angiogenesis in retinal and cardiac functionality (UniProtKB)
**SIGLEC6**	Immunosuppressive function on CTLs by regulating the activity of mast cells [522]
**ANXA11**	Midbody formation and completion of the terminal phase of cytokinesis (UniProtKB)
**DSG3**	Cell-cell junctions between epithelial, myocardial, and certain other cell types (the Human Protein Atlas)
**USP8**	Regulatory role at the level of protein turnover by preventing degradation particularly during cell cycle. Regulate T-cell anergy mediated by RNF128 (UniProtKB)
**RASSF2**	Promote apoptosis and cell cycle arrest (UniProtKB)
**ITGA11**	Integrin alpha-11/beta-1 is a receptor for collagen (UniProtKB)
**LRRN1**	Inhibits the Fas/FasL pathway and suppresses the apoptosis [523]
**SULT1A1**	Metabolism (UniProtKB)
**DSG4**	Cell adhesion (the Human Protein Atlas)
**BID**	Pro-apoptotic Bcl member (UniProtKB)
**SIT1**	Immunity. Negatively regulates TCR-mediated signaling in T-cells. Involved in positive selection of T-cells (the Human Protein Atlas)
**NCF2**	Oxidase produces a burst of superoxide which is delivered to the lumen of the neutrophil phagosome (the Human Protein Atlas)
**SKAP1**	Activation of T-cells, TCR signaling (UniProtKB)
**F11R**	Ligand for integrin ITGAL/ITGB2 to forms cell junctions and involved in the transendothelial migration of leukocytes and neutrophils [524] and platelet aggregation [525]. High level of circulating F11R in atherosclerosis [526] and hypertension [527]

**SELE**	E-selectin mediates in the adhesion of blood neutrophils in cytokine-activated endothelium (UniProtKB). Serum levels associate with COVID-19 severity [528, 529]. sE-selectin associates with sepsis and coagulopathy [530, 531]
**SELP**	P-selectin is an integral membrane protein that mediates the adhesion of activated platelets and endothelial cells to neutrophils and monocytes and has been proposed as a drug target for COVID-19-related ARDS [532]. Serum levels are higher in COVID-19 patients supporting its role in coagulopathy [533]. sP-selectin associates with sepsis and coagulopathy [530]
**FCGR2A**	FcγRIIa is a low affinity receptor for the Fc region of immunoglobulins gamma. Binding to IgG initiates cellular responses against pathogens and soluble antigens (UniProtKB). Relevant to immunity in COVID-19 and other infections [534]. A soluble form has been described and may modulate the interaction between immune complexes and membrane-associated Fc gamma RII [535] and shown to inhibits rheumatoid factor binding to immune complexes [536]
**FCGR3B**	FcγRIIIb is a low affinity receptor for aggregated and monomeric IgG. Not capable to mediate antibody-dependent cytotoxicity and phagocytosis, thus serves as a trap for immune complexes in the peripheral circulation which does not activate neutrophils ((UniProtKB)
**SLAMF7**	Self-receptor involved in immune modulation including NK cell-mediated cytotoxicity [537], regulation of lymphocyte adhesion [538], and in macrophage super-activation with broad implications in pathology of acute and chronic inflammation including severe COVID-19 [539]. Soluble form (sSLAMF7) has been described in multiple myeloma to activate surface SLAMF7 [540, 541]
**FETUB**	Protease inhibitor required for egg fertilization and other functions including systemic inflammation (the Human Protein Atlas). Reduced level of this type-3 cystatin has been reported in plasma of severe COVID-19 patients [542, 543]. Fetuin-B is a key partner in the recovery phase of an acute inflammatory response [544]
**FABP4**	Lipid transport protein in adipocytes. Circulating FABP4 associates with poor outcomes in cardiovascular disease, stroke and chronic kidney disease [545-547]
**LTBP2**	May play an integral structural role in elastic-fiber architectural organization and/or assembly [548]. LTBP2 is secreted from lung myofibroblasts in response to TGFβ1 and higher serum level in idiopathic pulmonary fibrosis patients versus healthy controls [549]
**LGALS3**	Galectin-3 has several roles including in innate immune responses against pathogens such as infection which leads to its secretion to act as pattern recognition protein and recruiting neutrophils [550]. In addition to its role in viral infection [551], Galectin-3 has been proposed as a biomarker for COVID-19 for its role in fibrosis and inflammation [552]
**CDSN**	Important for the epidermal barrier integrity (UniProtKB). Found in corneodesmosomes, which localize to human epidermis and other cornified squamous epithelia and its loss leads to skin barrier defect [553]. No literature about its presence in circulation
**CNTN5**	Contactins mediate cell surface interactions during nervous system development (UniProtKB)
**IL22RA1**	IL22RA1 and IL10RB form the receptor for IL22 and one of the receptors for IL20 and IL24 to enable signaling via JAK/STAT pathways (UniProtKB). A soluble form has been detected plasma [554]. IL22RA2 is a related homologue which is a secreted (soluble) decoy receptor for IL22 inhibiting its function [555, 556] in wound healing and in protection against microbes in non-hematopoietic cells [557]. By similarity, sIL22RA1 may be a decoy receptor for IL22
**NGF**	Activates cellular signaling cascades to regulate neuronal proliferation, differentiation, and survival (UniProtKB)

Supplementary References

1. Dienz, O. and M. Rincon, *The effects of IL-6 on CD4 T cell responses.* Clin Immunol, 2009. **130**(1): p. 27-33.https://www.ncbi.nlm.nih.gov/pubmed/18845487
2. Rose-John, S., *IL-6 trans-signaling via the soluble IL-6 receptor: importance for the pro-inflammatory activities of IL-6.* Int J Biol Sci, 2012. **8**(9): p. 1237-47.https://www.ncbi.nlm.nih.gov/pubmed/23136552
3. Jones, G.W., et al., *Loss of CD4+ T cell IL-6R expression during inflammation underlines a role for IL-6 trans signaling in the local maintenance of Th17 cells.* J Immunol, 2010. **184**(4): p. 2130-9.https://www.ncbi.nlm.nih.gov/pubmed/20083667
4. Yoshimura, T., et al., *Two-sided roles of IL-27: induction of Th1 differentiation on naive CD4+ T cells versus suppression of proinflammatory cytokine production including IL-23-induced IL-17 on activated CD4+ T cells partially through STAT3-dependent mechanism.* J Immunol, 2006. **177**(8): p. 5377-85.https://www.ncbi.nlm.nih.gov/pubmed/17015723
5. Morrow, K.N., C.M. Coopersmith, and M.L. Ford, *IL-17, IL-27, and IL-33: A Novel Axis Linked to Immunological Dysfunction During Sepsis.* Front Immunol, 2019. **10**: p. 1982.https://www.ncbi.nlm.nih.gov/pubmed/31507598
6. Athie-Morales, V., et al., *Sustained IL-12 signaling is required for Th1 development.* J Immunol, 2004. **172**(1): p. 61-9.https://www.ncbi.nlm.nih.gov/pubmed/14688310
7. Ria, F., G. Penna, and L. Adorini, *Th1 cells induce and Th2 inhibit antigen-dependent IL-12 secretion by dendritic cells.* Eur J Immunol, 1998. **28**(6): p. 2003-16.https://www.ncbi.nlm.nih.gov/pubmed/9645382
8. Schweigert, O., et al., *Soluble T cell immunoglobulin and mucin domain (TIM)-1 and -4 generated by A Disintegrin And Metalloprotease (ADAM)-10 and -17 bind to phosphatidylserine.* Biochim Biophys Acta, 2014. **1843**(2): p. 275-87.https://www.ncbi.nlm.nih.gov/pubmed/24286866
9. Ichimura, T., et al., *KIM-1/TIM-1 is a Receptor for SARS-CoV-2 in Lung and Kidney.* medRxiv, 2020.https://www.ncbi.nlm.nih.gov/pubmed/32995803
10. Curtiss, M. and J. Colgan, *The role of the T-cell costimulatory molecule Tim-1 in the immune response.* Immunol Res, 2007. **39**(1-3): p. 52-61.https://www.ncbi.nlm.nih.gov/pubmed/17917055
11. Song, L., et al., *Association of TIM-1 (T-Cell Immunoglobulin and Mucin Domain 1) With Incidence of Stroke.* Arterioscler Thromb Vasc Biol, 2020. **40**(7): p. 1777-1786.https://www.ncbi.nlm.nih.gov/pubmed/32460577
12. Schulz, C.A., et al., *Plasma kidney injury molecule-1 (p-KIM-1) levels and deterioration of kidney function over 16 years.* Nephrol Dial Transplant, 2020. **35**(2): p. 265-273.https://www.ncbi.nlm.nih.gov/pubmed/30629206
13. Stenemo, M., et al., *Circulating proteins as predictors of incident heart failure in the elderly.* Eur J Heart Fail, 2018. **20**(1): p. 55-62.https://www.ncbi.nlm.nih.gov/pubmed/28967680
14. Davis, S.M. and K.R. Pennypacker, *The role of the leukemia inhibitory factor receptor in neuroprotective signaling.* Pharmacol Ther, 2018. **183**: p. 50-57.https://www.ncbi.nlm.nih.gov/pubmed/28827150
15. Metcalfe, S.M., *LIF in the regulation of T-cell fate and as a potential therapeutic.* Genes Immun, 2011. **12**(3): p. 157-68.https://www.ncbi.nlm.nih.gov/pubmed/21368774
16. Gratchev, A., et al., *The receptor for interleukin-17E is induced by Th2 cytokines in antigen-presenting cells.* Scand J Immunol, 2004. **60**(3): p. 233-7.https://www.ncbi.nlm.nih.gov/pubmed/15320879
17. Gordon, D.E., et al., *Comparative host-coronavirus protein interaction networks reveal pan-viral disease mechanisms.* Science, 2020. **370**(6521).https://www.ncbi.nlm.nih.gov/pubmed/33060197
18. Sohda, M., et al., *Identification of a soluble isoform of human IL-17RA generated by alternative splicing.* Cytokine, 2013. **64**(3): p. 642-5.https://www.ncbi.nlm.nih.gov/pubmed/24084331
19. Li, Q., et al., *Recruitment of CCR6-expressing Th17 cells by CCL20 secreted from plasmin-stimulated macrophages.* Acta Biochim Biophys Sin (Shanghai), 2013. **45**(7): p. 593-600.https://www.ncbi.nlm.nih.gov/pubmed/23681234

20. Liu, H., et al., *Enhanced production of secretory glycoprotein VSTM1-v2 with mouse IgGkappa signal peptide in optimized HEK293F transient transfection.* J Biosci Bioeng, 2016. **121**(2): p. 133-9.https://www.ncbi.nlm.nih.gov/pubmed/26140918
21. Vasilyev, F.F., A.N. Silkov, and S.V. Sennikov, *Relationship between interleukin-1 type 1 and 2 receptor gene polymorphisms and the expression level of membrane-bound receptors.* Cell Mol Immunol, 2015. **12**(2): p. 222-30.https://www.ncbi.nlm.nih.gov/pubmed/24976267
22. Chung, Y., et al., *Critical regulation of early Th17 cell differentiation by interleukin-1 signaling.* Immunity, 2009. **30**(4): p. 576-87.https://www.ncbi.nlm.nih.gov/pubmed/19362022
23. Santarlasci, V., et al., *IL-1 and T Helper Immune Responses.* Front Immunol, 2013. **4**: p. 182.https://www.ncbi.nlm.nih.gov/pubmed/23874332
24. Peters, V.A., J.J. Joesting, and G.G. Freund, *IL-1 receptor 2 (IL-1R2) and its role in immune regulation.* Brain Behav Immun, 2013. **32**: p. 1-8.https://www.ncbi.nlm.nih.gov/pubmed/23195532
25. Altara, R., et al., *Conflicting vascular and metabolic impact of the IL-33/sST2 axis.* Cardiovasc Res, 2018. **114**(12): p. 1578-1594.https://www.ncbi.nlm.nih.gov/pubmed/29982301
26. Alves-Filho, J.C., et al., *Interleukin-33 attenuates sepsis by enhancing neutrophil influx to the site of infection.* Nat Med, 2010. **16**(6): p. 708-12.https://www.ncbi.nlm.nih.gov/pubmed/20473304
27. Liew, F.Y., J.P. Girard, and H.R. Turnquist, *Interleukin-33 in health and disease.* Nat Rev Immunol, 2016. **16**(11): p. 676-689.https://www.ncbi.nlm.nih.gov/pubmed/27640624
28. Vigne, S., et al., *IL-36 signaling amplifies Th1 responses by enhancing proliferation and Th1 polarization of naive CD4+ T cells.* Blood, 2012. **120**(17): p. 3478-87.https://www.ncbi.nlm.nih.gov/pubmed/22968459
29. Yi, G., et al., *Structural and Functional Attributes of the Interleukin-36 Receptor.* J Biol Chem, 2016. **291**(32): p. 16597-609.https://www.ncbi.nlm.nih.gov/pubmed/27307043
30. Zaiss, D.M.W., et al., *Emerging functions of amphiregulin in orchestrating immunity, inflammation, and tissue repair.* Immunity, 2015. **42**(2): p. 216-226.https://www.ncbi.nlm.nih.gov/pubmed/25692699
31. Morimoto, Y., et al., *Amphiregulin-Producing Pathogenic Memory T Helper 2 Cells Instruct Eosinophils to Secrete Osteopontin and Facilitate Airway Fibrosis.* Immunity, 2018. **49**(1): p. 134-150 e6.https://www.ncbi.nlm.nih.gov/pubmed/29958800
32. Novick, D., et al., *Interleukin-18 binding protein: a novel modulator of the Th1 cytokine response.* Immunity, 1999. **10**(1): p. 127-36.https://www.ncbi.nlm.nih.gov/pubmed/10023777
33. Tominaga, K., et al., *IL-12 synergizes with IL-18 or IL-1beta for IFN-gamma production from human T cells.* Int Immunol, 2000. **12**(2): p. 151-60.https://www.ncbi.nlm.nih.gov/pubmed/10653850
34. Reznikov, L.L., et al., *The combination of soluble IL-18Ralpha and IL-18Rbeta chains inhibits IL-18-induced IFN-gamma.* J Interferon Cytokine Res, 2002. **22**(5): p. 593-601.https://www.ncbi.nlm.nih.gov/pubmed/12060498
35. Takei, S., et al., *Soluble interleukin-18 receptor complex is a novel biomarker in rheumatoid arthritis.* Arthritis Res Ther, 2011. **13**(2): p. R52.https://www.ncbi.nlm.nih.gov/pubmed/21435242
36. Wang, K.X. and D.T. Denhardt, *Osteopontin: role in immune regulation and stress responses.* Cytokine Growth Factor Rev, 2008. **19**(5-6): p. 333-45.https://www.ncbi.nlm.nih.gov/pubmed/18952487
37. Singh, M., S. Dalal, and K. Singh, *Osteopontin: At the cross-roads of myocyte survival and myocardial function.* Life Sci, 2014. **118**(1): p. 1-6.https://www.ncbi.nlm.nih.gov/pubmed/25265596
38. Lu, L., et al., *A Potential Role of Interleukin 10 in COVID-19 Pathogenesis.* Trends Immunol, 2021. **42**(1): p. 3-5.https://www.ncbi.nlm.nih.gov/pubmed/33214057
39. Stamm, H., J. Wellbrock, and W. Fiedler, *Interaction of PVR/PVRL2 with TIGIT/DNAM-1 as a novel immune checkpoint axis and therapeutic target in cancer.* Mamm Genome, 2018. **29**(11-12): p. 694-702.https://www.ncbi.nlm.nih.gov/pubmed/30132062
40. Okumura, G., et al., *Tumor-derived soluble CD155 inhibits DNAM-1-mediated antitumor activity of natural killer cells.* J Exp Med, 2020. **217**(4).https://www.ncbi.nlm.nih.gov/pubmed/32040157

41. Chang, G.W., et al., *The Adhesion G Protein-Coupled Receptor GPR56/ADGRG1 Is an Inhibitory Receptor on Human NK Cells.* Cell Rep, 2016. **15**(8): p. 1757-70.https://www.ncbi.nlm.nih.gov/pubmed/27184850
42. Zhu, C., et al., *The Tim-3 ligand galectin-9 negatively regulates T helper type 1 immunity.* Nat Immunol, 2005. **6**(12): p. 1245-52.https://www.ncbi.nlm.nih.gov/pubmed/16286920
43. Anderson, A.C., N. Joller, and V.K. Kuchroo, *Lag-3, Tim-3, and TIGIT: Co-inhibitory Receptors with Specialized Functions in Immune Regulation.* Immunity, 2016. **44**(5): p. 989-1004.https://www.ncbi.nlm.nih.gov/pubmed/27192565
44. Vaitaitis, G.M. and D.H. Wagner, Jr., *Galectin-9 controls CD40 signaling through a Tim-3 independent mechanism and redirects the cytokine profile of pathogenic T cells in autoimmunity.* PLoS One, 2012. **7**(6): p. e38708.https://www.ncbi.nlm.nih.gov/pubmed/22685601
45. Le Tortorec, A., S. Willey, and S.J. Neil, *Antiviral inhibition of enveloped virus release by tetherin/BST-2: action and counteraction.* Viruses, 2011. **3**(5): p. 520-40.https://www.ncbi.nlm.nih.gov/pubmed/21994744
46. El-Sherbiny, Y.M., et al., *B Cell Tetherin: A Flow Cytometric Cell-Specific Assay for Response to Type I Interferon Predicts Clinical Features and Flares in Systemic Lupus Erythematosus.* Arthritis Rheumatol, 2020. **72**(5): p. 769-779.https://www.ncbi.nlm.nih.gov/pubmed/31804007
47. Cai, D., et al., *Up-regulation of bone marrow stromal protein 2 (BST2) in breast cancer with bone metastasis.* BMC Cancer, 2009. **9**: p. 102.https://www.ncbi.nlm.nih.gov/pubmed/19338666
48. Chiang, S.F., et al., *Bone Marrow Stromal Antigen 2 Is a Novel Plasma Biomarker and Prognosticator for Colorectal Carcinoma: A Secretome-Based Verification Study.* Dis Markers, 2015. **2015**: p. 874054.https://www.ncbi.nlm.nih.gov/pubmed/26494939
49. Martin-Sancho, L., et al., *Functional landscape of SARS-CoV-2 cellular restriction.* Mol Cell, 2021. **81**(12): p. 2656-2668 e8.https://www.ncbi.nlm.nih.gov/pubmed/33930332
50. Royster, W., P. Wang, and M. Aziz, *The Role of Siglec-G on Immune Cells in Sepsis.* Front Immunol, 2021. **12**: p. 621627.https://www.ncbi.nlm.nih.gov/pubmed/33708213
51. Kitzig, F., et al., *Cloning of two new splice variants of Siglec-10 and mapping of the interaction between Siglec-10 and SHP-1.* Biochem Biophys Res Commun, 2002. **296**(2): p. 355-62.https://www.ncbi.nlm.nih.gov/pubmed/12163025
52. Lin, H., et al., *Stanniocalcin 1 is a phagocytosis checkpoint driving tumor immune resistance.* Cancer Cell, 2021. **39**(4): p. 480-493 e6.https://www.ncbi.nlm.nih.gov/pubmed/33513345
53. Lee-Sundlov, M.M., et al., *Circulating blood and platelets supply glycosyltransferases that enable extrinsic extracellular glycosylation.* Glycobiology, 2017. **27**(2): p. 188-198.https://www.ncbi.nlm.nih.gov/pubmed/27798070
54. Lin, W.D., et al., *Sialylation of CD55 by ST3GAL1 Facilitates Immune Evasion in Cancer.* Cancer Immunol Res, 2021. **9**(1): p. 113-122.https://www.ncbi.nlm.nih.gov/pubmed/33177111
55. Anderson, K.J. and R.L. Allen, *Regulation of T-cell immunity by leucocyte immunoglobulin-like receptors: innate immune receptors for self on antigen-presenting cells.* Immunology, 2009. **127**(1): p. 8-17.https://www.ncbi.nlm.nih.gov/pubmed/19368561
56. Jones, D.C., et al., *Alternative mRNA splicing creates transcripts encoding soluble proteins from most LILR genes.* Eur J Immunol, 2009. **39**(11): p. 3195-206.https://www.ncbi.nlm.nih.gov/pubmed/19658091
57. Suciu-Foca, N., et al., *Soluble Ig-like transcript 3 inhibits tumor allograft rejection in humanized SCID mice and T cell responses in cancer patients.* J Immunol, 2007. **178**(11): p. 7432-41.https://www.ncbi.nlm.nih.gov/pubmed/17513794
58. Vlad, G., et al., *Immunoglobulin-like transcript 3-Fc suppresses T-cell responses to allogeneic human islet transplants in hu-NOD/SCID mice.* Diabetes, 2008. **57**(7): p. 1878-86.https://www.ncbi.nlm.nih.gov/pubmed/18420485
59. Karabulut, M., et al., *Serum nectin-2 levels are diagnostic and prognostic in patients with colorectal carcinoma.* Clin Transl Oncol, 2016. **18**(2): p. 160-71.https://www.ncbi.nlm.nih.gov/pubmed/26184725

60. Erturk, K., et al., *Serum nectin-2 and nectin-4 are diagnostic in lung cancer: which is superior?* Wien Klin Wochenschr, 2019. **131**(17-18): p. 419-426.https://www.ncbi.nlm.nih.gov/pubmed/31440821
61. Hartnell, A., et al., *Characterization of human sialoadhesin, a sialic acid binding receptor expressed by resident and inflammatory macrophage populations.* Blood, 2001. **97**(1): p. 288-96.https://www.ncbi.nlm.nih.gov/pubmed/11133773
62. Oliveira, J.J., et al., *The plasma biomarker soluble SIGLEC-1 is associated with the type I interferon transcriptional signature, ethnic background and renal disease in systemic lupus erythematosus.* Arthritis Res Ther, 2018. **20**(1): p. 152.https://www.ncbi.nlm.nih.gov/pubmed/30053827
63. Crocker, P.R., et al., *Sialoadhesin, a macrophage sialic acid binding receptor for haemopoietic cells with 17 immunoglobulin-like domains.* EMBO J, 1994. **13**(19): p. 4490-503.https://www.ncbi.nlm.nih.gov/pubmed/7925291
64. O'Neill, A.S., T.K. van den Berg, and G.E. Mullen, *Sialoadhesin - a macrophage-restricted marker of immunoregulation and inflammation.* Immunology, 2013. **138**(3): p. 198-207.https://www.ncbi.nlm.nih.gov/pubmed/23181380
65. Kerr, S.C., et al., *Endoglycan, a member of the CD34 family of sialomucins, is a ligand for the vascular selectins.* J Immunol, 2008. **181**(2): p. 1480-90.https://www.ncbi.nlm.nih.gov/pubmed/18606703
66. Kuhn, P.H., et al., *Systematic substrate identification indicates a central role for the metalloprotease ADAM10 in axon targeting and synapse function.* Elife, 2016. **5**.https://www.ncbi.nlm.nih.gov/pubmed/26802628
67. Hsia, H.E., et al., *Endoglycan (PODXL2) is proteolytically processed by ADAM10 (a disintegrin and metalloprotease 10) and controls neurite branching in primary neurons.* FASEB J, 2021. **35**(9): p. e21813.https://www.ncbi.nlm.nih.gov/pubmed/34390512
68. Sabbatino, F., et al., *PD-L1 Dysregulation in COVID-19 Patients.* Front Immunol, 2021. **12**: p. 695242.https://www.ncbi.nlm.nih.gov/pubmed/34163490
69. Jordan, W.J., et al., *Modulation of the human cytokine response by interferon lambda-1 (IFN-lambda1/IL-29).* Genes Immun, 2007. **8**(1): p. 13-20.https://www.ncbi.nlm.nih.gov/pubmed/17082759
70. Kelm, N.E., et al., *The role of IL-29 in immunity and cancer.* Crit Rev Oncol Hematol, 2016. **106**: p. 91-8.https://www.ncbi.nlm.nih.gov/pubmed/27637354
71. Lazear, H.M., T.J. Nice, and M.S. Diamond, *Interferon-lambda: Immune Functions at Barrier Surfaces and Beyond.* Immunity, 2015. **43**(1): p. 15-28.https://www.ncbi.nlm.nih.gov/pubmed/26200010
72. Wang, J.M., et al., *Insights into IL-29: Emerging role in inflammatory autoimmune diseases.* J Cell Mol Med, 2019. **23**(12): p. 7926-7932.https://www.ncbi.nlm.nih.gov/pubmed/31578802
73. Santer, D.M., et al., *Differential expression of interferon-lambda receptor 1 splice variants determines the magnitude of the antiviral response induced by interferon-lambda 3 in human immune cells.* PLoS Pathog, 2020. **16**(4): p. e1008515.https://www.ncbi.nlm.nih.gov/pubmed/32353085
74. De Marzi, M.C., et al., *Peptidoglycan recognition protein-peptidoglycan complexes increase monocyte/macrophage activation and enhance the inflammatory response.* Immunology, 2015. **145**(3): p. 429-42.https://www.ncbi.nlm.nih.gov/pubmed/25752767
75. Sharapova, T.N., et al., *Innate immunity protein Tag7 (PGRP-S) activates lymphocytes capable of Fasl-Fas-dependent contact killing of virus-infected cells.* IUBMB Life, 2017. **69**(12): p. 971-977.https://www.ncbi.nlm.nih.gov/pubmed/29083508
76. Klimczak-Tomaniak, D., et al., *Temporal patterns of macrophage- and neutrophil-related markers are associated with clinical outcome in heart failure patients.* ESC Heart Fail, 2020. **7**(3): p. 1190-1200.https://www.ncbi.nlm.nih.gov/pubmed/32196993
77. Han, Y., et al., *Circulating PGLYRP1 Levels as a Potential Biomarker for Coronary Artery Disease and Heart Failure.* J Cardiovasc Pharmacol, 2021. **77**(5): p. 578-585.https://www.ncbi.nlm.nih.gov/pubmed/33760799
78. Yashin, D.V., et al., *Tag7 (PGLYRP1) in Complex with Hsp70 Induces Alternative Cytotoxic Processes in Tumor Cells via TNFR1 Receptor.* J Biol Chem, 2015. **290**(35): p. 21724-31.https://www.ncbi.nlm.nih.gov/pubmed/26183779

79. Piedra-Quintero, Z.L., et al., *CD38: An Immunomodulatory Molecule in Inflammation and Autoimmunity.* Front Immunol, 2020. **11**: p. 597959.https://www.ncbi.nlm.nih.gov/pubmed/33329591
80. Glaria, E. and A.F. Valledor, *Roles of CD38 in the Immune Response to Infection.* Cells, 2020. **9**(1).https://www.ncbi.nlm.nih.gov/pubmed/31963337
81. Funaro, A., et al., *Identification and characterization of an active soluble form of human CD38 in normal and pathological fluids.* Int Immunol, 1996. **8**(11): p. 1643-50.https://www.ncbi.nlm.nih.gov/pubmed/8943558
82. Russell, S.E., et al., *Soluble IL-2Ralpha (sCD25) exacerbates autoimmunity and enhances the development of Th17 responses in mice.* PLoS One, 2012. **7**(10): p. e47748.https://www.ncbi.nlm.nih.gov/pubmed/23077668
83. Durda, P., et al., *Plasma Levels of Soluble Interleukin-2 Receptor alpha: Associations With Clinical Cardiovascular Events and Genome-Wide Association Scan.* Arterioscler Thromb Vasc Biol, 2015. **35**(10): p. 2246-53.https://www.ncbi.nlm.nih.gov/pubmed/26293465
84. Ueland, T., et al., *Elevated plasma sTIM-3 levels in patients with severe COVID-19.* J Allergy Clin Immunol, 2021. **147**(1): p. 92-98.https://www.ncbi.nlm.nih.gov/pubmed/32971109
85. Maurya, R., et al., *Leptin Functions in Infectious Diseases.* Front Immunol, 2018. **9**: p. 2741.https://www.ncbi.nlm.nih.gov/pubmed/30534129
86. Gruzdeva, O., et al., *Leptin resistance: underlying mechanisms and diagnosis.* Diabetes Metab Syndr Obes, 2019. **12**: p. 191-198.https://www.ncbi.nlm.nih.gov/pubmed/30774404
87. Spassov, D.S., et al., *Phosphorylation of Trask by Src kinases inhibits integrin clustering and functions in exclusion with focal adhesion signaling.* Mol Cell Biol, 2011. **31**(4): p. 766-82.https://www.ncbi.nlm.nih.gov/pubmed/21189288
88. Casar, B., et al., *Blocking of CDCP1 cleavage in vivo prevents Akt-dependent survival and inhibits metastatic colonization through PARP1-mediated apoptosis of cancer cells.* Oncogene, 2012. **31**(35): p. 3924-38.https://www.ncbi.nlm.nih.gov/pubmed/22179830
89. Enyindah-Asonye, G., et al., *CD318 is a ligand for CD6.* Proc Natl Acad Sci U S A, 2017. **114**(33): p. E6912-E6921.https://www.ncbi.nlm.nih.gov/pubmed/28760953
90. Ruth, J.H., et al., *CD6 is a target for cancer immunotherapy.* JCI Insight, 2021. **6**(5).https://www.ncbi.nlm.nih.gov/pubmed/33497367
91. Fong, K.P., et al., *Deciphering the human platelet sheddome.* Blood, 2011. **117**(1): p. e15-26.https://www.ncbi.nlm.nih.gov/pubmed/20962327
92. Carrasco, E., et al., *Human CD6 Down-Modulation following T-Cell Activation Compromises Lymphocyte Survival and Proliferative Responses.* Front Immunol, 2017. **8**: p. 769.https://www.ncbi.nlm.nih.gov/pubmed/28713387
93. Kumanogoh, A. and H. Kikutani, *Immunological functions of the neuropilins and plexins as receptors for semaphorins.* Nat Rev Immunol, 2013. **13**(11): p. 802-14.https://www.ncbi.nlm.nih.gov/pubmed/24319778
94. Zhu, L., et al., *Regulated surface expression and shedding support a dual role for semaphorin 4D in platelet responses to vascular injury.* Proc Natl Acad Sci U S A, 2007. **104**(5): p. 1621-6.https://www.ncbi.nlm.nih.gov/pubmed/17244710
95. Roney, K., E. Holl, and J. Ting, *Immune plexins and semaphorins: old proteins, new immune functions.* Protein Cell, 2013. **4**(1): p. 17-26.https://www.ncbi.nlm.nih.gov/pubmed/23307780
96. Peacock, J.W., et al., *SEMA3C drives cancer growth by transactivating multiple receptor tyrosine kinases via Plexin B1.* EMBO Mol Med, 2018. **10**(2): p. 219-238.https://www.ncbi.nlm.nih.gov/pubmed/29348142
97. Elhabazi, A., et al., *Structure and function of the immune semaphorin CD100/SEMA4D.* Crit Rev Immunol, 2003. **23**(1-2): p. 65-81.https://www.ncbi.nlm.nih.gov/pubmed/12906260
98. Ito, D., et al., *mTOR Complex Signaling through the SEMA4A-Plexin B2 Axis Is Required for Optimal Activation and Differentiation of CD8+ T Cells.* J Immunol, 2015. **195**(3): p. 934-43.https://www.ncbi.nlm.nih.gov/pubmed/26116513

99. Gras, C., et al., *Secreted semaphorin 5A activates immune effector cells and is a biomarker for rheumatoid arthritis.* Arthritis Rheumatol, 2014. **66**(6): p. 1461-71.https://www.ncbi.nlm.nih.gov/pubmed/24585544
100. Rajabinejad, M., et al., *Semaphorin 4A, 4C, and 4D: Function comparison in the autoimmunity, allergy, and cancer.* Gene, 2020. **746**: p. 144637.https://www.ncbi.nlm.nih.gov/pubmed/32244055
101. Xue, D., et al., *Semaphorin 4C: A Novel Component of B-Cell Polarization in Th2-Driven Immune Responses.* Front Immunol, 2016. **7**: p. 558.https://www.ncbi.nlm.nih.gov/pubmed/28003812
102. Zagai, U., et al., *Eosinophil cationic protein stimulates migration of human lung fibroblasts in vitro.* Scand J Immunol, 2009. **69**(4): p. 381-6.https://www.ncbi.nlm.nih.gov/pubmed/19284504
103. Venge, P., et al., *Eosinophil cationic protein (ECP): molecular and biological properties and the use of ECP as a marker of eosinophil activation in disease.* Clin Exp Allergy, 1999. **29**(9): p. 1172-86.https://www.ncbi.nlm.nih.gov/pubmed/10469025
104. Petretto, A., et al., *Neutrophil extracellular traps (NET) induced by different stimuli: A comparative proteomic analysis.* PLoS One, 2019. **14**(7): p. e0218946.https://www.ncbi.nlm.nih.gov/pubmed/31283757
105. Bystrom, J., K. Amin, and D. Bishop-Bailey, *Analysing the eosinophil cationic protein--a clue to the function of the eosinophil granulocyte.* Respir Res, 2011. **12**: p. 10.https://www.ncbi.nlm.nih.gov/pubmed/21235798
106. Waldhauer, I. and A. Steinle, *Proteolytic release of soluble UL16-binding protein 2 from tumor cells.* Cancer Res, 2006. **66**(5): p. 2520-6.https://www.ncbi.nlm.nih.gov/pubmed/16510567
107. Lopez-Soto, A., et al., *NKG2D signaling in cancer immunosurveillance.* Int J Cancer, 2015. **136**(8): p. 1741-50.https://www.ncbi.nlm.nih.gov/pubmed/24615398
108. Jung, T., et al., *Soluble human interleukin-4 receptor is produced by activated T cells under the control of metalloproteinases.* Int Arch Allergy Immunol, 1999. **119**(1): p. 23-30.https://www.ncbi.nlm.nih.gov/pubmed/10341317
109. ElKassar, N. and R.E. Gress, *An overview of IL-7 biology and its use in immunotherapy.* J Immunotoxicol, 2010. **7**(1): p. 1-7.https://www.ncbi.nlm.nih.gov/pubmed/20017587
110. Bikker, A., et al., *Interleukin-7: a key mediator in T cell-driven autoimmunity, inflammation, and tissue destruction.* Curr Pharm Des, 2012. **18**(16): p. 2347-56.https://www.ncbi.nlm.nih.gov/pubmed/22390698
111. Wang, G.L., et al., *Serum IP-10 and IL-7 levels are associated with disease severity of coronavirus disease 2019.* Cytokine, 2021. **142**: p. 155500.https://www.ncbi.nlm.nih.gov/pubmed/33810947
112. Benyamine, A., et al., *BTN3A is a prognosis marker and a promising target for Vgamma9Vdelta2 T cells based-immunotherapy in pancreatic ductal adenocarcinoma (PDAC).* Oncoimmunology, 2017. **7**(1): p. e1372080.https://www.ncbi.nlm.nih.gov/pubmed/29296524
113. Blazquez, J.L., et al., *New Insights Into the Regulation of gammadelta T Cells by BTN3A and Other BTN/BTNL in Tumor Immunity.* Front Immunol, 2018. **9**: p. 1601.https://www.ncbi.nlm.nih.gov/pubmed/30050536
114. Zen, K., et al., *Neutrophil migration across tight junctions is mediated by adhesive interactions between epithelial coxsackie and adenovirus receptor and a junctional adhesion molecule-like protein on neutrophils.* Mol Biol Cell, 2005. **16**(6): p. 2694-703.https://www.ncbi.nlm.nih.gov/pubmed/15800062
115. Luissint, A.C., et al., *JAM-L-mediated leukocyte adhesion to endothelial cells is regulated in cis by alpha4beta1 integrin activation.* J Cell Biol, 2008. **183**(6): p. 1159-73.https://www.ncbi.nlm.nih.gov/pubmed/19064666
116. Witherden, D.A., et al., *The junctional adhesion molecule JAML is a costimulatory receptor for epithelial gammadelta T cell activation.* Science, 2010. **329**(5996): p. 1205-10.https://www.ncbi.nlm.nih.gov/pubmed/20813954
117. He, Y.W., et al., *The extracellular matrix protein mindin is a pattern-recognition molecule for microbial pathogens.* Nat Immunol, 2004. **5**(1): p. 88-97.https://www.ncbi.nlm.nih.gov/pubmed/14691481

118. Sperk, M., et al., *Plasma soluble factor following two decades prolonged suppressive antiretroviral therapy in HIV-1-positive males: A cross-sectional study.* Medicine (Baltimore), 2018. **97**(5): p. e9759.https://www.ncbi.nlm.nih.gov/pubmed/29384862
119. Zeiger, R.S. and H.R. Colten, *Histaminase release from human eosinophils.* J Immunol, 1977. **118**(2): p. 540-3.https://www.ncbi.nlm.nih.gov/pubmed/402420
120. Herman, J.J., et al., *Complement-dependent histaminase release from human granulocytes.* J Clin Invest, 1979. **63**(6): p. 1195-202.https://www.ncbi.nlm.nih.gov/pubmed/109469
121. Herman, J.J., *Eosinophil diamine oxidase activity in acute inflammation in humans.* Agents Actions, 1982. **12**(1-2): p. 46-8.https://www.ncbi.nlm.nih.gov/pubmed/6805265
122. Zeiger, R.S., F.J. Twarog, and H.R. Colten, *Histaminase release from human granulocytes.* J Exp Med, 1976. **144**(4): p. 1049-61.https://www.ncbi.nlm.nih.gov/pubmed/824399
123. Lee, M.M., et al., *Platelets support extracellular sialylation by supplying the sugar donor substrate.* J Biol Chem, 2014. **289**(13): p. 8742-8.https://www.ncbi.nlm.nih.gov/pubmed/24550397
124. Jones, M.B., *IgG and leukocytes: Targets of immunomodulatory alpha2,6 sialic acids.* Cell Immunol, 2018. **333**: p. 58-64.https://www.ncbi.nlm.nih.gov/pubmed/29685495
125. Holdbrooks, A.T., et al., *Regulation of inflammatory signaling by the ST6Gal-I sialyltransferase.* PLoS One, 2020. **15**(11): p. e0241850.https://www.ncbi.nlm.nih.gov/pubmed/33166339
126. Irons, E.E., P.R. Punch, and J.T.Y. Lau, *Blood-Borne ST6GAL1 Regulates Immunoglobulin Production in B Cells.* Front Immunol, 2020. **11**: p. 617.https://www.ncbi.nlm.nih.gov/pubmed/32391003
127. Binici, J., et al., *A soluble fragment of the tumor antigen BCL2-associated athanogene 6 (BAG-6) is essential and sufficient for inhibition of NKp30 receptor-dependent cytotoxicity of natural killer cells.* J Biol Chem, 2013. **288**(48): p. 34295-303.https://www.ncbi.nlm.nih.gov/pubmed/24133212
128. Pazina, T., et al., *Regulation of the Functions of Natural Cytotoxicity Receptors by Interactions with Diverse Ligands and Alterations in Splice Variant Expression.* Front Immunol, 2017. **8**: p. 369.https://www.ncbi.nlm.nih.gov/pubmed/28424697
129. Reiners, K.S., et al., *Soluble ligands for NK cell receptors promote evasion of chronic lymphocytic leukemia cells from NK cell anti-tumor activity.* Blood, 2013. **121**(18): p. 3658-65.https://www.ncbi.nlm.nih.gov/pubmed/23509156
130. Beinhauer, B.G., et al., *Interleukin 10 regulates cell surface and soluble LIR-2 (CD85d) expression on dendritic cells resulting in T cell hyporesponsiveness in vitro.* Eur J Immunol, 2004. **34**(1): p. 74-80.https://www.ncbi.nlm.nih.gov/pubmed/14971032
131. Hitomi, K., et al., *An immunoglobulin-like receptor, Allergin-1, inhibits immunoglobulin E-mediated immediate hypersensitivity reactions.* Nat Immunol, 2010. **11**(7): p. 601-7.https://www.ncbi.nlm.nih.gov/pubmed/20526344
132. Salomonsson, M., et al., *Circulating mast cell progenitors correlate with reduced lung function in allergic asthma.* Clin Exp Allergy, 2019. **49**(6): p. 874-882.https://www.ncbi.nlm.nih.gov/pubmed/30892731
133. Delaveris, C.S., et al., *Synthetic Siglec-9 Agonists Inhibit Neutrophil Activation Associated with COVID-19.* ACS Cent Sci, 2021. **7**(4): p. 650-657.https://www.ncbi.nlm.nih.gov/pubmed/34056095
134. Zeng, Z., et al., *Increased expression of Siglec-9 in chronic obstructive pulmonary disease.* Sci Rep, 2017. **7**(1): p. 10116.https://www.ncbi.nlm.nih.gov/pubmed/28860481
135. Zheng, Y., et al., *The Roles of Siglec7 and Siglec9 on Natural Killer Cells in Virus Infection and Tumour Progression.* J Immunol Res, 2020. **2020**: p. 6243819.https://www.ncbi.nlm.nih.gov/pubmed/32322597
136. Borges, L., M. Kubin, and T. Kuhlman, *LIR9, an immunoglobulin-superfamily-activating receptor, is expressed as a transmembrane and as a secreted molecule.* Blood, 2003. **101**(4): p. 1484-6.https://www.ncbi.nlm.nih.gov/pubmed/12393390
137. Lewis Marffy, A.L. and A.J. McCarthy, *Leukocyte Immunoglobulin-Like Receptors (LILRs) on Human Neutrophils: Modulators of Infection and Immunity.* Front Immunol, 2020. **11**: p. 857.https://www.ncbi.nlm.nih.gov/pubmed/32477348

138. Li, M., et al., *Elevated Exhaustion Levels of NK and CD8(+) T Cells as Indicators for Progression and Prognosis of COVID-19 Disease.* Front Immunol, 2020. **11**: p. 580237.https://www.ncbi.nlm.nih.gov/pubmed/33154753
139. Mazzoni, A., et al., *Impaired immune cell cytotoxicity in severe COVID-19 is IL-6 dependent.* J Clin Invest, 2020. **130**(9): p. 4694-4703.https://www.ncbi.nlm.nih.gov/pubmed/32463803
140. Leal Rojas, I.M., et al., *Human Blood CD1c(+) Dendritic Cells Promote Th1 and Th17 Effector Function in Memory CD4(+) T Cells.* Front Immunol, 2017. **8**: p. 971.https://www.ncbi.nlm.nih.gov/pubmed/28878767
141. Porcelli, S., et al., *Recognition of cluster of differentiation 1 antigens by human CD4-CD8-cytolytic T lymphocytes.* Nature, 1989. **341**(6241): p. 447-50.https://www.ncbi.nlm.nih.gov/pubmed/2477705
142. Langley, K.E., et al., *Soluble stem cell factor in human serum.* Blood, 1993. **81**(3): p. 656-60.https://www.ncbi.nlm.nih.gov/pubmed/7678995
143. Guimond, M., et al., *In vivo role of Flt3 ligand and dendritic cells in NK cell homeostasis.* J Immunol, 2010. **184**(6): p. 2769-75.https://www.ncbi.nlm.nih.gov/pubmed/20142363
144. Padilla, O., et al., *Cloning of S4D-SRCRB, a new soluble member of the group B scavenger receptor cysteine-rich family (SRCR-SF) mapping to human chromosome 7q11.23.* Immunogenetics, 2002. **54**(9): p. 621-34.https://www.ncbi.nlm.nih.gov/pubmed/12466895
145. Lee, J.S., et al., *Longitudinal proteomic profiling provides insights into host response and proteome dynamics in COVID-19 progression.* Proteomics, 2021. **21**(11-12): p. e2000278.https://www.ncbi.nlm.nih.gov/pubmed/33945677
146. Consortium, C.-M.-o.B.A., et al., *A blood atlas of COVID-19 defines hallmarks of disease severity and specificity*. 2021, medRxiv.
147. Fang, X., et al., *Identification of Key Genes Associated with Changes in the Host Response to Severe Burn Shock: A Bioinformatics Analysis with Data from the Gene Expression Omnibus (GEO) Database.* J Inflamm Res, 2020. **13**: p. 1029-1041.https://www.ncbi.nlm.nih.gov/pubmed/33293847
148. Persson, T., et al., *Expression of the neutrophil-activating CXC chemokine ENA-78/CXCL5 by human eosinophils.* Clin Exp Allergy, 2003. **33**(4): p. 531-7.https://www.ncbi.nlm.nih.gov/pubmed/12680872
149. Liang, Y., et al., *Role of neutrophil chemoattractant CXCL5 in SARS-CoV-2 infection-induced lung inflammatory innate immune response in an in vivo hACE2 transfection mouse model.* Zool Res, 2020. **41**(6): p. 621-631.https://www.ncbi.nlm.nih.gov/pubmed/33045777
150. Ratthe, C. and D. Girard, *Interleukin-15 enhances human neutrophil phagocytosis by a Syk-dependent mechanism: importance of the IL-15Ralpha chain.* J Leukoc Biol, 2004. **76**(1): p. 162-8.https://www.ncbi.nlm.nih.gov/pubmed/15123770
151. Verri, W.A., Jr., et al., *IL-15 mediates antigen-induced neutrophil migration by triggering IL-18 production.* Eur J Immunol, 2007. **37**(12): p. 3373-80.https://www.ncbi.nlm.nih.gov/pubmed/17979156
152. Waickman, A.T., et al., *CD4 effector T cell differentiation is controlled by IL-15 that is expressed and presented in trans.* Cytokine, 2017. **99**: p. 266-274.https://www.ncbi.nlm.nih.gov/pubmed/28807496
153. Lo Iacono, O., et al., *Soluble adhesion molecules correlate with liver inflammation and fibrosis in chronic hepatitis C treated with interferon-alpha.* Aliment Pharmacol Ther, 1998. **12**(11): p. 1091-9.https://www.ncbi.nlm.nih.gov/pubmed/9845398
154. Tchalla, A., et al., *High levels of an endothelial dysfunction marker (sVCAM-1) are associated with injurious and recurrent falls and mortality over a 5-year interval in an older population.* Exp Gerontol, 2018. **106**: p. 1-7.https://www.ncbi.nlm.nih.gov/pubmed/29481968
155. Choudhury, A., et al., *Platelet surface CD62P and CD63, mean platelet volume, and soluble/platelet P-selectin as indexes of platelet function in atrial fibrillation: a comparison of "healthy control subjects" and "disease control subjects" in sinus rhythm.* J Am Coll Cardiol, 2007. **49**(19): p. 1957-64.https://www.ncbi.nlm.nih.gov/pubmed/17498581
156. Kallquist, L., et al., *The tetraspanin CD63 is involved in granule targeting of neutrophil elastase.* Blood, 2008. **112**(8): p. 3444-54.https://www.ncbi.nlm.nih.gov/pubmed/18669870

157. Heissig, B., et al., *Role of neutrophil-derived matrix metalloproteinase-9 in tissue regeneration.* Histol Histopathol, 2010. **25**(6): p. 765-70.https://www.ncbi.nlm.nih.gov/pubmed/20376783
158. Ueland, T., et al., *Distinct and early increase in circulating MMP-9 in COVID-19 patients with respiratory failure.* J Infect, 2020. **81**(3): p. e41-e43.https://www.ncbi.nlm.nih.gov/pubmed/32603675
159. Hu, F., et al., *Scavenger receptor-A is a biomarker and effector of rheumatoid arthritis: A large-scale multicenter study.* Nat Commun, 2020. **11**(1): p. 1911.https://www.ncbi.nlm.nih.gov/pubmed/32312978
160. Tang, Y., et al., *Macrophage scavenger receptor 1 contributes to pathogenesis of fulminant hepatitis via neutrophil-mediated complement activation.* J Hepatol, 2018. **68**(4): p. 733-743.https://www.ncbi.nlm.nih.gov/pubmed/29154963
161. Jedynak, M., et al., *Soluble TREM-1 Serum Level can Early Predict Mortality of Patients with Sepsis, Severe Sepsis and Septic Shock.* Arch Immunol Ther Exp (Warsz), 2018. **66**(4): p. 299-306.https://www.ncbi.nlm.nih.gov/pubmed/29282483
162. Mahdy, A.M., et al., *Production of soluble triggering receptor expressed on myeloid cells by lipopolysaccharide-stimulated human neutrophils involves de novo protein synthesis.* Clin Vaccine Immunol, 2006. **13**(4): p. 492-5.https://www.ncbi.nlm.nih.gov/pubmed/16603617
163. Lech, M., et al., *Circulating Markers of Inflammation Persist in Children and Adults With Giant Aneurysms After Kawasaki Disease.* Circ Genom Precis Med, 2019. **12**(4): p. e002433.https://www.ncbi.nlm.nih.gov/pubmed/30844302
164. Stegemann, C., et al., *Proteomic identification of matrix metalloproteinase substrates in the human vasculature.* Circ Cardiovasc Genet, 2013. **6**(1): p. 106-17.https://www.ncbi.nlm.nih.gov/pubmed/23255316
165. Arunachalam, P.S., et al., *Systems biological assessment of immunity to mild versus severe COVID-19 infection in humans.* Science, 2020. **369**(6508): p. 1210-1220.https://www.ncbi.nlm.nih.gov/pubmed/32788292
166. Ayi, K., et al., *CD47-SIRPalpha Interactions Regulate Macrophage Uptake of Plasmodium falciparum-Infected Erythrocytes and Clearance of Malaria In Vivo.* Infect Immun, 2016. **84**(7): p. 2002-2011.https://www.ncbi.nlm.nih.gov/pubmed/27091932
167. Korkmaz, B., et al., *Therapeutic targeting of cathepsin C: from pathophysiology to treatment.* Pharmacol Ther, 2018. **190**: p. 202-236.https://www.ncbi.nlm.nih.gov/pubmed/29842917
168. Xiao, Y., et al., *Cathepsin C promotes breast cancer lung metastasis by modulating neutrophil infiltration and neutrophil extracellular trap formation.* Cancer Cell, 2021. **39**(3): p. 423-437 e7.https://www.ncbi.nlm.nih.gov/pubmed/33450198
169. He, C.H., et al., *Chitinase 3-like 1 regulates cellular and tissue responses via IL-13 receptor alpha2.* Cell Rep, 2013. **4**(4): p. 830-41.https://www.ncbi.nlm.nih.gov/pubmed/23972995
170. Zhao, T., et al., *Chitinase-3 like-protein-1 function and its role in diseases.* Signal Transduct Target Ther, 2020. **5**(1): p. 201.https://www.ncbi.nlm.nih.gov/pubmed/32929074
171. Coriati, A., et al., *Neutrophils as a Potential Source of Chitinase-3-like Protein 1 in Cystic Fibrosis.* Inflammation, 2018. **41**(5): p. 1631-1639.https://www.ncbi.nlm.nih.gov/pubmed/29804188
172. Deutschmann, C., et al., *Identification of Chitinase-3-Like Protein 1 as a Novel Neutrophil Antigenic Target in Crohn's Disease.* J Crohns Colitis, 2019. **13**(7): p. 894-904.https://www.ncbi.nlm.nih.gov/pubmed/30753386
173. Demetri, G.D. and J.D. Griffin, *Granulocyte colony-stimulating factor and its receptor.* Blood, 1991. **78**(11): p. 2791-808.https://www.ncbi.nlm.nih.gov/pubmed/1720034
174. Chen, Q., et al., *Increased gene copy number of DEFA1/DEFA3 worsens sepsis by inducing endothelial pyroptosis.* Proc Natl Acad Sci U S A, 2019. **116**(8): p. 3161-3170.https://www.ncbi.nlm.nih.gov/pubmed/30718392
175. Maneerat, Y., et al., *Increased alpha-defensin expression is associated with risk of coronary heart disease: a feasible predictive inflammatory biomarker of coronary heart disease in hyperlipidemia patients.* Lipids Health Dis, 2016. **15**: p. 117.https://www.ncbi.nlm.nih.gov/pubmed/27430968

176. Schroll, A., et al., *Lipocalin-2 ameliorates granulocyte functionality.* Eur J Immunol, 2012. **42**(12): p. 3346-57.https://www.ncbi.nlm.nih.gov/pubmed/22965758
177. Chun, H.J., et al., *Immunofibrotic drivers of impaired lung function in postacute sequelae of SARS-CoV-2 infection.* JCI Insight, 2021. **6**(14).https://www.ncbi.nlm.nih.gov/pubmed/34111030
178. Senior, R.M., et al., *Entactin stimulates neutrophil adhesion and chemotaxis through interactions between its Arg-Gly-Asp (RGD) domain and the leukocyte response integrin.* J Clin Invest, 1992. **90**(6): p. 2251-7.https://www.ncbi.nlm.nih.gov/pubmed/1469085
179. Kobuch, J., et al., *TIMP-1 signaling via CD63 triggers granulopoiesis and neutrophilia in mice.* Haematologica, 2015. **100**(8): p. 1005-13.https://www.ncbi.nlm.nih.gov/pubmed/26001794
180. Yang, J., et al., *Impact of Kidney Function on the Blood Proteome and on Protein Cardiovascular Risk Biomarkers in Patients With Stable Coronary Heart Disease.* J Am Heart Assoc, 2020. **9**(15): p. e016463.https://www.ncbi.nlm.nih.gov/pubmed/32696702
181. Mackman, N., *Role of tissue factor in hemostasis, thrombosis, and vascular development.* Arterioscler Thromb Vasc Biol, 2004. **24**(6): p. 1015-22.https://www.ncbi.nlm.nih.gov/pubmed/15117736
182. Egorina, E.M., M.A. Sovershaev, and J.B. Hansen, *The role of tissue factor in systemic inflammatory response syndrome.* Blood Coagul Fibrinolysis, 2011. **22**(6): p. 451-6.https://www.ncbi.nlm.nih.gov/pubmed/21597365
183. Campo, G., et al., *Tissue factor and coagulation factor VII levels during acute myocardial infarction: association with genotype and adverse events.* Arterioscler Thromb Vasc Biol, 2006. **26**(12): p. 2800-6.https://www.ncbi.nlm.nih.gov/pubmed/17008590
184. Suefuji, H., et al., *Increased plasma tissue factor levels in acute myocardial infarction.* Am Heart J, 1997. **134**(2 Pt 1): p. 253-9.https://www.ncbi.nlm.nih.gov/pubmed/9313605
185. Rezaie, A.R., *Regulation of the protein C anticoagulant and antiinflammatory pathways.* Curr Med Chem, 2010. **17**(19): p. 2059-69.https://www.ncbi.nlm.nih.gov/pubmed/20423310
186. Laskowska, M., K. Laskowska, and J. Oleszczuk, *The relation of maternal serum eNOS, NOSTRIN and ADMA levels with aetiopathogenesis of preeclampsia and/or intrauterine fetal growth restriction.* J Matern Fetal Neonatal Med, 2015. **28**(1): p. 26-32.https://www.ncbi.nlm.nih.gov/pubmed/24588201
187. Tasolar, H., et al., *Endothelial nitric oxide synthase levels and their response to exercise in patients with slow coronary flow.* Cardiovasc J Afr, 2013. **24**(9-10): p. 355-9.https://www.ncbi.nlm.nih.gov/pubmed/24337211
188. Guimaraes, L.M.F., C.V.T. Rossini, and C. Lameu, *Implications of SARS-Cov-2 infection on eNOS and iNOS activity: Consequences for the respiratory and vascular systems.* Nitric Oxide, 2021. **111-112**: p. 64-71.https://www.ncbi.nlm.nih.gov/pubmed/33831567
189. Rabelink, T.J. and A.J. van Zonneveld, *Coupling eNOS uncoupling to the innate immune response.* Arterioscler Thromb Vasc Biol, 2006. **26**(12): p. 2585-7.https://www.ncbi.nlm.nih.gov/pubmed/17110608
190. Islam, S., et al., *Serum levels of thrombotic markers in patients with acute myocardial infarction.* Int J Clin Exp Med, 2014. **7**(4): p. 1059-63.https://www.ncbi.nlm.nih.gov/pubmed/24955182
191. Thogersen, A.M., et al., *High plasminogen activator inhibitor and tissue plasminogen activator levels in plasma precede a first acute myocardial infarction in both men and women: evidence for the fibrinolytic system as an independent primary risk factor.* Circulation, 1998. **98**(21): p. 2241-7.https://www.ncbi.nlm.nih.gov/pubmed/9826309
192. White, D., et al., *Evaluation of COVID-19 coagulopathy; laboratory characterization using thrombin generation and nonconventional haemostasis assays.* Int J Lab Hematol, 2021. **43**(1): p. 123-130.https://www.ncbi.nlm.nih.gov/pubmed/32892505
193. Zhang, B., R.J. Kaufman, and D. Ginsburg, *LMAN1 and MCFD2 form a cargo receptor complex and interact with coagulation factor VIII in the early secretory pathway.* J Biol Chem, 2005. **280**(27): p. 25881-6.https://www.ncbi.nlm.nih.gov/pubmed/15886209

194. Toda, H., et al., *Stem cell-derived neural stem/progenitor cell supporting factor is an autocrine/paracrine survival factor for adult neural stem/progenitor cells.* J Biol Chem, 2003. **278**(37): p. 35491-500.https://www.ncbi.nlm.nih.gov/pubmed/12832409
195. Coll, B., et al., *Serum levels of matrix metalloproteinase-10 are associated with the severity of atherosclerosis in patients with chronic kidney disease.* Kidney Int, 2010. **78**(12): p. 1275-80.https://www.ncbi.nlm.nih.gov/pubmed/20844474
196. Martinez-Aguilar, E., et al., *Matrix metalloproteinase 10 is associated with disease severity and mortality in patients with peripheral arterial disease.* J Vasc Surg, 2015. **61**(2): p. 428-35.https://www.ncbi.nlm.nih.gov/pubmed/25441671
197. Rodriguez, J.A., et al., *Metalloproteinases and atherothrombosis: MMP-10 mediates vascular remodeling promoted by inflammatory stimuli.* Front Biosci, 2008. **13**: p. 2916-21.https://www.ncbi.nlm.nih.gov/pubmed/17981764
198. Shi, S., et al., *Matrix metalloproteinase 3 as a valuable marker for patients with COVID-19.* J Med Virol, 2021. **93**(1): p. 528-532.https://www.ncbi.nlm.nih.gov/pubmed/32603484
199. Wijten, P., et al., *High precision platelet releasate definition by quantitative reversed protein profiling--brief report.* Arterioscler Thromb Vasc Biol, 2013. **33**(7): p. 1635-8.https://www.ncbi.nlm.nih.gov/pubmed/23640497
200. Rosas, I.O., et al., *MMP1 and MMP7 as potential peripheral blood biomarkers in idiopathic pulmonary fibrosis.* PLoS Med, 2008. **5**(4): p. e93.https://www.ncbi.nlm.nih.gov/pubmed/18447576
201. Tzouvelekis, A., et al., *Validation of the prognostic value of MMP-7 in idiopathic pulmonary fibrosis.* Respirology, 2017. **22**(3): p. 486-493.https://www.ncbi.nlm.nih.gov/pubmed/27761978
202. Ling, N.R., et al., *Origin and properties of soluble CD21 (CR2) in human blood.* Clin Exp Immunol, 1998. **113**(3): p. 360-6.https://www.ncbi.nlm.nih.gov/pubmed/9737663
203. Barrington, R.A., et al., *Uncoupling CD21 and CD19 of the B-cell coreceptor.* Proc Natl Acad Sci U S A, 2009. **106**(34): p. 14490-5.https://www.ncbi.nlm.nih.gov/pubmed/19706534
204. Foley, J.H. and E.M. Conway, *Cross Talk Pathways Between Coagulation and Inflammation.* Circ Res, 2016. **118**(9): p. 1392-408.https://www.ncbi.nlm.nih.gov/pubmed/27126649
205. Ludeman, M.J., et al., *Regulated shedding of PAR1 N-terminal exodomain from endothelial cells.* J Biol Chem, 2004. **279**(18): p. 18592-9.https://www.ncbi.nlm.nih.gov/pubmed/14982936
206. Yu, L., et al., *Prognostic significance of urokinase-type plasminogen activator and its receptor in patients with systemic inflammatory response syndrome.* World J Emerg Med, 2011. **2**(3): p. 185-9.https://www.ncbi.nlm.nih.gov/pubmed/25215007
207. van Hinsbergh, V.W., et al., *Tumor necrosis factor induces the production of urokinase-type plasminogen activator by human endothelial cells.* Blood, 1990. **75**(10): p. 1991-8.https://www.ncbi.nlm.nih.gov/pubmed/2140060
208. Wojta, J., et al., *Interferon-alpha 2 counteracts interleukin-1 alpha-stimulated expression of urokinase-type plasminogen activator in human foreskin microvascular endothelial cells in vitro.* Lymphokine Cytokine Res, 1994. **13**(2): p. 133-8.https://www.ncbi.nlm.nih.gov/pubmed/8061114
209. Goshua, G., et al., *Endotheliopathy in COVID-19-associated coagulopathy: evidence from a single-centre, cross-sectional study.* Lancet Haematol, 2020. **7**(8): p. e575-e582.https://www.ncbi.nlm.nih.gov/pubmed/32619411
210. Califano, F., et al., *Clinical importance of thrombomodulin serum levels.* Eur Rev Med Pharmacol Sci, 2000. **4**(3): p. 59-66.https://www.ncbi.nlm.nih.gov/pubmed/11558626
211. Goicoechea de Jorge, E., et al., *Dimerization of complement factor H-related proteins modulates complement activation in vivo.* Proc Natl Acad Sci U S A, 2013. **110**(12): p. 4685-90.https://www.ncbi.nlm.nih.gov/pubmed/23487775
212. Bellac, C.L., et al., *Macrophage matrix metalloproteinase-12 dampens inflammation and neutrophil influx in arthritis.* Cell Rep, 2014. **9**(2): p. 618-32.https://www.ncbi.nlm.nih.gov/pubmed/25310974

213. Rovina, N., et al., *Soluble urokinase plasminogen activator receptor (suPAR) as an early predictor of severe respiratory failure in patients with COVID-19 pneumonia.* Crit Care, 2020. **24**(1): p. 187.https://www.ncbi.nlm.nih.gov/pubmed/32354367
214. Huang, M., et al., *Plasma levels of the active form of suPAR are associated with COVID-19 severity.* Crit Care, 2020. **24**(1): p. 704.https://www.ncbi.nlm.nih.gov/pubmed/33372603
215. Radomski, A., et al., *Identification, regulation and role of tissue inhibitor of metalloproteinases-4 (TIMP-4) in human platelets.* Br J Pharmacol, 2002. **137**(8): p. 1330-8.https://www.ncbi.nlm.nih.gov/pubmed/12466243
216. Ruggeri, Z.M. and G.L. Mendolicchio, *Adhesion mechanisms in platelet function.* Circ Res, 2007. **100**(12): p. 1673-85.https://www.ncbi.nlm.nih.gov/pubmed/17585075
217. Garlanda, C., et al., *Pentraxins at the crossroads between innate immunity, inflammation, matrix deposition, and female fertility.* Annu Rev Immunol, 2005. **23**: p. 337-66.https://www.ncbi.nlm.nih.gov/pubmed/15771574
218. Latini, R., et al., *Prognostic significance of the long pentraxin PTX3 in acute myocardial infarction.* Circulation, 2004. **110**(16): p. 2349-54.https://www.ncbi.nlm.nih.gov/pubmed/15477419
219. Ma, Y.J. and P. Garred, *Pentraxins in Complement Activation and Regulation.* Front Immunol, 2018. **9**: p. 3046.https://www.ncbi.nlm.nih.gov/pubmed/30619374
220. Muller, B., et al., *Circulating levels of the long pentraxin PTX3 correlate with severity of infection in critically ill patients.* Crit Care Med, 2001. **29**(7): p. 1404-7.https://www.ncbi.nlm.nih.gov/pubmed/11445697
221. Zheng, P.S., et al., *Versican G3 domain promotes blood coagulation through suppressing the activity of tissue factor pathway inhibitor-1.* J Biol Chem, 2006. **281**(12): p. 8175-82.https://www.ncbi.nlm.nih.gov/pubmed/16431924
222. Awwad, K., et al., *Role of secreted modular calcium-binding protein 1 (SMOC1) in transforming growth factor beta signalling and angiogenesis.* Cardiovasc Res, 2015. **106**(2): p. 284-94.https://www.ncbi.nlm.nih.gov/pubmed/25750188
223. Delgado Lagos, F., et al., *Secreted modular calcium-binding protein 1 binds and activates thrombin to account for platelet hyperreactivity in diabetes.* Blood, 2021. **137**(12): p. 1641-1651.https://www.ncbi.nlm.nih.gov/pubmed/33529332
224. Owens, A.W., et al., *Circulating lymphotoxin beta receptor and atherosclerosis: observations from the Dallas Heart Study.* Atherosclerosis, 2010. **212**(2): p. 601-6.https://www.ncbi.nlm.nih.gov/pubmed/20599198
225. DeBerge, M.P., et al., *Shedding of TNF receptor 2 by effector CD8(+) T cells by ADAM17 is important for regulating TNF-alpha availability during influenza infection.* J Leukoc Biol, 2015. **98**(3): p. 423-34.https://www.ncbi.nlm.nih.gov/pubmed/26019295
226. Porteu, F., et al., *Human neutrophil elastase releases a ligand-binding fragment from the 75-kDa tumor necrosis factor (TNF) receptor. Comparison with the proteolytic activity responsible for shedding of TNF receptors from stimulated neutrophils.* J Biol Chem, 1991. **266**(28): p. 18846-53.https://www.ncbi.nlm.nih.gov/pubmed/1655765
227. Porteu, F. and C. Nathan, *Shedding of tumor necrosis factor receptors by activated human neutrophils.* J Exp Med, 1990. **172**(2): p. 599-607.https://www.ncbi.nlm.nih.gov/pubmed/2165128
228. Carlsson, A.C., et al., *Association of soluble tumor necrosis factor receptors 1 and 2 with nephropathy, cardiovascular events, and total mortality in type 2 diabetes.* Cardiovasc Diabetol, 2016. **15**: p. 40.https://www.ncbi.nlm.nih.gov/pubmed/26928194
229. Neirynck, N., et al., *Soluble tumor necrosis factor receptor 1 and 2 predict outcomes in advanced chronic kidney disease: a prospective cohort study.* PLoS One, 2015. **10**(3): p. e0122073.https://www.ncbi.nlm.nih.gov/pubmed/25823004
230. Tziakas, D., et al., *Prolonged activation of tumor necrosis factor (TNF)-alpha and its soluble receptors in chronic heart failure patients both in the compensated and decompensated state. Interplay between*

their levels and metalloproteinase-3. Eur Cytokine Netw, 2004. **15**(3): p. 231-9.https://www.ncbi.nlm.nih.gov/pubmed/15542448
231. Kong, Y., et al., *Storm of soluble immune checkpoints associated with disease severity of COVID-19.* Signal Transduct Target Ther, 2020. **5**(1): p. 192.https://www.ncbi.nlm.nih.gov/pubmed/32895366
232. He, Q., et al., *Correlation of osteoprotegerin, sRANKL, inflammatory factors and epicardial adipose tissue volume with coronary heart disease.* Int J Clin Pract, 2021. **75**(7): p. e14207.https://www.ncbi.nlm.nih.gov/pubmed/33813793
233. Schoppet, M., J.R. Schaefer, and L.C. Hofbauer, *Low serum levels of soluble RANK ligand are associated with the presence of coronary artery disease in men.* Circulation, 2003. **107**(11): p. e76; author reply e76.https://www.ncbi.nlm.nih.gov/pubmed/12654623
234. Levine, S.J., *Molecular mechanisms of soluble cytokine receptor generation.* J Biol Chem, 2008. **283**(21): p. 14177-81.https://www.ncbi.nlm.nih.gov/pubmed/18385130
235. Akiyama, T., M. Shinzawa, and N. Akiyama, *RANKL-RANK interaction in immune regulatory systems.* World J Orthop, 2012. **3**(9): p. 142-50.https://www.ncbi.nlm.nih.gov/pubmed/23173110
236. Lacy, M., et al., *Cell-specific and divergent roles of the CD40L-CD40 axis in atherosclerotic vascular disease.* Nat Commun, 2021. **12**(1): p. 3754.https://www.ncbi.nlm.nih.gov/pubmed/34145241
237. Goncalves, I., et al., *sTRAIL-R2 (Soluble TNF [Tumor Necrosis Factor]-Related Apoptosis-Inducing Ligand Receptor 2) a Marker of Plaque Cell Apoptosis and Cardiovascular Events.* Stroke, 2019. **50**(8): p. 1989-1996.https://www.ncbi.nlm.nih.gov/pubmed/31272321
238. Mielczarek-Palacz, A., Z. Kondera-Anasz, and M. Smycz-Kubanska, *Changes in the Concentration of Markers Participating in the Regulation of the Apoptosis Receptor Pathway Involving Soluble Tumour Necrosis Factor Ligand inducing Apoptosis (sTRAIL) and Osteoprotegerin (OPG) in the Serum of Women with Ovarian Cancer-Participation in Pathogenesis or a Possible Clinical Use?* Cells, 2020. **9**(3).https://www.ncbi.nlm.nih.gov/pubmed/32143328
239. Mielczarek-Palacz, A., J. Sikora, and Z. Kondera-Anasz, *Assessment of concentrations of sTRAIL ligand and its receptors sTRAIL-R1 and sTRAIL-R2 - markers monitoring the course of the extrinsic pathway of apoptosis induction: potential application in ovarian cancer diagnostics.* Arch Med Sci, 2017. **13**(3): p. 624-628.https://www.ncbi.nlm.nih.gov/pubmed/28507579
240. Karadag, D.T., et al., *TNF-Related Apoptosis-Inducing Ligand Receptor 1 in Patients With Ankylosing Spondylitis.* J Clin Rheumatol, 2020. **26**(6): p. 242-247.https://www.ncbi.nlm.nih.gov/pubmed/31094932
241. Lynch, C.N., et al., *TWEAK induces angiogenesis and proliferation of endothelial cells.* J Biol Chem, 1999. **274**(13): p. 8455-9.https://www.ncbi.nlm.nih.gov/pubmed/10085077
242. Campbell, S., et al., *The role of TWEAK/Fn14 in the pathogenesis of inflammation and systemic autoimmunity.* Front Biosci, 2004. **9**: p. 2273-84.https://www.ncbi.nlm.nih.gov/pubmed/15353286
243. Burkly, L.C., et al., *TWEAKing tissue remodeling by a multifunctional cytokine: role of TWEAK/Fn14 pathway in health and disease.* Cytokine, 2007. **40**(1): p. 1-16.https://www.ncbi.nlm.nih.gov/pubmed/17981048
244. Sharif, M.N., et al., *Soluble Fn14 Is Detected and Elevated in Mouse and Human Kidney Disease.* PLoS One, 2016. **11**(5): p. e0155368.https://www.ncbi.nlm.nih.gov/pubmed/27171494
245. Figgett, W.A., et al., *The TACI receptor regulates T-cell-independent marginal zone B cell responses through innate activation-induced cell death.* Immunity, 2013. **39**(3): p. 573-83.https://www.ncbi.nlm.nih.gov/pubmed/24012421
246. von Bulow, G.U., J.M. van Deursen, and R.J. Bram, *Regulation of the T-independent humoral response by TACI.* Immunity, 2001. **14**(5): p. 573-82.https://www.ncbi.nlm.nih.gov/pubmed/11371359
247. Hoffmann, F.S., et al., *The immunoregulator soluble TACI is released by ADAM10 and reflects B cell activation in autoimmunity.* J Immunol, 2015. **194**(2): p. 542-52.https://www.ncbi.nlm.nih.gov/pubmed/25505277

248. Michel, J., et al., *A soluble form of CD137 (ILA/4-1BB), a member of the TNF receptor family, is released by activated lymphocytes and is detectable in sera of patients with rheumatoid arthritis.* Eur J Immunol, 1998. **28**(1): p. 290-5.https://www.ncbi.nlm.nih.gov/pubmed/9485208
249. Furtner, M., et al., *Levels of soluble CD137 are enhanced in sera of leukemia and lymphoma patients and are strongly associated with chronic lymphocytic leukemia.* Leukemia, 2005. **19**(5): p. 883-5.https://www.ncbi.nlm.nih.gov/pubmed/15744355
250. Luu, K., Z. Shao, and H. Schwarz, *The relevance of soluble CD137 in the regulation of immune responses and for immunotherapeutic intervention.* J Leukoc Biol, 2020. **107**(5): p. 731-738.https://www.ncbi.nlm.nih.gov/pubmed/32052477
251. He, Y., et al., *Increased Soluble CD137 Levels and CD4+ T-Cell-Associated Expression of CD137 in Acute Atherothrombotic Stroke.* Clin Transl Sci, 2018. **11**(4): p. 428-434.https://www.ncbi.nlm.nih.gov/pubmed/29697202
252. Tanikawa, C., et al., *Crosstalk of EDA-A2/XEDAR in the p53 signaling pathway.* Mol Cancer Res, 2010. **8**(6): p. 855-63.https://www.ncbi.nlm.nih.gov/pubmed/20501644
253. Cascino, I., et al., *Three functional soluble forms of the human apoptosis-inducing Fas molecule are produced by alternative splicing.* J Immunol, 1995. **154**(6): p. 2706-13.https://www.ncbi.nlm.nih.gov/pubmed/7533181
254. Tortorella, C., et al., *sICAM-1, sCD95 and sCD95L levels in chronic liver diseases of different etiology.* Immunopharmacol Immunotoxicol, 2000. **22**(1): p. 19-33.https://www.ncbi.nlm.nih.gov/pubmed/10737254
255. Bhatraju, P.K., et al., *Circulating levels of soluble Fas (sCD95) are associated with risk for development of a nonresolving acute kidney injury subphenotype.* Crit Care, 2017. **21**(1): p. 217.https://www.ncbi.nlm.nih.gov/pubmed/28814331
256. Ankersmit, H.J., et al., *Increased serum concentrations of soluble CD95/Fas and caspase 1/ICE in patients with acute angina.* Heart, 2004. **90**(2): p. 151-4.https://www.ncbi.nlm.nih.gov/pubmed/14729783
257. van der Voort, R., et al., *An alternatively spliced CXCL16 isoform expressed by dendritic cells is a secreted chemoattractant for CXCR6+ cells.* J Leukoc Biol, 2010. **87**(6): p. 1029-39.https://www.ncbi.nlm.nih.gov/pubmed/20181724
258. Havlioglu, N., et al., *Slit proteins, potential endogenous modulators of inflammation.* J Neurovirol, 2002. **8**(6): p. 486-95.https://www.ncbi.nlm.nih.gov/pubmed/12476344
259. Garcia-Zepeda, E.A., et al., *Human eotaxin is a specific chemoattractant for eosinophil cells and provides a new mechanism to explain tissue eosinophilia.* Nat Med, 1996. **2**(4): p. 449-56.https://www.ncbi.nlm.nih.gov/pubmed/8597956
260. Sa, V.C., et al., *The pattern of immune cell infiltration in chromoblastomycosis: involvement of macrophage inflammatory protein-1 alpha/CCL3 and fungi persistence.* Rev Inst Med Trop Sao Paulo, 2007. **49**(1): p. 49-53.https://www.ncbi.nlm.nih.gov/pubmed/17384820
261. Imai, T., et al., *Identification and molecular characterization of fractalkine receptor CX3CR1, which mediates both leukocyte migration and adhesion.* Cell, 1997. **91**(4): p. 521-30.https://www.ncbi.nlm.nih.gov/pubmed/9390561
262. Sancho, D. and C. Reis e Sousa, *Signaling by myeloid C-type lectin receptors in immunity and homeostasis.* Annu Rev Immunol, 2012. **30**: p. 491-529.https://www.ncbi.nlm.nih.gov/pubmed/22224766
263. Sung, P.S. and S.L. Hsieh, *C-type lectins and extracellular vesicles in virus-induced NETosis.* J Biomed Sci, 2021. **28**(1): p. 46.https://www.ncbi.nlm.nih.gov/pubmed/34116654
264. Richardson, M.B. and S.J. Williams, *MCL and Mincle: C-Type Lectin Receptors That Sense Damaged Self and Pathogen-Associated Molecular Patterns.* Front Immunol, 2014. **5**: p. 288.https://www.ncbi.nlm.nih.gov/pubmed/25002863

265. Lopez Robles, M.D., et al., *Cell-surface C-type lectin-like receptor CLEC-1 dampens dendritic cell activation and downstream Th17 responses.* Blood Adv, 2017. **1**(9): p. 557-568.https://www.ncbi.nlm.nih.gov/pubmed/29296975
266. Sattler, S., et al., *The human C-type lectin-like receptor CLEC-1 is upregulated by TGF-beta and primarily localized in the endoplasmic membrane compartment.* Scand J Immunol, 2012. **75**(3): p. 282-92.https://www.ncbi.nlm.nih.gov/pubmed/22117783
267. Thebault, P., et al., *The C-type lectin-like receptor CLEC-1, expressed by myeloid cells and endothelial cells, is up-regulated by immunoregulatory mediators and moderates T cell activation.* J Immunol, 2009. **183**(5): p. 3099-108.https://www.ncbi.nlm.nih.gov/pubmed/19667084
268. Takahashi, Y., et al., *Histidine-Rich Glycoprotein Stimulates Human Neutrophil Phagocytosis and Prolongs Survival through CLEC1A.* J Immunol, 2021. **206**(4): p. 737-750.https://www.ncbi.nlm.nih.gov/pubmed/33452125
269. Schwingshackl, P., et al., *Distribution and maturation of skin dendritic cell subsets in two forms of cutaneous T-cell lymphoma: mycosis fungoides and Sezary syndrome.* Acta Derm Venereol, 2012. **92**(3): p. 269-75.https://www.ncbi.nlm.nih.gov/pubmed/22678564
270. Soldevila, G., C. Raman, and F. Lozano, *The immunomodulatory properties of the CD5 lymphocyte receptor in health and disease.* Curr Opin Immunol, 2011. **23**(3): p. 310-8.https://www.ncbi.nlm.nih.gov/pubmed/21482089
271. Aibar, J., et al., *Pattern of soluble CD5 and CD6 lymphocyte receptors in critically ill patients with septic syndromes.* J Crit Care, 2015. **30**(5): p. 914-9.https://www.ncbi.nlm.nih.gov/pubmed/26031813
272. Luster, A.D., J.C. Unkeless, and J.V. Ravetch, *Gamma-interferon transcriptionally regulates an early-response gene containing homology to platelet proteins.* Nature, 1985. **315**(6021): p. 672-6.https://www.ncbi.nlm.nih.gov/pubmed/3925348
273. Coperchini, F., L. Chiovato, and M. Rotondi, *Interleukin-6, CXCL10 and Infiltrating Macrophages in COVID-19-Related Cytokine Storm: Not One for All But All for One!* Front Immunol, 2021. **12**: p. 668507.https://www.ncbi.nlm.nih.gov/pubmed/33981314
274. Park, H.K., et al., *Linking resistin, inflammation, and cardiometabolic diseases.* Korean J Intern Med, 2017. **32**(2): p. 239-247.https://www.ncbi.nlm.nih.gov/pubmed/28192887
275. Tripathi, D., et al., *Resistin in metabolism, inflammation, and disease.* FEBS J, 2020. **287**(15): p. 3141-3149.https://www.ncbi.nlm.nih.gov/pubmed/32255270
276. Maurel, M., et al., *Control of anterior GRadient 2 (AGR2) dimerization links endoplasmic reticulum proteostasis to inflammation.* EMBO Mol Med, 2019. **11**(6).https://www.ncbi.nlm.nih.gov/pubmed/31040128
277. Tavernier, J., et al., *Molecular basis of the membrane-anchored and two soluble isoforms of the human interleukin 5 receptor alpha subunit.* Proc Natl Acad Sci U S A, 1992. **89**(15): p. 7041-5.https://www.ncbi.nlm.nih.gov/pubmed/1495999
278. Wilson, T.M., et al., *IL-5 receptor alpha levels in patients with marked eosinophilia or mastocytosis.* J Allergy Clin Immunol, 2011. **128**(5): p. 1086-92 e1-3.https://www.ncbi.nlm.nih.gov/pubmed/21762978
279. Takatsu, K., T. Kouro, and Y. Nagai, *Interleukin 5 in the link between the innate and acquired immune response.* Adv Immunol, 2009. **101**: p. 191-236.https://www.ncbi.nlm.nih.gov/pubmed/19231596
280. Linch, S.N., et al., *Interleukin 5 is protective during sepsis in an eosinophil-independent manner.* Am J Respir Crit Care Med, 2012. **186**(3): p. 246-54.https://www.ncbi.nlm.nih.gov/pubmed/22652030
281. Mazon, M., et al., *Elevated blood levels of Dickkopf-1 are associated with acute infections.* Immun Inflamm Dis, 2018. **6**(4): p. 428-434.https://www.ncbi.nlm.nih.gov/pubmed/30028084
282. Zhang, D.M., et al., *Cripto-1 modulates macrophage cytokine secretion and phagocytic activity via NF-kappaB signaling.* Immunol Res, 2016. **64**(1): p. 104-14.https://www.ncbi.nlm.nih.gov/pubmed/26476731
283. Klauzinska, M., et al., *Cripto-1: an extracellular protein - connecting the sequestered biological dots.* Connect Tissue Res, 2015. **56**(5): p. 364-80.https://www.ncbi.nlm.nih.gov/pubmed/26327334

284. Jenkins, R.W., et al., *Regulated secretion of acid sphingomyelinase: implications for selectivity of ceramide formation.* J Biol Chem, 2010. **285**(46): p. 35706-18.https://www.ncbi.nlm.nih.gov/pubmed/20807762
285. Chung, H.Y. and R.A. Claus, *Keep Your Friends Close, but Your Enemies Closer: Role of Acid Sphingomyelinase During Infection and Host Response.* Front Med (Lausanne), 2020. **7**: p. 616500.https://www.ncbi.nlm.nih.gov/pubmed/33553211
286. Wang, J., et al., *SARS-CoV-2 infection induces the activation of tissue factor-mediated coagulation via activation of acid sphingomyelinase.* Blood, 2021. **138**(4): p. 344-349.https://www.ncbi.nlm.nih.gov/pubmed/34075401
287. Beckmann, N. and K.A. Becker, *Ceramide and Related Molecules in Viral Infections.* Int J Mol Sci, 2021. **22**(11).https://www.ncbi.nlm.nih.gov/pubmed/34073578
288. Carpinteiro, A., et al., *Inhibition of acid sphingomyelinase by ambroxol prevents SARS-CoV-2 entry into epithelial cells.* J Biol Chem, 2021. **296**: p. 100701.https://www.ncbi.nlm.nih.gov/pubmed/33895135
289. Islam, A., et al., *Extracellular TNFR1 release requires the calcium-dependent formation of a nucleobindin 2-ARTS-1 complex.* J Biol Chem, 2006. **281**(10): p. 6860-73.https://www.ncbi.nlm.nih.gov/pubmed/16407280
290. Wang, Z.Z., et al., *Nesfatin-1 alleviates acute lung injury through reducing inflammation and oxidative stress via the regulation of HMGB1.* Eur Rev Med Pharmacol Sci, 2020. **24**(9): p. 5071-5081.https://www.ncbi.nlm.nih.gov/pubmed/32432771
291. Leivo-Korpela, S., et al., *Adipokines NUCB2/nesfatin-1 and visfatin as novel inflammatory factors in chronic obstructive pulmonary disease.* Mediators Inflamm, 2014. **2014**: p. 232167.https://www.ncbi.nlm.nih.gov/pubmed/24891763
292. Carmel, R. and D. Hollander, *Extreme elevation of transcobalamin II levels in multiple myeloma and other disorders.* Blood, 1978. **51**(6): p. 1057-63.https://www.ncbi.nlm.nih.gov/pubmed/647113
293. Jensen, H.S., et al., *Transcobalamin II as an indicator of activity in metastatic renal adenocarcinoma.* Cancer, 1983. **52**(9): p. 1700-4.https://www.ncbi.nlm.nih.gov/pubmed/6616421
294. Nawa, Y., et al., *Nucleophosmin may act as an alarmin: implications for severe sepsis.* J Leukoc Biol, 2009. **86**(3): p. 645-53.https://www.ncbi.nlm.nih.gov/pubmed/19581374
295. Hamilton, J.A., *Colony-stimulating factors in inflammation and autoimmunity.* Nat Rev Immunol, 2008. **8**(7): p. 533-44.https://www.ncbi.nlm.nih.gov/pubmed/18551128
296. Etzerodt, A. and S.K. Moestrup, *CD163 and inflammation: biological, diagnostic, and therapeutic aspects.* Antioxid Redox Signal, 2013. **18**(17): p. 2352-63.https://www.ncbi.nlm.nih.gov/pubmed/22900885
297. Zingaropoli, M.A., et al., *Increased sCD163 and sCD14 Plasmatic Levels and Depletion of Peripheral Blood Pro-Inflammatory Monocytes, Myeloid and Plasmacytoid Dendritic Cells in Patients With Severe COVID-19 Pneumonia.* Front Immunol, 2021. **12**: p. 627548.https://www.ncbi.nlm.nih.gov/pubmed/33777012
298. Kamarainen, M., et al., *RELP, a novel human REG-like protein with up-regulated expression in inflammatory and metaplastic gastrointestinal mucosa.* Am J Pathol, 2003. **163**(1): p. 11-20.https://www.ncbi.nlm.nih.gov/pubmed/12819006
299. Ma, X., et al., *The pancreatic cancer secreted REG4 promotes macrophage polarization to M2 through EGFR/AKT/CREB pathway.* Oncol Rep, 2016. **35**(1): p. 189-96.https://www.ncbi.nlm.nih.gov/pubmed/26531138
300. Lu, C., M.A. Amin, and D.A. Fox, *CD13/Aminopeptidase N Is a Potential Therapeutic Target for Inflammatory Disorders.* J Immunol, 2020. **204**(1): p. 3-11.https://www.ncbi.nlm.nih.gov/pubmed/31848300
301. Tsou E, et al. *Identification of CD13 as a Potential Cause for SARS-CoV-2-triggered Hyperinflammation and Thrombosis*. 2020 [cited Arthritis Rheumatol. 72 (suppl 10). Accessed October 5, 2021; Available from: https://acrabstracts.org/abstract/identification-of-cd13-as-a-potential-cause-for-sars-cov-2-triggered-hyperinflammation-and-thrombosis/.

302. Nezi, M., G. Mastorakos, and Z. Mouslech, *Corticotropin Releasing Hormone And The Immune/Inflammatory Response*, in *Endotext*, K.R. Feingold, et al., Editors. 2000: South Dartmouth (MA).
303. Li, W., et al., *STK4 regulates TLR pathways and protects against chronic inflammation-related hepatocellular carcinoma.* J Clin Invest, 2015. **125**(11): p. 4239-54.https://www.ncbi.nlm.nih.gov/pubmed/26457732
304. Potere, N., et al., *Low Density Lipoprotein Receptor-Related Protein-1 in Cardiac Inflammation and Infarct Healing.* Front Cardiovasc Med, 2019. **6**: p. 51.https://www.ncbi.nlm.nih.gov/pubmed/31080804
305. Gorovoy, M., et al., *Inflammatory mediators promote production of shed LRP1/CD91, which regulates cell signaling and cytokine expression by macrophages.* J Leukoc Biol, 2010. **88**(4): p. 769-78.https://www.ncbi.nlm.nih.gov/pubmed/20610799
306. Cattaneo, V., et al., *Galectin-8 elicits pro-inflammatory activities in the endothelium.* Glycobiology, 2014. **24**(10): p. 966-73.https://www.ncbi.nlm.nih.gov/pubmed/24957054
307. Cao, Z.Q. and X.L. Guo, *The role of galectin-4 in physiology and diseases.* Protein Cell, 2016. **7**(5): p. 314-24.https://www.ncbi.nlm.nih.gov/pubmed/27017379
308. Williams, D., et al., *Stable Flow-induced Expression of KLK10 Inhibits Endothelial Inflammation and Atherosclerosis.* bioRxiv, 2021: p. 2021.08.10.455857.https://www.biorxiv.org/content/biorxiv/early/2021/08/10/2021.08.10.455857.full.pdf
309. Iring, A., et al., *Shear stress-induced endothelial adrenomedullin signaling regulates vascular tone and blood pressure.* J Clin Invest, 2019. **129**(7): p. 2775-2791.https://www.ncbi.nlm.nih.gov/pubmed/31205027
310. Marino, R., et al., *Plasma adrenomedullin is associated with short-term mortality and vasopressor requirement in patients admitted with sepsis.* Crit Care, 2014. **18**(1): p. R34.https://www.ncbi.nlm.nih.gov/pubmed/24533868
311. Voors, A.A., et al., *Adrenomedullin in heart failure: pathophysiology and therapeutic application.* Eur J Heart Fail, 2019. **21**(2): p. 163-171.https://www.ncbi.nlm.nih.gov/pubmed/30592365
312. Marutsuka, K., et al., *Adrenomedullin augments the release and production of tissue factor pathway inhibitor in human aortic endothelial cells.* Cardiovasc Res, 2003. **57**(1): p. 232-7.https://www.ncbi.nlm.nih.gov/pubmed/12504833
313. Gregoriano, C., et al., *The vasoactive peptide MR-pro-adrenomedullin in COVID-19 patients: an observational study.* Clin Chem Lab Med, 2021. **59**(5): p. 995-1004.https://www.ncbi.nlm.nih.gov/pubmed/33554516
314. Meijer, M.T., et al., *Tenascin C Plasma Levels in Critically Ill Patients with or Without Sepsis: A Multicenter Observational Study.* Shock, 2020. **54**(1): p. 62-69.https://www.ncbi.nlm.nih.gov/pubmed/31764620
315. Yuan, W., et al., *Clinical significance and prognosis of serum tenascin-C in patients with sepsis.* BMC Anesthesiol, 2018. **18**(1): p. 170.https://www.ncbi.nlm.nih.gov/pubmed/30442110
316. Du, Y., et al., *High Serum Secreted Frizzled-Related Protein 5 Levels Associates with Early Improvement of Cardiac Function Following ST-Segment Elevation Myocardial Infarction Treated by Primary Percutaneous Coronary Intervention.* J Atheroscler Thromb, 2019. **26**(10): p. 868-878.https://www.ncbi.nlm.nih.gov/pubmed/30773518
317. Huang, A. and Y. Huang, *Role of Sfrps in cardiovascular disease.* Ther Adv Chronic Dis, 2020. **11**: p. 2040622320901990.https://www.ncbi.nlm.nih.gov/pubmed/32064070
318. Reindl, M., et al., *Association of Myocardial Injury With Serum Procalcitonin Levels in Patients With ST-Elevation Myocardial Infarction.* JAMA Netw Open, 2020. **3**(6): p. e207030.https://www.ncbi.nlm.nih.gov/pubmed/32539151
319. Sinning, C.R., et al., *Association of serum procalcitonin with cardiovascular prognosis in coronary artery disease.* Circ J, 2011. **75**(5): p. 1184-91.https://www.ncbi.nlm.nih.gov/pubmed/21378450

320. Schindler, C., et al., *Role of the vasodilator peptide angiotensin-(1-7) in cardiovascular drug therapy.* Vasc Health Risk Manag, 2007. **3**(1): p. 125-37.https://www.ncbi.nlm.nih.gov/pubmed/17583183
321. Uri, K., et al., *Circulating ACE2 activity correlates with cardiovascular disease development.* J Renin Angiotensin Aldosterone Syst, 2016. **17**(4).https://www.ncbi.nlm.nih.gov/pubmed/27965422
322. Kragstrup, T.W., et al., *Plasma ACE2 predicts outcome of COVID-19 in hospitalized patients.* PLoS One, 2021. **16**(6): p. e0252799.https://www.ncbi.nlm.nih.gov/pubmed/34086837
323. Yang, J., et al., *Insulin-like growth factor binding protein-2: a new circulating indicator of pulmonary arterial hypertension severity and survival.* BMC Med, 2020. **18**(1): p. 268.https://www.ncbi.nlm.nih.gov/pubmed/33019943
324. Barutaut, M., et al., *Insulin-like Growth Factor Binding Protein 2 predicts mortality risk in heart failure.* Int J Cardiol, 2020. **300**: p. 245-251.https://www.ncbi.nlm.nih.gov/pubmed/31806281
325. Lyle, M.A., et al., *Circulating Neprilysin in Patients With Heart Failure and Preserved Ejection Fraction.* JACC Heart Fail, 2020. **8**(1): p. 70-80.https://www.ncbi.nlm.nih.gov/pubmed/31392960
326. Rorth, R., et al., *Comparison of BNP and NT-proBNP in Patients With Heart Failure and Reduced Ejection Fraction.* Circ Heart Fail, 2020. **13**(2): p. e006541.https://www.ncbi.nlm.nih.gov/pubmed/32065760
327. Salah, K., et al., *Prognosis and NT-proBNP in heart failure patients with preserved versus reduced ejection fraction.* Heart, 2019. **105**(15): p. 1182-1189.https://www.ncbi.nlm.nih.gov/pubmed/30962192
328. Askevold, E.T., et al., *The cardiokine secreted Frizzled-related protein 3, a modulator of Wnt signalling, in clinical and experimental heart failure.* J Intern Med, 2014. **275**(6): p. 621-30.https://www.ncbi.nlm.nih.gov/pubmed/24330105
329. Poling, J., et al., *Therapeutic targeting of the oncostatin M receptor-beta prevents inflammatory heart failure.* Basic Res Cardiol, 2014. **109**(1): p. 396.https://www.ncbi.nlm.nih.gov/pubmed/24292852
330. Alabi, A., et al., *Membrane type 1 matrix metalloproteinase promotes LDL receptor shedding and accelerates the development of atherosclerosis.* Nat Commun, 2021. **12**(1): p. 1889.https://www.ncbi.nlm.nih.gov/pubmed/33767172
331. Lyon, C.A., et al., *EC4, a truncation of soluble N-cadherin, reduces vascular smooth muscle cell apoptosis and markers of atherosclerotic plaque instability.* Mol Ther Methods Clin Dev, 2014. **1**: p. 14004.https://www.ncbi.nlm.nih.gov/pubmed/26015951
332. Lyon, C.A., et al., *Soluble N-cadherin overexpression reduces features of atherosclerotic plaque instability.* Arterioscler Thromb Vasc Biol, 2009. **29**(2): p. 195-201.https://www.ncbi.nlm.nih.gov/pubmed/19008530
333. Williams, H., et al., *MMP-7 mediates cleavage of N-cadherin and promotes smooth muscle cell apoptosis.* Cardiovasc Res, 2010. **87**(1): p. 137-46.https://www.ncbi.nlm.nih.gov/pubmed/20139113
334. Asega, A.F., et al., *Cleavage of proteoglycans, plasma proteins and the platelet-derived growth factor receptor in the hemorrhagic process induced by snake venom metalloproteinases.* Sci Rep, 2020. **10**(1): p. 12912.https://www.ncbi.nlm.nih.gov/pubmed/32737331
335. Laragh, J.H. and J.E. Sealey, *The plasma renin test reveals the contribution of body sodium-volume content (V) and renin-angiotensin (R) vasoconstriction to long-term blood pressure.* Am J Hypertens, 2011. **24**(11): p. 1164-80.https://www.ncbi.nlm.nih.gov/pubmed/21938070
336. Katakami, N., *Can soluble receptor for advanced glycation end-product (sRAGE) levels in blood be used as a predictor of cardiovascular diseases?* Atherosclerosis, 2017. **266**: p. 223-225.https://www.ncbi.nlm.nih.gov/pubmed/28923371
337. Dubin, R.F., et al., *Proteomic analysis of heart failure hospitalization among patients with chronic kidney disease: The Heart and Soul Study.* PLoS One, 2018. **13**(12): p. e0208042.https://www.ncbi.nlm.nih.gov/pubmed/30557359
338. deFilippi, C.R. and N.L. Mills, *Rapid Cardiac Troponin Release After Transient Ischemia: Implications for the Diagnosis of Myocardial Infarction.* Circulation, 2021. **143**(11): p. 1105-1108.https://www.ncbi.nlm.nih.gov/pubmed/33720770

339. Ni, L. and X.H.T. Wehrens, *Cardiac troponin I-more than a biomarker for myocardial ischemia?* Ann Transl Med, 2018. **6**(Suppl 1): p. S17.https://www.ncbi.nlm.nih.gov/pubmed/30613592
340. Wu, X., et al., *Role of IGFBP1 in the senescence of vascular endothelial cells and severity of agingrelated coronary atherosclerosis.* Int J Mol Med, 2019. **44**(5): p. 1921-1931.https://www.ncbi.nlm.nih.gov/pubmed/31545483
341. Yeap, B.B., et al., *Associations of IGF1 and IGFBPs 1 and 3 with all-cause and cardiovascular mortality in older men: the Health In Men Study.* Eur J Endocrinol, 2011. **164**(5): p. 715-23.https://www.ncbi.nlm.nih.gov/pubmed/21378090
342. Zheng, W., et al., *Association of Circulating IGFBP1 Level with the Severity of Coronary Artery Lesions in Patients with Unstable Angina.* Dis Markers, 2017. **2017**: p. 1917291.https://www.ncbi.nlm.nih.gov/pubmed/28316362
343. Carbone, C., et al., *Angiopoietin-Like Proteins in Angiogenesis, Inflammation and Cancer.* Int J Mol Sci, 2018. **19**(2).https://www.ncbi.nlm.nih.gov/pubmed/29389861
344. Shen, L., et al., *Association of C1q/TNF-related protein-1 (CTRP1) serum levels with coronary artery disease.* J Int Med Res, 2019. **47**(6): p. 2571-2579.https://www.ncbi.nlm.nih.gov/pubmed/31081425
345. Biscetti, F., et al., *Sortilin levels are associated with peripheral arterial disease in type 2 diabetic subjects.* Cardiovasc Diabetol, 2019. **18**(1): p. 5.https://www.ncbi.nlm.nih.gov/pubmed/30634965
346. Goettsch, C., M. Kjolby, and E. Aikawa, *Sortilin and Its Multiple Roles in Cardiovascular and Metabolic Diseases.* Arterioscler Thromb Vasc Biol, 2018. **38**(1): p. 19-25.https://www.ncbi.nlm.nih.gov/pubmed/29191923
347. Talbot, H., et al., *Regulatory Roles of Sortilin and SorLA in Immune-Related Processes.* Front Pharmacol, 2018. **9**: p. 1507.https://www.ncbi.nlm.nih.gov/pubmed/30666202
348. Gao, J., et al., *Pcsk9 is associated with severity of coronary artery lesions in male patients with premature myocardial infarction.* Lipids Health Dis, 2021. **20**(1): p. 56.https://www.ncbi.nlm.nih.gov/pubmed/34044829
349. Wang, S., et al., *Correlation of serum PCSK9 in CHD patients with the severity of coronary arterial lesions.* Eur Rev Med Pharmacol Sci, 2016. **20**(6): p. 1135-9.https://www.ncbi.nlm.nih.gov/pubmed/27049268
350. Pilarczyk, K., et al., *Placenta growth factor expression in human atherosclerotic carotid plaques is related to plaque destabilization.* Atherosclerosis, 2008. **196**(1): p. 333-340.https://www.ncbi.nlm.nih.gov/pubmed/17157858
351. Keranov, S., et al., *SPARCL1 as a biomarker of maladaptive right ventricular remodelling in pulmonary hypertension.* Biomarkers, 2020. **25**(3): p. 290-295.https://www.ncbi.nlm.nih.gov/pubmed/32248722
352. Regensburger, D., et al., *Matricellular Protein SPARCL1 Regulates Blood Vessel Integrity and Antagonizes Inflammatory Bowel Disease.* Inflamm Bowel Dis, 2021. **27**(9): p. 1491-1502.https://www.ncbi.nlm.nih.gov/pubmed/33393634
353. He, G.A., et al., *The C-terminal domain of canstatin suppresses in vivo tumor growth associated with proliferation of endothelial cells.* Biochem Biophys Res Commun, 2004. **318**(2): p. 354-60.https://www.ncbi.nlm.nih.gov/pubmed/15120609
354. Assadian, S., et al., *p53 inhibits angiogenesis by inducing the production of Arresten.* Cancer Res, 2012. **72**(5): p. 1270-9.https://www.ncbi.nlm.nih.gov/pubmed/22253229
355. Sugiyama, A., et al., *Cathepsin S degrades arresten and canstatin in infarcted area after myocardial infarction in rats.* J Vet Med Sci, 2019. **81**(4): p. 522-531.https://www.ncbi.nlm.nih.gov/pubmed/30726795
356. Mao, K., et al., *Proteomics of extracellular vesicles in plasma reveals the characteristics and residual traces of COVID-19 patients without underlying diseases after 3 months of recovery.* Cell Death Dis, 2021. **12**(6): p. 541.https://www.ncbi.nlm.nih.gov/pubmed/34035220
357. Schwanekamp, J.A., et al., *TGFBI functions similar to periostin but is uniquely dispensable during cardiac injury.* PLoS One, 2017. **12**(7): p. e0181945.https://www.ncbi.nlm.nih.gov/pubmed/28750100

358. Arvidsson, M., et al., *Plasma proteoglycan prolargin in diagnosis and differentiation of pulmonary arterial hypertension.* ESC Heart Fail, 2021. **8**(2): p. 1230-1243.https://www.ncbi.nlm.nih.gov/pubmed/33403810
359. Barallobre-Barreiro, J., et al., *Proteomics analysis of cardiac extracellular matrix remodeling in a porcine model of ischemia/reperfusion injury.* Circulation, 2012. **125**(6): p. 789-802.https://www.ncbi.nlm.nih.gov/pubmed/22261194
360. Barroso, M.C., et al., *Serum insulin-like growth factor-1 and its binding protein-7: potential novel biomarkers for heart failure with preserved ejection fraction.* BMC Cardiovasc Disord, 2016. **16**(1): p. 199.https://www.ncbi.nlm.nih.gov/pubmed/27769173
361. Peng, J.R., et al., *Elevated Levels of Plasma Superoxide Dismutases 1 and 2 in Patients with Coronary Artery Disease.* Biomed Res Int, 2016. **2016**: p. 3708905.https://www.ncbi.nlm.nih.gov/pubmed/27830142
362. Rossignol, M., M.L. Gagnon, and M. Klagsbrun, *Genomic organization of human neuropilin-1 and neuropilin-2 genes: identification and distribution of splice variants and soluble isoforms.* Genomics, 2000. **70**(2): p. 211-22.https://www.ncbi.nlm.nih.gov/pubmed/11112349
363. Roy, S., et al., *Multifaceted Role of Neuropilins in the Immune System: Potential Targets for Immunotherapy.* Front Immunol, 2017. **8**: p. 1228.https://www.ncbi.nlm.nih.gov/pubmed/29067024
364. Takei, S., et al., *Bone morphogenetic protein-4 promotes induction of cardiomyocytes from human embryonic stem cells in serum-based embryoid body development.* Am J Physiol Heart Circ Physiol, 2009. **296**(6): p. H1793-803.https://www.ncbi.nlm.nih.gov/pubmed/19363129
365. David, L., et al., *Bone morphogenetic protein-9 is a circulating vascular quiescence factor.* Circ Res, 2008. **102**(8): p. 914-22.https://www.ncbi.nlm.nih.gov/pubmed/18309101
366. Jeevanandam, M., N.J. Holaday, and S.R. Petersen, *Plasma levels of insulin-like growth factor binding protein-3 in acute trauma patients.* Metabolism, 1995. **44**(9): p. 1205-8.https://www.ncbi.nlm.nih.gov/pubmed/7545263
367. Hoeflich, A., R. David, and R. Hjortebjerg, *Current IGFBP-Related Biomarker Research in Cardiovascular Disease-We Need More Structural and Functional Information in Clinical Studies.* Front Endocrinol (Lausanne), 2018. **9**: p. 388.https://www.ncbi.nlm.nih.gov/pubmed/30061864
368. Saxne, T. and D. Heinegard, *Cartilage oligomeric matrix protein: a novel marker of cartilage turnover detectable in synovial fluid and blood.* Br J Rheumatol, 1992. **31**(9): p. 583-91.https://www.ncbi.nlm.nih.gov/pubmed/1381980
369. Sandstedt, J., et al., *COMP (Cartilage Oligomeric Matrix Protein) Neoepitope: A Novel Biomarker to Identify Symptomatic Carotid Stenosis.* Arterioscler Thromb Vasc Biol, 2021. **41**(3): p. 1218-1228.https://www.ncbi.nlm.nih.gov/pubmed/33472398
370. Marchio, P., et al., *Targeting Early Atherosclerosis: A Focus on Oxidative Stress and Inflammation.* Oxid Med Cell Longev, 2019. **2019**: p. 8563845.https://www.ncbi.nlm.nih.gov/pubmed/31354915
371. Foulquier, S., et al., *WNT Signaling in Cardiac and Vascular Disease.* Pharmacol Rev, 2018. **70**(1): p. 68-141.https://www.ncbi.nlm.nih.gov/pubmed/29247129
372. Zannas, A.S., et al., *Epigenetic upregulation of FKBP5 by aging and stress contributes to NF-kappaB-driven inflammation and cardiovascular risk.* Proc Natl Acad Sci U S A, 2019. **116**(23): p. 11370-11379.https://www.ncbi.nlm.nih.gov/pubmed/31113877
373. Yu, S., et al., *FKBP5 Exacerbates Impairments in Cerebral Ischemic Stroke by Inducing Autophagy via the AKT/FOXO3 Pathway.* Front Cell Neurosci, 2020. **14**: p. 193.https://www.ncbi.nlm.nih.gov/pubmed/32760250
374. Kemppainen, E., et al., *Increased serum trypsinogen 2 and trypsin 2-alpha 1 antitrypsin complex values identify endoscopic retrograde cholangiopancreatography induced pancreatitis with high accuracy.* Gut, 1997. **41**(5): p. 690-5.https://www.ncbi.nlm.nih.gov/pubmed/9414980
375. Wang, J., et al., *Proteomic Signature of Acute Liver Failure: From Discovery and Verification in a Pig Model to Confirmation in Humans.* Mol Cell Proteomics, 2017. **16**(7): p. 1188-1199.https://www.ncbi.nlm.nih.gov/pubmed/28336726

376. Synolaki, E., et al., *The Activin/Follistatin Axis Is Severely Deregulated in COVID-19 and Independently Associated With In-Hospital Mortality.* J Infect Dis, 2021. **223**(9): p. 1544-1554.https://www.ncbi.nlm.nih.gov/pubmed/33625513
377. Sorensen, G.L., *Surfactant Protein D in Respiratory and Non-Respiratory Diseases.* Front Med (Lausanne), 2018. **5**: p. 18.https://www.ncbi.nlm.nih.gov/pubmed/29473039
378. Yi, W., et al., *Paracrine regulation of growth factor signaling by shed leucine-rich repeats and immunoglobulin-like domains 1.* Exp Cell Res, 2011. **317**(4): p. 504-12.https://www.ncbi.nlm.nih.gov/pubmed/21087604
379. Zimmers, T.A., et al., *Growth differentiation factor-15/macrophage inhibitory cytokine-1 induction after kidney and lung injury.* Shock, 2005. **23**(6): p. 543-8.https://www.ncbi.nlm.nih.gov/pubmed/15897808
380. Filbin, M.R., et al., *Longitudinal proteomic analysis of severe COVID-19 reveals survival-associated signatures, tissue-specific cell death, and cell-cell interactions.* Cell Rep Med, 2021. **2**(5): p. 100287.https://www.ncbi.nlm.nih.gov/pubmed/33969320
381. Lampropoulou, E., et al., *Cyclin-dependent kinase 5 mediates pleiotrophin-induced endothelial cell migration.* Sci Rep, 2018. **8**(1): p. 5893.https://www.ncbi.nlm.nih.gov/pubmed/29651006
382. Li, J., et al., *The pro-angiogenic cytokine pleiotrophin potentiates cardiomyocyte apoptosis through inhibition of endogenous AKT/PKB activity.* J Biol Chem, 2007. **282**(48): p. 34984-93.https://www.ncbi.nlm.nih.gov/pubmed/17925408
383. Tsirmoula, S., et al., *Pleiotrophin-induced endothelial cell migration is regulated by xanthine oxidase-mediated generation of reactive oxygen species.* Microvasc Res, 2015. **98**: p. 74-81.https://www.ncbi.nlm.nih.gov/pubmed/25582077
384. Nagano, K., *R-spondin signaling as a pivotal regulator of tissue development and homeostasis.* Jpn Dent Sci Rev, 2019. **55**(1): p. 80-87.https://www.ncbi.nlm.nih.gov/pubmed/31049116
385. Yin, X., et al., *RSPOs facilitated HSC activation and promoted hepatic fibrogenesis.* Oncotarget, 2016. **7**(39): p. 63767-63778.https://www.ncbi.nlm.nih.gov/pubmed/27572318
386. He, B., et al., *Tumor biomarkers predict clinical outcome of COVID-19 patients.* J Infect, 2020. **81**(3): p. 452-482.https://www.ncbi.nlm.nih.gov/pubmed/32504736
387. Vincourt, J.B., et al., *Measurement of matrilin-3 levels in human serum and synovial fluid using a competitive enzyme-linked immunosorbent assay.* Osteoarthritis Cartilage, 2012. **20**(7): p. 783-6.https://www.ncbi.nlm.nih.gov/pubmed/22469847
388. Zafar Gondal, A., L.A. Foris, and J.R. Richards, *Serum Myoglobin*, in *StatPearls*. 2021: Treasure Island (FL).
389. Bodor, G.S., *Biochemical Markers of Myocardial Damage.* EJIFCC, 2016. **27**(2): p. 95-111.https://www.ncbi.nlm.nih.gov/pubmed/27683523
390. Meizlish, M.L., et al., *A neutrophil activation signature predicts critical illness and mortality in COVID-19.* Blood Adv, 2021. **5**(5): p. 1164-1177.https://www.ncbi.nlm.nih.gov/pubmed/33635335
391. Shiota, G., et al., *Serum hepatocyte growth factor levels in liver diseases: clinical implications.* Hepatology, 1995. **21**(1): p. 106-12.https://www.ncbi.nlm.nih.gov/pubmed/7806142
392. Morishita, R., et al., *Hepatocyte growth factor (HGF) as a potential index of severity of hypertension.* Hypertens Res, 1999. **22**(3): p. 161-7.https://www.ncbi.nlm.nih.gov/pubmed/10515437
393. Morishita, R., et al., *Hepatocyte growth factor as cardiovascular hormone: role of HGF in the pathogenesis of cardiovascular disease.* Endocr J, 2002. **49**(3): p. 273-84.https://www.ncbi.nlm.nih.gov/pubmed/12201209
394. Rychli, K., et al., *Hepatocyte growth factor is a strong predictor of mortality in patients with advanced heart failure.* Heart, 2011. **97**(14): p. 1158-63.https://www.ncbi.nlm.nih.gov/pubmed/21572126
395. Liu, Y., et al., *Elevated plasma levels of selective cytokines in COVID-19 patients reflect viral load and lung injury.* National Science Review, 2020. **7**(6): p. 1003-1011.https://doi.org/10.1093/nsr/nwaa037
396. Perreau, M., et al., *The cytokines HGF and CXCL13 predict the severity and the mortality in COVID-19 patients.* Nat Commun, 2021. **12**(1): p. 4888.https://www.ncbi.nlm.nih.gov/pubmed/34373466

397. Okunishi, K., et al., *A novel role of hepatocyte growth factor as an immune regulator through suppressing dendritic cell function.* J Immunol, 2005. **175**(7): p. 4745-53.https://www.ncbi.nlm.nih.gov/pubmed/16177122
398. Molnarfi, N., et al., *Hepatocyte growth factor: A regulator of inflammation and autoimmunity.* Autoimmun Rev, 2015. **14**(4): p. 293-303.https://www.ncbi.nlm.nih.gov/pubmed/25476732
399. Nebigil, C.G., *Updates on Endothelial Functions of Proangiogenic Prokineticin.* Hypertension, 2016. **68**(5): p. 1091-1097.https://www.ncbi.nlm.nih.gov/pubmed/27672031
400. Eggimann, P., Y.A. Que, and F. Rebeaud, *Measurement of pancreatic stone protein in the identification and management of sepsis.* Biomark Med, 2019. **13**(2): p. 135-145.https://www.ncbi.nlm.nih.gov/pubmed/30672312
401. Keel, M., et al., *Pancreatic stone protein is highly increased during posttraumatic sepsis and activates neutrophil granulocytes.* Crit Care Med, 2009. **37**(5): p. 1642-8.https://www.ncbi.nlm.nih.gov/pubmed/19325491
402. Que, Y.A., et al., *Pancreatic stone protein as an early biomarker predicting mortality in a prospective cohort of patients with sepsis requiring ICU management.* Crit Care, 2012. **16**(4): p. R114.https://www.ncbi.nlm.nih.gov/pubmed/22748193
403. Ledda, F., G. Paratcha, and C.F. Ibanez, *Target-derived GFRalpha1 as an attractive guidance signal for developing sensory and sympathetic axons via activation of Cdk5.* Neuron, 2002. **36**(3): p. 387-401.https://www.ncbi.nlm.nih.gov/pubmed/12408843
404. Paratcha, G., et al., *Released GFRalpha1 potentiates downstream signaling, neuronal survival, and differentiation via a novel mechanism of recruitment of c-Ret to lipid rafts.* Neuron, 2001. **29**(1): p. 171-84.https://www.ncbi.nlm.nih.gov/pubmed/11182089
405. Kemik, O., et al., *Serum procarboxypeptidase A and carboxypeptidase A levels in pancreatic disease.* Hum Exp Toxicol, 2012. **31**(5): p. 447-51.https://www.ncbi.nlm.nih.gov/pubmed/21502183
406. Witt, H., et al., *Variants in CPA1 are strongly associated with early onset chronic pancreatitis.* Nat Genet, 2013. **45**(10): p. 1216-20.https://www.ncbi.nlm.nih.gov/pubmed/23955596
407. Khalil, M., et al., *Neurofilaments as biomarkers in neurological disorders.* Nat Rev Neurol, 2018. **14**(10): p. 577-589.https://www.ncbi.nlm.nih.gov/pubmed/30171200
408. Korley, F.K., et al., *Serum NfL (Neurofilament Light Chain) Levels and Incident Stroke in Adults With Diabetes Mellitus.* Stroke, 2019. **50**(7): p. 1669-1675.https://www.ncbi.nlm.nih.gov/pubmed/31138085
409. Uphaus, T., et al., *NfL (Neurofilament Light Chain) Levels as a Predictive Marker for Long-Term Outcome After Ischemic Stroke.* Stroke, 2019. **50**(11): p. 3077-3084.https://www.ncbi.nlm.nih.gov/pubmed/31537188
410. Jockusch, H., G. Friedrich, and M. Zippel, *Serum parvalbumin, an indicator of muscle disease in murine dystrophy and myotonia.* Muscle Nerve, 1990. **13**(6): p. 551-5.https://www.ncbi.nlm.nih.gov/pubmed/2366828
411. Magliozzi, R., et al., *CSF parvalbumin levels reflect interneuron loss linked with cortical pathology in multiple sclerosis.* Ann Clin Transl Neurol, 2021. **8**(3): p. 534-547.https://www.ncbi.nlm.nih.gov/pubmed/33484486
412. Chen, C.C., et al., *Serum pancreas-specific protein in acute pancreatitis. Its clinical utility in comparison with serum amylase.* Scand J Gastroenterol, 1994. **29**(1): p. 87-90.https://www.ncbi.nlm.nih.gov/pubmed/7510410
413. Nie, X., et al., *Multi-organ proteomic landscape of COVID-19 autopsies.* Cell, 2021. **184**(3): p. 775-791 e14.https://www.ncbi.nlm.nih.gov/pubmed/33503446
414. Dencker, M., et al., *Cystatin B, cathepsin L and D related to surrogate markers for cardiovascular disease in children.* PLoS One, 2017. **12**(11): p. e0187494.https://www.ncbi.nlm.nih.gov/pubmed/29149174
415. Goncalves, I., et al., *High levels of cathepsin D and cystatin B are associated with increased risk of coronary events.* Open Heart, 2016. **3**(1): p. e000353.https://www.ncbi.nlm.nih.gov/pubmed/26848396

416. Wehrli, M., et al., *Human IgA Fc receptor FcalphaRI (CD89) triggers different forms of neutrophil death depending on the inflammatory microenvironment.* J Immunol, 2014. **193**(11): p. 5649-59.https://www.ncbi.nlm.nih.gov/pubmed/25339672
417. de Tymowski, C., et al., *CD89 Is a Potent Innate Receptor for Bacteria and Mediates Host Protection from Sepsis.* Cell Rep, 2019. **27**(3): p. 762-775 e5.https://www.ncbi.nlm.nih.gov/pubmed/30995475
418. Vuong, M.T., et al., *Association of soluble CD89 levels with disease progression but not susceptibility in IgA nephropathy.* Kidney Int, 2010. **78**(12): p. 1281-7.https://www.ncbi.nlm.nih.gov/pubmed/20811333
419. Dollt, C., et al., *The shedded ectodomain of Lyve-1 expressed on M2-like tumor-associated macrophages inhibits melanoma cell proliferation.* Oncotarget, 2017. **8**(61): p. 103682-103692.https://www.ncbi.nlm.nih.gov/pubmed/29262593
420. Lim, H.Y., et al., *Hyaluronan Receptor LYVE-1-Expressing Macrophages Maintain Arterial Tone through Hyaluronan-Mediated Regulation of Smooth Muscle Cell Collagen.* Immunity, 2018. **49**(2): p. 326-341 e7.https://www.ncbi.nlm.nih.gov/pubmed/30054204
421. Wong, H.L., et al., *MT1-MMP sheds LYVE-1 on lymphatic endothelial cells and suppresses VEGF-C production to inhibit lymphangiogenesis.* Nat Commun, 2016. **7**: p. 10824.https://www.ncbi.nlm.nih.gov/pubmed/26926389
422. Nishida-Fukuda, H., et al., *Ectodomain Shedding of Lymphatic Vessel Endothelial Hyaluronan Receptor 1 (LYVE-1) Is Induced by Vascular Endothelial Growth Factor A (VEGF-A).* J Biol Chem, 2016. **291**(20): p. 10490-500.https://www.ncbi.nlm.nih.gov/pubmed/26966180
423. Dai, D., et al., *Serum sLYVE-1 is not associated with coronary disease but with renal dysfunction: a retrospective study.* Sci Rep, 2019. **9**(1): p. 10816.https://www.ncbi.nlm.nih.gov/pubmed/31346234
424. Kertesz, N., et al., *The soluble extracellular domain of EphB4 (sEphB4) antagonizes EphB4-EphrinB2 interaction, modulates angiogenesis, and inhibits tumor growth.* Blood, 2006. **107**(6): p. 2330-8.https://www.ncbi.nlm.nih.gov/pubmed/16322467
425. Bisiak, F. and A.A. McCarthy, *Structure and Function of Roundabout Receptors.* Subcell Biochem, 2019. **93**: p. 291-319.https://www.ncbi.nlm.nih.gov/pubmed/31939155
426. Tong, M., et al., *The Role of the Slit/Robo Signaling Pathway.* J Cancer, 2019. **10**(12): p. 2694-2705.https://www.ncbi.nlm.nih.gov/pubmed/31258778
427. Cheng, G., et al., *Identification of PLXDC1 and PLXDC2 as the transmembrane receptors for the multifunctional factor PEDF.* Elife, 2014. **3**: p. e05401.https://www.ncbi.nlm.nih.gov/pubmed/25535841
428. Liu, J., et al., *A new splice variant of the major subunit of human asialoglycoprotein receptor encodes a secreted form in hepatocytes.* PLoS One, 2010. **5**(9): p. e12934.https://www.ncbi.nlm.nih.gov/pubmed/20886072
429. Witzigmann, D., et al., *Variable asialoglycoprotein receptor 1 expression in liver disease: Implications for therapeutic intervention.* Hepatol Res, 2016. **46**(7): p. 686-96.https://www.ncbi.nlm.nih.gov/pubmed/26422581
430. Batulan, Z., et al., *Extracellular Release and Signaling by Heat Shock Protein 27: Role in Modifying Vascular Inflammation.* Front Immunol, 2016. **7**: p. 285.https://www.ncbi.nlm.nih.gov/pubmed/27507972
431. Haider, T., et al., *Systemic release of heat-shock protein 27 and 70 following severe trauma.* Sci Rep, 2019. **9**(1): p. 9595.https://www.ncbi.nlm.nih.gov/pubmed/31270381
432. Chen, D., et al., *GILT restricts the cellular entry mediated by the envelope glycoproteins of SARS-CoV, Ebola virus and Lassa fever virus.* Emerg Microbes Infect, 2019. **8**(1): p. 1511-1523.https://www.ncbi.nlm.nih.gov/pubmed/31631785
433. Rausch, M.P. and K.T. Hastings, *Diverse cellular and organismal functions of the lysosomal thiol reductase GILT.* Mol Immunol, 2015. **68**(2 Pt A): p. 124-8.https://www.ncbi.nlm.nih.gov/pubmed/26116226

434. Nagasawa, R., et al., *Serum heme oxygenase-1 measurement is useful for evaluating disease activity and outcomes in patients with acute respiratory distress syndrome and acute exacerbation of interstitial lung disease.* BMC Pulm Med, 2020. **20**(1): p. 310.https://www.ncbi.nlm.nih.gov/pubmed/33238962
435. Zager, R.A., A.C. Johnson, and K. Becker, *Plasma and urinary heme oxygenase-1 in AKI.* J Am Soc Nephrol, 2012. **23**(6): p. 1048-57.https://www.ncbi.nlm.nih.gov/pubmed/22440905
436. Seta, F., et al., *Heme oxygenase-2 is a critical determinant for execution of an acute inflammatory and reparative response.* Am J Pathol, 2006. **169**(5): p. 1612-23.https://www.ncbi.nlm.nih.gov/pubmed/17071585
437. Sather, S., et al., *A soluble form of the Mer receptor tyrosine kinase inhibits macrophage clearance of apoptotic cells and platelet aggregation.* Blood, 2007. **109**(3): p. 1026-33.https://www.ncbi.nlm.nih.gov/pubmed/17047157
438. Thorp, E., et al., *Shedding of the Mer tyrosine kinase receptor is mediated by ADAM17 protein through a pathway involving reactive oxygen species, protein kinase Cdelta, and p38 mitogen-activated protein kinase (MAPK).* J Biol Chem, 2011. **286**(38): p. 33335-44.https://www.ncbi.nlm.nih.gov/pubmed/21828049
439. Sainaghi, P.P., M. Bellan, and A. Nerviani, *Role of the Gas6/TAM System as a Disease Marker and Potential Drug Target.* Dis Markers, 2021. **2021**: p. 2854925.https://www.ncbi.nlm.nih.gov/pubmed/33532004
440. Salmi, L., et al., *Gas6/TAM Axis in Sepsis: Time to Consider Its Potential Role as a Therapeutic Target.* Dis Markers, 2019. **2019**: p. 6156493.https://www.ncbi.nlm.nih.gov/pubmed/31485279
441. Grubb, A., *Cystatin C is Indispensable for Evaluation of Kidney Disease.* EJIFCC, 2017. **28**(4): p. 268-276.https://www.ncbi.nlm.nih.gov/pubmed/29333146
442. Murty, M.S., et al., *Serum cystatin C as a marker of renal function in detection of early acute kidney injury.* Indian J Nephrol, 2013. **23**(3): p. 180-3.https://www.ncbi.nlm.nih.gov/pubmed/23814415
443. Taglieri, N., W. Koenig, and J.C. Kaski, *Cystatin C and cardiovascular risk.* Clin Chem, 2009. **55**(11): p. 1932-43.https://www.ncbi.nlm.nih.gov/pubmed/19713275
444. Chen, D., et al., *Serum Cystatin C and Coronavirus Disease 2019: A Potential Inflammatory Biomarker in Predicting Critical Illness and Mortality for Adult Patients.* Mediators Inflamm, 2020. **2020**: p. 3764515.https://www.ncbi.nlm.nih.gov/pubmed/33061826
445. Zinellu, A. and A.A. Mangoni, *Cystatin C, COVID-19 severity and mortality: a systematic review and meta-analysis.* J Nephrol, 2021.https://www.ncbi.nlm.nih.gov/pubmed/34390479
446. Yawei, Z., et al., Research Square, 2021.https://doi.org/10.21203/rs.3.rs-610842/v1
447. Li, W. and H. Yue, *Thymidine Phosphorylase Is Increased in COVID-19 Patients in an Acuity-Dependent Manner.* Front Med (Lausanne), 2021. **8**: p. 653773.https://www.ncbi.nlm.nih.gov/pubmed/33829029
448. Liu, C., et al., *Extracellular gamma-synuclein promotes tumor cell motility by activating beta1 integrin-focal adhesion kinase signaling pathway and increasing matrix metalloproteinase-24, -2 protein secretion.* J Exp Clin Cancer Res, 2018. **37**(1): p. 117.https://www.ncbi.nlm.nih.gov/pubmed/29903032
449. Vergara, D., et al., *Proteomic expression profile of injured rat peripheral nerves revealed biological networks and processes associated with nerve regeneration.* J Cell Physiol, 2018. **233**(8): p. 6207-6223.https://www.ncbi.nlm.nih.gov/pubmed/29327509
450. Braga Emidio, N., et al., *Structure, Function, and Therapeutic Potential of the Trefoil Factor Family in the Gastrointestinal Tract.* ACS Pharmacol Transl Sci, 2020. **3**(4): p. 583-597.https://www.ncbi.nlm.nih.gov/pubmed/32832864
451. Lebherz-Eichinger, D., et al., *Increased trefoil factor 2 levels in patients with chronic kidney disease.* PLoS One, 2017. **12**(3): p. e0174551.https://www.ncbi.nlm.nih.gov/pubmed/28355260
452. Samson, M.H., et al., *Circulating trefoil factors in relation to lung cancer, age and lung function: a cross-sectional study in patients referred for suspected lung cancer.* Scand J Clin Lab Invest, 2021. **81**(6): p. 446-450.https://www.ncbi.nlm.nih.gov/pubmed/34242119

453. Osorio-Conles, O., et al., *Adipose tissue and serum CCDC80 in obesity and its association with related metabolic disease.* Mol Med, 2017. **23**: p. 225-234.https://www.ncbi.nlm.nih.gov/pubmed/28850155
454. Wang, H.H., et al., *Plasma asprosin, CCDC80 and ANGPTL4 levels are associated with metabolic and cardiovascular risk in patients with inflammatory bowel disease.* Physiol Res, 2021. **70**(2): p. 203-211.https://www.ncbi.nlm.nih.gov/pubmed/33676388
455. Vuotikka, P., et al., *Serum myoglobin/carbonic anhydrase III ratio as a marker of reperfusion after myocardial infarction.* Int J Cardiol, 2003. **91**(2-3): p. 137-44.https://www.ncbi.nlm.nih.gov/pubmed/14559123
456. Lee, Y., et al., *Testican-1, as a novel diagnosis of sepsis.* J Cell Biochem, 2018. **119**(5): p. 4216-4223.https://www.ncbi.nlm.nih.gov/pubmed/29315764
457. Qu, Y., et al., *Increased trefoil factor 3 levels in the serum of patients with three major histological subtypes of lung cancer.* Oncol Rep, 2012. **27**(4): p. 1277-83.https://www.ncbi.nlm.nih.gov/pubmed/22246423
458. Jia, M., et al., *Ezrin, a Membrane Cytoskeleton Cross-Linker Protein, as a Marker of Epithelial Damage in Asthma.* Am J Respir Crit Care Med, 2019. **199**(4): p. 496-507.https://www.ncbi.nlm.nih.gov/pubmed/30290132
459. Stoyanova, T., et al., *Regulated proteolysis of Trop2 drives epithelial hyperplasia and stem cell self-renewal via beta-catenin signaling.* Genes Dev, 2012. **26**(20): p. 2271-85.https://www.ncbi.nlm.nih.gov/pubmed/23070813
460. Chung, E.J., et al., *Transforming growth factor alpha is a critical mediator of radiation lung injury.* Radiat Res, 2014. **182**(3): p. 350-62.https://www.ncbi.nlm.nih.gov/pubmed/25117621
461. Koch, M., et al., *CD36-mediated activation of endothelial cell apoptosis by an N-terminal recombinant fragment of thrombospondin-2 inhibits breast cancer growth and metastasis in vivo.* Breast Cancer Res Treat, 2011. **128**(2): p. 337-46.https://www.ncbi.nlm.nih.gov/pubmed/20714802
462. Berezin, A.E., A.A. Kremzer, and T.A. Samura, *Circulating thrombospondine-2 in patients with moderate-to-severe chronic heart failure due to coronary artery disease.* J Biomed Res, 2015. **30**.https://www.ncbi.nlm.nih.gov/pubmed/26423730
463. Buda, V., et al., *Thrombospondin-1 Serum Levels In Hypertensive Patients With Endothelial Dysfunction After One Year Of Treatment With Perindopril.* Drug Des Devel Ther, 2019. **13**: p. 3515-3526.https://www.ncbi.nlm.nih.gov/pubmed/31631975
464. Kimura, T., et al., *Serum thrombospondin 2 is a novel predictor for the severity in the patients with NAFLD.* Liver Int, 2021. **41**(3): p. 505-514.https://www.ncbi.nlm.nih.gov/pubmed/33386676
465. Lee, C.H., et al., *Circulating Thrombospondin-2 as a Novel Fibrosis Biomarker of Nonalcoholic Fatty Liver Disease in Type 2 Diabetes.* Diabetes Care, 2021. **44**(9): p. 2089-2097.https://www.ncbi.nlm.nih.gov/pubmed/34183428
466. Qi, L., et al., *Thrombospondin-2 is upregulated in patients with aortic dissection and enhances angiotensin II-induced smooth muscle cell apoptosis.* Exp Ther Med, 2020. **20**(6): p. 150.https://www.ncbi.nlm.nih.gov/pubmed/33093888
467. Schroen, B., et al., *Thrombospondin-2 is essential for myocardial matrix integrity: increased expression identifies failure-prone cardiac hypertrophy.* Circ Res, 2004. **95**(5): p. 515-22.https://www.ncbi.nlm.nih.gov/pubmed/15284191
468. Schweitzer, K.S., et al., *IGSF3 mutation identified in patient with severe COPD alters cell function and motility.* JCI Insight, 2020. **5**(14).https://www.ncbi.nlm.nih.gov/pubmed/32573489
469. Ekman, C., et al., *Plasma concentrations of Gas6 (growth arrest specific protein 6) and its soluble tyrosine kinase receptor sAxl in sepsis and systemic inflammatory response syndromes.* Crit Care, 2010. **14**(4): p. R158.https://www.ncbi.nlm.nih.gov/pubmed/20731857
470. Tsoutsou, P.G., et al., *ICAM-1, ICAM-2 and ICAM-3 in the sera of patients with idiopathic pulmonary fibrosis.* Inflammation, 2004. **28**(6): p. 359-64.https://www.ncbi.nlm.nih.gov/pubmed/16245079

471. Kaur, S., et al., *Elevated plasma ICAM1 levels predict 28-day mortality in cirrhotic patients with COVID-19 or bacterial sepsis.* JHEP Rep, 2021. **3**(4): p. 100303.https://www.ncbi.nlm.nih.gov/pubmed/33997748
472. Muller, W.A., *Mechanisms of transendothelial migration of leukocytes.* Circ Res, 2009. **105**(3): p. 223-30.https://www.ncbi.nlm.nih.gov/pubmed/19644057
473. Koch, A., et al., *Relevance of serum sclerostin concentrations in critically ill patients.* J Crit Care, 2017. **37**: p. 38-44.https://www.ncbi.nlm.nih.gov/pubmed/27621111
474. Munger, J.S., et al., *The integrin alpha v beta 6 binds and activates latent TGF beta 1: a mechanism for regulating pulmonary inflammation and fibrosis.* Cell, 1999. **96**(3): p. 319-28.https://www.ncbi.nlm.nih.gov/pubmed/10025398
475. Devaux, C.A., S. Mezouar, and J.L. Mege, *The E-Cadherin Cleavage Associated to Pathogenic Bacteria Infections Can Favor Bacterial Invasion and Transmigration, Dysregulation of the Immune Response and Cancer Induction in Humans.* Front Microbiol, 2019. **10**: p. 2598.https://www.ncbi.nlm.nih.gov/pubmed/31781079
476. Grabowska, M.M. and M.L. Day, *Soluble E-cadherin: more than a symptom of disease.* Front Biosci (Landmark Ed), 2012. **17**: p. 1948-64.https://www.ncbi.nlm.nih.gov/pubmed/22201848
477. Farkas, I., et al., *CD59 blocks not only the insertion of C9 into MAC but inhibits ion channel formation by homologous C5b-8 as well as C5b-9.* J Physiol, 2002. **539**(Pt 2): p. 537-45.https://www.ncbi.nlm.nih.gov/pubmed/11882685
478. Ghosh, P., et al., *Glycation of the complement regulatory protein CD59 is a novel biomarker for glucose handling in humans.* J Clin Endocrinol Metab, 2014. **99**(6): p. E999-E1006.https://www.ncbi.nlm.nih.gov/pubmed/24628556
479. Vakeva, A., et al., *Detection of a soluble form of the complement membrane attack complex inhibitor CD59 in plasma after acute myocardial infarction.* Scand J Immunol, 2000. **52**(4): p. 411-4.https://www.ncbi.nlm.nih.gov/pubmed/11013013
480. Budding, K., et al., *Soluble CD59 is a Novel Biomarker for the Prediction of Obstructive Chronic Lung Allograft Dysfunction After Lung Transplantation.* Sci Rep, 2016. **6**: p. 26274.https://www.ncbi.nlm.nih.gov/pubmed/27215188
481. Chalupsky, K., et al., *ADAM10/17-dependent release of soluble c-Met correlates with hepatocellular damage.* Folia Biol (Praha), 2013. **59**(2): p. 76-86.https://www.ncbi.nlm.nih.gov/pubmed/23746173
482. Kim, Y.C., et al., *Soluble cMet levels in urine are a significant prognostic biomarker for diabetic nephropathy.* Sci Rep, 2018. **8**(1): p. 12738.https://www.ncbi.nlm.nih.gov/pubmed/30143691
483. Youn, J.C., et al., *Soluble CD93 levels in patients with acute myocardial infarction and its implication on clinical outcome.* PLoS One, 2014. **9**(5): p. e96538.https://www.ncbi.nlm.nih.gov/pubmed/24801400
484. Greenlee, M.C., S.A. Sullivan, and S.S. Bohlson, *Detection and characterization of soluble CD93 released during inflammation.* Inflamm Res, 2009. **58**(12): p. 909-19.https://www.ncbi.nlm.nih.gov/pubmed/19603257
485. Rodriguez, P., et al., *Deletion of delta-like 1 homologue accelerates fibroblast-myofibroblast differentiation and induces myocardial fibrosis.* Eur Heart J, 2019. **40**(12): p. 967-978.https://www.ncbi.nlm.nih.gov/pubmed/29668883
486. Babaknejad, N., et al., *An Overview of FGF19 and FGF21: The Therapeutic Role in the Treatment of the Metabolic Disorders and Obesity.* Horm Metab Res, 2018. **50**(6): p. 441-452.https://www.ncbi.nlm.nih.gov/pubmed/29883971
487. Wojcik, M., et al., *A decrease in fasting FGF19 levels is associated with the development of non-alcoholic fatty liver disease in obese adolescents.* J Pediatr Endocrinol Metab, 2012. **25**(11-12): p. 1089-93.https://www.ncbi.nlm.nih.gov/pubmed/23329754
488. Schaap, F.G., *Role of fibroblast growth factor 19 in the control of glucose homeostasis.* Curr Opin Clin Nutr Metab Care, 2012. **15**(4): p. 386-91.https://www.ncbi.nlm.nih.gov/pubmed/22617565
489. Justet, A., et al., *FGF19, a potential innovative target in Idiopathic Pulmonary Fibrosis?* European Respiratory Journal, 2019. **54**(suppl 63): p. OA2116

490. Lim, S., et al., *Absence of Myostatin Improves Cardiac Function Following Myocardial Infarction.* Heart Lung Circ, 2018. **27**(6): p. 693-701.https://www.ncbi.nlm.nih.gov/pubmed/28690022
491. Duan, R.D., et al., *Identification of human intestinal alkaline sphingomyelinase as a novel ecto-enzyme related to the nucleotide phosphodiesterase family.* J Biol Chem, 2003. **278**(40): p. 38528-36.https://www.ncbi.nlm.nih.gov/pubmed/12885774
492. Duan, R.D., et al., *Effects of ursodeoxycholate and other bile salts on levels of rat intestinal alkaline sphingomyelinase: a potential implication in tumorigenesis.* Dig Dis Sci, 1998. **43**(1): p. 26-32.https://www.ncbi.nlm.nih.gov/pubmed/9508530
493. Prima, V., M. Cao, and S.I. Svetlov, *ASS and SULT2A1 are Novel and Sensitive Biomarkers of Acute Hepatic Injury-A Comparative Study in Animal Models.* J Liver, 2013. **2**(1).https://www.ncbi.nlm.nih.gov/pubmed/23724364
494. Franscini, N., et al., *Critical role of interleukin-1beta for transcriptional regulation of endothelial 6-pyruvoyltetrahydropterin synthase.* Arterioscler Thromb Vasc Biol, 2003. **23**(11): p. e50-3.https://www.ncbi.nlm.nih.gov/pubmed/14551150
495. Jones, J.M., J.C. Morrell, and S.J. Gould, *Identification and characterization of HAOX1, HAOX2, and HAOX3, three human peroxisomal 2-hydroxy acid oxidases.* J Biol Chem, 2000. **275**(17): p. 12590-7.https://www.ncbi.nlm.nih.gov/pubmed/10777549
496. Na, K., et al., *Human plasma carboxylesterase 1, a novel serologic biomarker candidate for hepatocellular carcinoma.* Proteomics, 2009. **9**(16): p. 3989-99.https://www.ncbi.nlm.nih.gov/pubmed/19658107
497. Fuentes-Prior, P., *Priming of SARS-CoV-2 S protein by several membrane-bound serine proteinases could explain enhanced viral infectivity and systemic COVID-19 infection.* J Biol Chem, 2021. **296**: p. 100135.https://www.ncbi.nlm.nih.gov/pubmed/33268377
498. Katsuki, A., et al., *Plasma levels of agouti-related protein are increased in obese men.* J Clin Endocrinol Metab, 2001. **86**(5): p. 1921-4.https://www.ncbi.nlm.nih.gov/pubmed/11344185
499. Argente, J., et al., *One level up: abnormal proteolytic regulation of IGF activity plays a role in human pathophysiology.* EMBO Mol Med, 2017. **9**(10): p. 1338-1345.https://www.ncbi.nlm.nih.gov/pubmed/28801361
500. Gonzalez, A., et al., *Lysosomal integral membrane protein-2: a new player in lysosome-related pathology.* Mol Genet Metab, 2014. **111**(2): p. 84-91.https://www.ncbi.nlm.nih.gov/pubmed/24389070
501. Guo, H., et al., *SCARB2/LIMP-2 Regulates IFN Production of Plasmacytoid Dendritic Cells by Mediating Endosomal Translocation of TLR9 and Nuclear Translocation of IRF7.* J Immunol, 2015. **194**(10): p. 4737-49.https://www.ncbi.nlm.nih.gov/pubmed/25862818
502. Li, X., et al., *The neuropilin-like protein ESDN regulates insulin signaling and sensitivity.* Am J Physiol Heart Circ Physiol, 2016. **310**(9): p. H1184-93.https://www.ncbi.nlm.nih.gov/pubmed/26921437
503. Ferro, E.S., M.C.F. Gewehr, and A. Navon, *Thimet Oligopeptidase Biochemical and Biological Significances: Past, Present, and Future Directions.* Biomolecules, 2020. **10**(9).https://www.ncbi.nlm.nih.gov/pubmed/32847123
504. Banerjee, A., et al., *Modulation of paired immunoglobulin-like type 2 receptor signaling alters the host response to Staphylococcus aureus-induced pneumonia.* Infect Immun, 2010. **78**(3): p. 1353-63.https://www.ncbi.nlm.nih.gov/pubmed/20065029
505. Shiratori, I., et al., *Activation of natural killer cells and dendritic cells upon recognition of a novel CD99-like ligand by paired immunoglobulin-like type 2 receptor.* J Exp Med, 2004. **199**(4): p. 525-33.https://www.ncbi.nlm.nih.gov/pubmed/14970179
506. Zheng, C., et al., *Landscape of Infiltrating T Cells in Liver Cancer Revealed by Single-Cell Sequencing.* Cell, 2017. **169**(7): p. 1342-1356 e16.https://www.ncbi.nlm.nih.gov/pubmed/28622514
507. Mamane, Y., et al., *Posttranslational regulation of IRF-4 activity by the immunophilin FKBP52.* Immunity, 2000. **12**(2): p. 129-40.https://www.ncbi.nlm.nih.gov/pubmed/10714679

508. Martins-da-Silva, A., et al., *Identification of Secreted Proteins Involved in Nonspecific dsRNA-Mediated Lutzomyia longipalpis LL5 Cell Antiviral Response.* Viruses, 2018. **10**(1).https://www.ncbi.nlm.nih.gov/pubmed/29346269
509. Dolegowska, K., et al., *FGF19 subfamily members: FGF19 and FGF21.* J Physiol Biochem, 2019. **75**(2): p. 229-240.https://www.ncbi.nlm.nih.gov/pubmed/30927227
510. Dongiovanni, P., et al., *beta-Klotho gene variation is associated with liver damage in children with NAFLD.* J Hepatol, 2020. **72**(3): p. 411-419.https://www.ncbi.nlm.nih.gov/pubmed/31655133
511. Blanchette-Mackie, E.J., et al., *Perilipin is located on the surface layer of intracellular lipid droplets in adipocytes.* J Lipid Res, 1995. **36**(6): p. 1211-26.https://www.ncbi.nlm.nih.gov/pubmed/7665999
512. Kern, P.A., et al., *Perilipin expression in human adipose tissue is elevated with obesity.* J Clin Endocrinol Metab, 2004. **89**(3): p. 1352-8.https://www.ncbi.nlm.nih.gov/pubmed/15001633
513. Cui, C., et al., *A CD300c-Fc Fusion Protein Inhibits T Cell Immunity.* Front Immunol, 2018. **9**: p. 2657.https://www.ncbi.nlm.nih.gov/pubmed/30498497
514. Foss, S., et al., *TRIM21-From Intracellular Immunity to Therapy.* Front Immunol, 2019. **10**: p. 2049.https://www.ncbi.nlm.nih.gov/pubmed/31555278
515. Miyake, A., et al., *Neucrin is a novel neural-specific secreted antagonist to canonical Wnt signaling.* Biochem Biophys Res Commun, 2009. **390**(3): p. 1051-5.https://www.ncbi.nlm.nih.gov/pubmed/19857465
516. Odermatt, A., et al., *The N-terminal anchor sequences of 11beta-hydroxysteroid dehydrogenases determine their orientation in the endoplasmic reticulum membrane.* J Biol Chem, 1999. **274**(40): p. 28762-70.https://www.ncbi.nlm.nih.gov/pubmed/10497248
517. Liang, J., et al., *The functions and mechanisms of prefoldin complex and prefoldin-subunits.* Cell Biosci, 2020. **10**: p. 87.https://www.ncbi.nlm.nih.gov/pubmed/32699605
518. Watanabe, K., et al., *Characterization of the glycosylphosphatidylinositol-anchor signal sequence of human Cryptic with a hydrophilic extension.* Biochim Biophys Acta, 2008. **1778**(12): p. 2671-81.https://www.ncbi.nlm.nih.gov/pubmed/18930707
519. Kong, Y., et al., *Breast cancer stem cell markers CD44 and ALDH1A1 in serum: distribution and prognostic value in patients with primary breast cancer.* J Cancer, 2018. **9**(20): p. 3728-3735.https://www.ncbi.nlm.nih.gov/pubmed/30405844
520. Herder, C., et al., *A Systemic Inflammatory Signature Reflecting Cross Talk Between Innate and Adaptive Immunity Is Associated With Incident Polyneuropathy: KORA F4/FF4 Study.* Diabetes, 2018. **67**(11): p. 2434-2442.https://www.ncbi.nlm.nih.gov/pubmed/30115651
521. Ahmed, S., et al., *Elevated plasma endocan and BOC in heart failure patients decrease after heart transplantation in association with improved hemodynamics.* Heart Vessels, 2020. **35**(11): p. 1614-1628.https://www.ncbi.nlm.nih.gov/pubmed/32651845
522. Wang, J.B., et al., *An immune checkpoint score system for prognostic evaluation and adjuvant chemotherapy selection in gastric cancer.* Nat Commun, 2020. **11**(1): p. 6352.https://www.ncbi.nlm.nih.gov/pubmed/33311518
523. Liu, B., et al., *Leucine-rich repeat neuronal protein-1 suppresses apoptosis of gastric cancer cells through regulation of Fas/FasL.* Cancer Sci, 2019. **110**(7): p. 2145-2155.https://www.ncbi.nlm.nih.gov/pubmed/31087525
524. Ostermann, G., et al., *JAM-1 is a ligand of the beta(2) integrin LFA-1 involved in transendothelial migration of leukocytes.* Nat Immunol, 2002. **3**(2): p. 151-8.https://www.ncbi.nlm.nih.gov/pubmed/11812992
525. Sobocka, M.B., et al., *Cloning of the human platelet F11 receptor: a cell adhesion molecule member of the immunoglobulin superfamily involved in platelet aggregation.* Blood, 2000. **95**(8): p. 2600-9.https://www.ncbi.nlm.nih.gov/pubmed/10753840
526. Cavusoglu, E., et al., *Association of plasma levels of F11 receptor/junctional adhesion molecule-A (F11R/JAM-A) with human atherosclerosis.* J Am Coll Cardiol, 2007. **50**(18): p. 1768-76.https://www.ncbi.nlm.nih.gov/pubmed/17964041

527. Ong, K.L., et al., *Elevated plasma level of soluble F11 receptor/junctional adhesion molecule-A (F11R/JAM-A) in hypertension.* Am J Hypertens, 2009. **22**(5): p. 500-5.https://www.ncbi.nlm.nih.gov/pubmed/19214165
528. Jin, Y., et al., *Endothelial activation and dysfunction in COVID-19: from basic mechanisms to potential therapeutic approaches.* Signal Transduct Target Ther, 2020. **5**(1): p. 293.https://www.ncbi.nlm.nih.gov/pubmed/33361764
529. Oliva, A., et al., *Role of Serum E-Selectin as a Biomarker of Infection Severity in Coronavirus Disease 2019.* J Clin Med, 2021. **10**(17).https://www.ncbi.nlm.nih.gov/pubmed/34501466
530. Zonneveld, R., et al., *Soluble adhesion molecules as markers for sepsis and the potential pathophysiological discrepancy in neonates, children and adults.* Crit Care, 2014. **18**(2): p. 204.https://www.ncbi.nlm.nih.gov/pubmed/24602331
531. Okajima, K., et al., *Plasma levels of soluble E-selectin in patients with disseminated intravascular coagulation.* Am J Hematol, 1997. **54**(3): p. 219-24.https://www.ncbi.nlm.nih.gov/pubmed/9067501
532. Neri, T., D. Nieri, and A. Celi, *P-selectin blockade in COVID-19-related ARDS.* Am J Physiol Lung Cell Mol Physiol, 2020. **318**(6): p. L1237-L1238.https://www.ncbi.nlm.nih.gov/pubmed/32464083
533. Agrati, C., et al., *The Role of P-Selectin in COVID-19 Coagulopathy: An Updated Review.* Int J Mol Sci, 2021. **22**(15).https://www.ncbi.nlm.nih.gov/pubmed/34360707
534. Bournazos, S., A. Gupta, and J.V. Ravetch, *The role of IgG Fc receptors in antibody-dependent enhancement.* Nat Rev Immunol, 2020. **20**(10): p. 633-643.https://www.ncbi.nlm.nih.gov/pubmed/32782358
535. Rappaport, E.F., et al., *A soluble form of the human Fc receptor Fc gamma RIIA: cloning, transcript analysis and detection.* Exp Hematol, 1993. **21**(5): p. 689-96.https://www.ncbi.nlm.nih.gov/pubmed/8513871
536. Wines, B.D., et al., *Soluble FcgammaRIIa inhibits rheumatoid factor binding to immune complexes.* Immunology, 2003. **109**(2): p. 246-54.https://www.ncbi.nlm.nih.gov/pubmed/12757620
537. Tassi, I. and M. Colonna, *The cytotoxicity receptor CRACC (CS-1) recruits EAT-2 and activates the PI3K and phospholipase Cgamma signaling pathways in human NK cells.* J Immunol, 2005. **175**(12): p. 7996-8002.https://www.ncbi.nlm.nih.gov/pubmed/16339536
538. Murphy, J.J., et al., *A novel immunoglobulin superfamily receptor (19A) related to CD2 is expressed on activated lymphocytes and promotes homotypic B-cell adhesion.* Biochem J, 2002. **361**(Pt 3): p. 431-6.https://www.ncbi.nlm.nih.gov/pubmed/11802771
539. Simmons, D.P., et al., *SLAMF7 engagement super-activates macrophages in acute and chronic inflammation.* bioRxiv, 2020: p. 2020.11.05.368647.https://www.biorxiv.org/content/biorxiv/early/2020/11/05/2020.11.05.368647.full.pdf
540. Kikuchi, J., et al., *Soluble SLAMF7 promotes the growth of myeloma cells via homophilic interaction with surface SLAMF7.* Leukemia, 2020. **34**(1): p. 180-195.https://www.ncbi.nlm.nih.gov/pubmed/31358854
541. Ishibashi, M., et al., *Clinical impact of serum soluble SLAMF7 in multiple myeloma.* Oncotarget, 2018. **9**(78): p. 34784-34793.https://www.ncbi.nlm.nih.gov/pubmed/30410677
542. Shu, T., et al., *Plasma Proteomics Identify Biomarkers and Pathogenesis of COVID-19.* Immunity, 2020. **53**(5): p. 1108-1122 e5.https://www.ncbi.nlm.nih.gov/pubmed/33128875
543. Vollmy, F., et al., *A serum proteome signature to predict mortality in severe COVID-19 patients.* Life Sci Alliance, 2021. **4**(9).https://www.ncbi.nlm.nih.gov/pubmed/34226277
544. Olivier, E., et al., *Fetuin-B, a second member of the fetuin family in mammals.* Biochem J, 2000. **350 Pt 2**: p. 589-97.https://www.ncbi.nlm.nih.gov/pubmed/10947975
545. Saito, N., et al., *Elevated circulating FABP4 concentration predicts cardiovascular death in a general population: a 12-year prospective study.* Sci Rep, 2021. **11**(1): p. 4008.https://www.ncbi.nlm.nih.gov/pubmed/33597568

546. Tu, W.J., et al., *Circulating FABP4 (Fatty Acid-Binding Protein 4) Is a Novel Prognostic Biomarker in Patients With Acute Ischemic Stroke.* Stroke, 2017. **48**(6): p. 1531-1538.https://www.ncbi.nlm.nih.gov/pubmed/28487339

547. Wang, C.P., et al., *Plasma fatty acid-binding protein 4 (FABP4) level is associated with abnormal QTc interval in patients with stable angina and chronic kidney disease.* BMC Cardiovasc Disord, 2019. **19**(1): p. 153.https://www.ncbi.nlm.nih.gov/pubmed/31234795

548. Saharinen, J., et al., *Latent transforming growth factor-beta binding proteins (LTBPs)--structural extracellular matrix proteins for targeting TGF-beta action.* Cytokine Growth Factor Rev, 1999. **10**(2): p. 99-117.https://www.ncbi.nlm.nih.gov/pubmed/10743502

549. Enomoto, Y., et al., *LTBP2 is secreted from lung myofibroblasts and is a potential biomarker for idiopathic pulmonary fibrosis.* Clin Sci (Lond), 2018. **132**(14): p. 1565-1580.https://www.ncbi.nlm.nih.gov/pubmed/30006483

550. Diaz-Alvarez, L. and E. Ortega, *The Many Roles of Galectin-3, a Multifaceted Molecule, in Innate Immune Responses against Pathogens.* Mediators Inflamm, 2017. **2017**: p. 9247574.https://www.ncbi.nlm.nih.gov/pubmed/28607536

551. Wang, W.H., et al., *The role of galectins in virus infection - A systemic literature review.* J Microbiol Immunol Infect, 2020. **53**(6): p. 925-935.https://www.ncbi.nlm.nih.gov/pubmed/31630962

552. Garcia-Revilla, J., et al., *Hyperinflammation and Fibrosis in Severe COVID-19 Patients: Galectin-3, a Target Molecule to Consider.* Front Immunol, 2020. **11**: p. 2069.https://www.ncbi.nlm.nih.gov/pubmed/32973815

553. Oji, V., et al., *Loss of corneodesmosin leads to severe skin barrier defect, pruritus, and atopy: unraveling the peeling skin disease.* Am J Hum Genet, 2010. **87**(2): p. 274-81.https://www.ncbi.nlm.nih.gov/pubmed/20691404

554. Petrackova, A., et al., *Serum protein pattern associated with organ damage and lupus nephritis in systemic lupus erythematosus revealed by PEA immunoassay.* Clin Proteomics, 2017. **14**: p. 32.https://www.ncbi.nlm.nih.gov/pubmed/29026368

555. de Moura, P.R., et al., *Crystal structure of a soluble decoy receptor IL-22BP bound to interleukin-22.* FEBS Lett, 2009. **583**(7): p. 1072-7.https://www.ncbi.nlm.nih.gov/pubmed/19285080

556. Xu, W., et al., *A soluble class II cytokine receptor, IL-22RA2, is a naturally occurring IL-22 antagonist.* Proc Natl Acad Sci U S A, 2001. **98**(17): p. 9511-6.https://www.ncbi.nlm.nih.gov/pubmed/11481447

557. Dudakov, J.A., A.M. Hanash, and M.R. van den Brink, *Interleukin-22: immunobiology and pathology.* Annu Rev Immunol, 2015. **33**: p. 747-85.https://www.ncbi.nlm.nih.gov/pubmed/25706098
