# Supplementary material for: Prognostic tools and candidate drugs based on plasma proteomics of patients with severe COVID-19 complications
Source: Nat Commun. 2022 Feb 17;13:946. doi: 10.1038/s41467-022-28639-4 (PMC8854716; doi:10.1038/s41467-022-28639-4)
Supplement: Supplementary file 3 — Description of Additional Supplementary Files [file 41467_2022_28639_MOESM3_ESM.pdf]

Description of Additional Supplementary Files

Supplementary Data 1. Data of comorbidity for patients admitted to hospital in Qatar with Mild-Moderate vs Severe COVID-19 during the disease peak period (April 2020-May 2020).

Supplementary Data 2. Olink and clinical data for the cohort from Qatar.

Supplementary Data 3. Differentially Expressed Proteins (DEPs) between severe, mild and control cases in the cohort from Qatar.

Supplementary Data 4. (a) Drug-Protein Interactions (DPI) for proteins with > 1.5-fold upregulation in severe versus mild cases. Only FDA approved drugs included. (b) Multi-target Drug-Protein Interactions (DPI) for proteins with > 1.5-fold upregulation in severe versus mild cases. Summary of multi-target drugs.

Supplementary Data 5. Extracted data from the US MGH cohort for validation of the COVID-19 molecular severity score and the 4-marker clinical score.

Supplementary Data 6. (a) Statistical comparison for Figure 6a. (b) Statistical comparison for Figure 6b. Supplementary Data 6. (c) Statistical comparison for Figure 7a and Supplementary Figure 6a.
