# Supplementary material for: Prognostic tools and candidate drugs based on plasma proteomics of patients with severe COVID-19 complications
Source: Nat Commun. 2022 Feb 17;13:946. doi: 10.1038/s41467-022-28639-4 (PMC8854716; doi:10.1038/s41467-022-28639-4)
Supplement: Supplementary file 10 — Reporting summary [file 41467_2022_28639_MOESM10_ESM.pdf]

nature portfolio

Corresponding author(s): Fares Al-Ejeh

Last updated by author(s): Jan 2, 2022

Reporting Summary

Nature Portfolio wishes to improve the reproducibility of the work that we publish. This form provides structure for consistency and transparency in reporting. For further information on Nature Portfolio policies, see our Editorial Policies and the Editorial Policy Checklist.

Statistics

For all statistical analyses, confirm that the following items are present in the figure legend, table legend, main text, or Methods section.

n/a	Confirmed	
☐	☒	The exact sample size (*n*) for each experimental group/condition, given as a discrete number and unit of measurement
☒	☐	A statement on whether measurements were taken from distinct samples or whether the same sample was measured repeatedly
☐	☒	The statistical test(s) used AND whether they are one- or two-sided<br>*Only common tests should be described solely by name; describe more complex techniques in the Methods section.*
☐	☒	A description of all covariates tested
☐	☒	A description of any assumptions or corrections, such as tests of normality and adjustment for multiple comparisons
☐	☒	A full description of the statistical parameters including central tendency (e.g. means) or other basic estimates (e.g. regression coefficient) AND variation (e.g. standard deviation) or associated estimates of uncertainty (e.g. confidence intervals)
☐	☒	For null hypothesis testing, the test statistic (e.g. *F*, *t*, *r*) with confidence intervals, effect sizes, degrees of freedom and *P* value noted<br>*Give P values as exact values whenever suitable.*
☒	☐	For Bayesian analysis, information on the choice of priors and Markov chain Monte Carlo settings
☒	☐	For hierarchical and complex designs, identification of the appropriate level for tests and full reporting of outcomes
☒	☐	Estimates of effect sizes (e.g. Cohen's *d*, Pearson's *r*), indicating how they were calculated

Our web collection on statistics for biologists contains articles on many of the points above.

Software and code

Policy information about availability of computer code

Data collection	Olink NPX Manager (version 2.1.0.224) for Olink Proteomics panels
Data analysis	SPSS (version 26) for clinical data, Cytoscape (version 3.7.2) with String-db (version 11) plugin for network analysis and visualization, DGIdb (version 4.2.0) for drug-gene interactions, MedCalc® (version 12.7) for ROC curve analyses, R packages in RStudio (version 1.2.5) for hierarchical clustering (heatmap.2, in version 3.1.1 of the gplots package), principal component analysis (PCA, prcomp in the Stats R package version 4.1.1), differential expression analysis (Linear Models for Microarray Data, limma, version 3.28.14), volcano plots, gene-ontology biological process (GO-PB) and KEGG pathways enrichment analyses were used through the standalone version of iDEP (version .92), MUVR (version 0.0.975) and Boruta (version 7.0.0) packages in R for variable selection, GraphPad Prism (version 8) for all other data analysis.

For manuscripts utilizing custom algorithms or software that are central to the research but not yet described in published literature, software must be made available to editors and reviewers. We strongly encourage code deposition in a community repository (e.g. GitHub). See the Nature Portfolio guidelines for submitting code & software for further information.

Data

Policy information about availability of data

All manuscripts must include a data availability statement. This statement should provide the following information, where applicable:

- Accession codes, unique identifiers, or web links for publicly available datasets
- A description of any restrictions on data availability
- For clinical datasets or third party data, please ensure that the statement adheres to our policy

All data and clinical information are supplied in Supplementary Data files 1-6. String-db (version 11) available at https://string-db.org/ and DGIdb (version 4.2.0) available at https://www.dgidb.org/

Field-specific reporting

Please select the one below that is the best fit for your research. If you are not sure, read the appropriate sections before making your selection.

- [x] Life sciences
- [ ] Behavioural & social sciences
- [ ] Ecological, evolutionary & environmental sciences

For a reference copy of the document with all sections, see nature.com/documents/nr-reporting-summary-flat.pdf

Life sciences study design

All studies must disclose on these points even when the disclosure is negative.

Sample size	Sample size (50 patients per group and 50 healthy controls) selected based on availability during the pandemic and consulting with similar early studies carrying out proteomics/metabolomics of COVID-19 patients; effect size on COVID-19 plasma proteomics was not clear at the time of collection (April-May 2020) to enable power calculations.
Data exclusions	All data on all patients are included in Supplementary Table 2. The differential expression analysis of plasma proteins only excluded patients whose Olink data did not pass the Olink QC as described in the Methods section.
Replication	This cohort study did not include technical replication. Validation of the findings from our cohort was done in an independent cohort.
Randomization	Randomization design is not relevant to this cohort study as it was an observational study. Patient samples from the three different groups in our cohort were randomized for the Olink assay plates as instructed by the manufacturer to avoid any bias in measurements of protein expression.
Blinding	This cohort study did not involve group allocations, thus blinding is not relevant.

Reporting for specific materials, systems and methods

We require information from authors about some types of materials, experimental systems and methods used in many studies. Here, indicate whether each material, system or method listed is relevant to your study. If you are not sure if a list item applies to your research, read the appropriate section before selecting a response.

Materials & experimental systems

n/a	Involved in the study	
x		Antibodies
x		Eukaryotic cell lines
x		Palaeontology and archaeology
x		Animals and other organisms
	x	Human research participants
x		Clinical data
x		Dual use research of concern

Methods

n/a	Involved in the study	
x		ChIP-seq
x		Flow cytometry
x		MRI-based neuroimaging

Human research participants

Policy information about studies involving human research participants

Population characteristics	SARS-CoV-2 infection and COVID-19 severity were the variables of interest with co-variates including age, sex, ethnicity, co-morbidities, and clinical symptoms. Statistical analyses in the study considered the influence (interactions) of the co-variates of the population when determining the plasma proteins which associate with COVID-19 severity as described in the Methods section.
Recruitment	A cohort of 100 patients (mild-moderate and severe) affected by COVID-19 disease and admitted to Hamad Medical Corporation (HMC) hospitals; tertiary level hospitals in Doha, Qatar, were recruited. Infection was confirmed by positive RT-PCR assays for SARS-CoV-2 from sputum and throat swab with Ct values around 30. Patients with severe COVID-19 were defined as those requiring ICU admissions due to COVID-19 disease or disease complications, while patients with mild-moderate COVID-19 were admitted to community hospitals but did not requiring ICU care. Patients were approached to participate in the study without predetermine criteria other than being adults, confirmed positive with PCR for SARS-CoV-2 infection. Fifty control subjects were recruited at the Clinical Research Center of the Anti-Doping Laboratory Qatar from volunteers identified by Qatar Red Crescent Society, according to the criteria of being healthy, without prior history of confirmed COVID-19 infection diagnosis, normal SpO2%, and vital signs. Individuals with poor cognitive ability, or any past or present medical disease or were not able to consent were excluded. This exclusion is not expected to cause bias or affect the results of the study. Although we attempted to match the control group to the two groups of patients infected with SARS-CoV-2, there were differences in the characteristics of the groups in terms of the co-variates (summarised in Table 1). To mitigate the effect of these differences, our analysis described in the legend of Figure 1 and the Methods and Results sections corrected for these differences. For the infected patients, the time of blood collection was also considered as a co-variate and the analysis of differentially expressed proteins between the severe COVID-19 patients and patients with mild-moderate disease was also corrected for the time between admission and blood collection.
Ethics oversight	The study received IRB approval from the Hamad Medical Corporation (HMC, Doha - Qatar) and was supported by a grant from HMC-Medical Research Council (MRC); approval and fund number MRC-05-003. Written informed consent was obtained from all the participants in the study. Participants were not compensated for participation. The conduct of this study was in accordance with the International Council for Harmonization's Guideline for Good Clinical Practice (ICH-GCP) and the Declaration of Helsinki.

Note that full information on the approval of the study protocol must also be provided in the manuscript.
